# CODARFE: Unlocking the prediction of continuous environmental variables based on microbiome

**DOI:** 10.1093/gigascience/giaf055

**Published:** 2025-06-23

**Authors:** Murilo Caminotto Barbosa, João Fernando Marques da Silva, Leonardo Cardoso Alves, Robert D Finn, Alexandre Rossi Paschoal

**Affiliations:** Department of Computer Science (DACOM), Universidade Tecnológica Federal do Paraná (UTFPR), Campus Cornélio Procópio, 86300-000, Paraná, Brazil; Centro de Inovação, Superbac Biotechnology Solutions, 86975-000, Mandaguari, Brazil; Centro de Inovação, Superbac Biotechnology Solutions, 86975-000, Mandaguari, Brazil; European Molecular Biology Laboratory, European Bioinformatics Institute (EMBL-EBI), Wellcome Genome Campus, Hinxton, Cambridge, CB10 1RQ, UK; Department of Computer Science (DACOM), Universidade Tecnológica Federal do Paraná (UTFPR), Campus Cornélio Procópio, 86300-000, Paraná, Brazil; Rosalind Franklin Institute, Harwell Science and Innovation Campus, Didcot, OX11 0QS, UK

**Keywords:** compositional data analysis, microbiome, prediction, feature selection, machine learning

## Abstract

**Background:**

Despite the surge in microbiome data acquisition, there is a limited availability of tools capable of effectively analyzing it and identifying correlations between taxonomic compositions and continuous environmental factors. Furthermore, existing tools also do not predict the environmental factors in new samples, underscoring the pressing need for innovative solutions to enhance our understanding of microbiome dynamics and fulfill the prediction gap. Here we introduce CODARFE, a novel tool for sparse compositional microbiome predictor selection and prediction of continuous environmental factors.

**Results:**

We tested CODARFE against 4 state-of-the-art tools in 2 experiments. First, CODARFE outperformed predictor selection in 21 of 24 databases in terms of correlation. Second, among all the tools, CODARFE achieved the highest number of previously identified bacteria linked to environmental factors for human data—that is, at least 7% more. We also tested CODARFE in a cross-study, using the same biome but under different external effects, using a model trained on 1 dataset to predict environmental factors on another dataset, achieving 11% of mean absolute percentage error. Finally, CODARFE is available in 5 formats, including a Windows version with a graphical interface, to installable source code for Linux servers and an embedded Jupyter notebook available at MGnify.

**Conclusions:**

Our findings underscore the robustness and broad applicability of CODARFE across diverse fields, even under varying experimental conditions. Additionally, the ability to predict outcomes in new samples allows for the generation of new insights in previously unexplored contexts, providing researchers with a versatile tool.

## Graphical Abstract

**Graphical Abstract:**
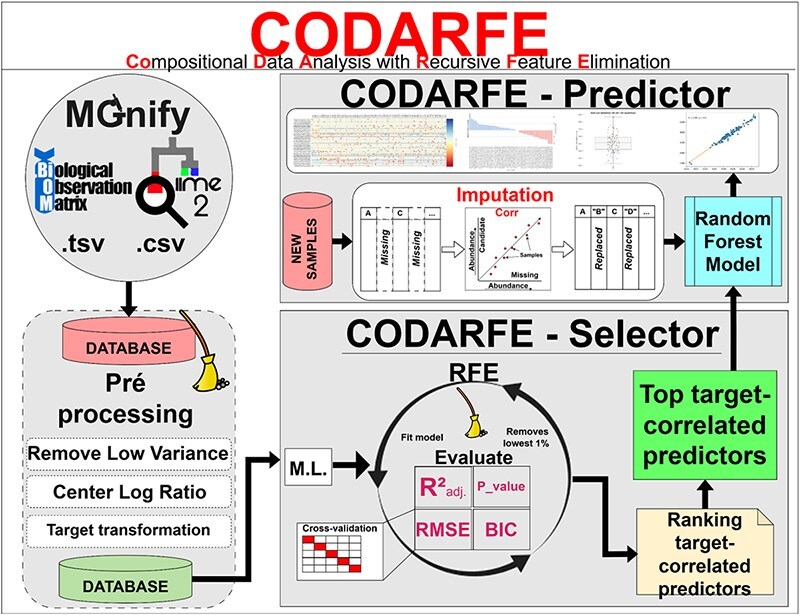
CODARFE’s workflow involves several key steps: Initially, a count table from sequencing data is inputted, alongside a metadata table containing the target variable for association. The data undergo preprocessing to eliminate compositional effects and low-variance predictors. Next, recursive feature elimination (RFE) is applied, iteratively calculating 4 metrics to refine the predictor selection, ensuring the most relevant subset is identified. This refined predictor set is then used to train a random forest model, enabling target prediction in new samples with a novel imputation method to address missing predictors. Outputs include the predictive model, selected predictors, a heatmap of abundance relative to the target distribution, and a bar chart illustrating individual predictor correlations.

**Key Points:**

- CODARFE is a novel tool designed to select sparse compositional microbiome predictors and predict continuous environmental factors, filling a critical gap in microbiome analysis.
- Its predictive power extends across different studies and conditions, achieving 11% mean absolute percentage error in cross-study predictions, showing generalization potential even with varying external effects.
- In testing against 4 state-of-the-art tools, CODARFE outperformed 21 of 24 datasets, showing a stronger correlation and significantly faster performance.
- CODARFE is versatile and available in multiple formats, including a graphical interface for Windows and a Jupyter notebook, and it has potential for use with data types other than 16 s.

## Background

Microbiomes encompass a vast array of microorganisms, including bacteria, viruses, fungi, and protozoa [1], inhabiting diverse environments, including the human body, plants, soils, and even the International Space Station [2]. This microbial diversity has become a focal point of research to understand the role of microbes in health and disease [3–5]. Consequently, this has led to significant interest in identifying specific microbial biomarkers that can serve as indicators for these conditions, providing valuable insights into both the diseases themselves and the environmental factors associated with them [6, 7]. However, comparison between microbiome data coming from 2 or more samples can present specific challenges owing to the composition variability and hence the need to develop appropriate statistical approaches [8, 9].

Analyzing microbial communities typically means measuring the proportions of different microbial taxa present in a sample, but this information is conveyed as ratios or percentages relative to the total microbial community in that sample, creating dependencies among the species proportions [10]. Additionally, data sparsity arises from the presence of rare or low-abundance species that are difficult to detect and quantify [8, 11].

Given these complexities, compositional data analysis (CODA) techniques have emerged as a prominent research focus in the field of microbiome research [12]. With its help, the statistical analysis then can be used to associate the microbiome predictors—in this research, being exclusively taxa—to any target variable [13]. In the context of this research, the “target variable” can be any continuous environmental or clinical variable related to the microbiome.

Although machine learning (ML) techniques have shown promise [14, 15], and their application to microbiome data analysis tools is expanding [16–20], a basic need still exists: the ability to predict the variable of interest in new samples [21]. This challenge arises from the improbability of 2 samples collected in different times—even if they are collected in very close areas—to have the same set of bacterias. To address this challenge, we present CODARFE (COmpositional Data Analysis with Recursive Feature Elimination), a new tool that combines CODA methods with a recursive feature elimination (RFE) process and ML. This approach enables the association of microbiome compositional data with a continuous environmental variable. It also integrates a module for missing predictor imputation and enables the prediction of target environmental variables in new samples.

## Material and Methods

### Evaluation datasets

In this study, we collected a total of 30 datasets from 3 data sources, and for each source, a set of analyses was conducted (Supplementary Fig. S1.1). “Group A: Literature” was collected with the aim of evaluating the coefficient of correlation and processing time required by different methods and for a hold-out prediction evaluation. In total, there are 19 datasets, one for each environmental variable [21]. All 19 tables were preprocessed into count tables, requiring only extraction from the Rdata, where they were stored by the original authors, and transformation into csv files. “Group B: ML Repo” was collected to serve as a positive control. This is due to preexisting evidence of a relationship between 1 or more taxa and the sample metadata variable of interest in the original publication associated with the dataset. The validation is achieved by calculating the number of taxa selected by the tools that are also supported by articles as linked to the target variable. It is composed of 5 human gut datasets obtained from the collection available in the “ML Repo” [22–25], where 2 of these datasets pertain to pediatric Crohn’s disease, with samples collected from the rectum (*n* = 51) and ileum (*n* = 67); 1 from a study on the infant microbiome (aged 0–2 years) (*n* = 49); and 2 from the study one vaginal health, which captured pH (*n* = 388) and Nugent scores (*n* = 388). Similar to Group A, these datasets were already count tables and did not require further preprocessing; were used as they are. “Group C: Cross-Studies” was collected with the purpose to evaluate CODARFE’s generalization power and potential failings when training a model on samples from 1 project and predicting the variable of interest on a different project (from the same biome). Group C consists of 2 sets of datasets extracted from MGnify [26]. The first set comprises 2 projects with pH measurements in arable soil under the effect of a ginseng field and cattle (Table 1) [27, 28] (MGnify identifiers: MGYS00000916 and MGYS00001160). The second set involves age measurements in humans from 4 projects with samples collected from different parts of the digestive system and subjected to various external effects (e.g., antibiotic treatment, HIV) (Table 2) [29–32] (MGnify identifiers: MGYS00000580, MGYS00001175, MGYS00001188, MGYS00001255). Similar to the other 2 groups, all data were already count tables, and no other preprocessing was applied.

**Table 1:** Details of each dataset used in the soil cross-studies analysis

	Chroňáková et al. [27]	Nguyen et al. [28]
**Sample source**	Soil	Soil
**Aim of the study**	Investigation of prokaryotic diversity in soil impacted by outdoor cattle husbandry	Investigation of bacterial communities in the soil of ginseng fields
**Location**	Czech Republic	South Korea
**Year of collection**	2011	2012
**Sequenced region**	V1-V3	V1-V3
**MGnify study identifier**	MGYS00000916	MGYS00001160

**Table 2:** Pertinent data from each research paper used in the human cross-studies analysis

	Di Paola et al. [29]	Raymond et al. [30]	Wang et al. [31]	Noguera-Julian et al. [32]
**Sample source**	Large intestine	Large intestine	Digestive system	Large intestine
**Sample type**	Feces	Feces	Gastric mucosa	Feces
**Aim of the Study**	Characterization of gut Microbiota in juvenile idiopathic arthritis	Study of the impact of antibiotics on the gut microbiome	Microbiota in patients with gastritis, intestinal metaplasia, early gastric cancer, and advanced gastric cancer	Understanding the impact of HIV on the microbiome
**Platform Seq**.	Illumina	Illumina	Illumina	Illumina
**Sequenced region**	V5–V6	V3	V3–V4	16S/18S rRNA
**Host**	Children	Adults between 21 and 35 years old	Adults	Adults
**MGnify study identifier**	MGYS00000580	MGYS00001175	MGYS00001188	MGYS00001255

As a final clarification, all of the experiments were carried out using splits of the same data for training and testing, making them independent of the pipelines used to obtain the count table (as is the tool), with the exception of the cross-studies, where the goal is precisely to understand the effect of different preprocessing pipelines. Supplementary  Tables S1, S2, and S3 summarize the number of samples and columns per dataset per group.

### Data preprocessing

The data preprocessing consists of 2 steps. In the first step, predictors whose variance across samples approximate zero are removed. A value is considered close to zero if it is less than or equal to one-eighth of the mean variance in the dataset, thereby removing columns deemed uninformative [33].

The second step concerns only the target variable, where values are square-rooted and rearranged between 0 and 100 if the coefficient of variation (CV) is greater than or equal to 0.2, indicating a wide variation, which may suggest the presence of noise [34]. The square root serves to mitigate this data dispersion if required, which has been proven effective for reducing errors in machine learning models [35]. Additionally, if the CV is less than 0.2 and the target contains negative numbers, a simple shift is applied to allow the model to predict the target (due to the Poisson distribution used in the model, described later).

### Selection of CODA transformation and regression method

Subsequently, 4 machine learning algorithms were evaluated, using 2 distinct compositional data transformations: Hellinger and center log ratio (CLR) [8]. These transformations were chosen due to their ability to mitigate the inherent issues of compositional data, where the Hellinger transformation is effective for preserving the Euclidean structure of the data [8] and the CLR transformation for its ability to transform compositional data into a real-valued space where traditional statistical methods can be applied without the risk of generating misleading results [13].

The algorithms evaluated were linear support vector regression [36], linear stochastic gradient descent [36], Huber regression [37], and the Theil Sen estimator [38], collectively chosen for their ability to return weights corresponding to the importance of each predictor. Each algorithm was tested with a wide range of hyperparameters and applied to each transformation, termed model hyperparameter transformation (MHT), resulting in 2,270 combinations. Each MHT was evaluated using RFE [39], which removes 1% of the total predictors (columns) with the smallest weights at each iteration, generating up to a hundred trained models.

**Figure 1:**
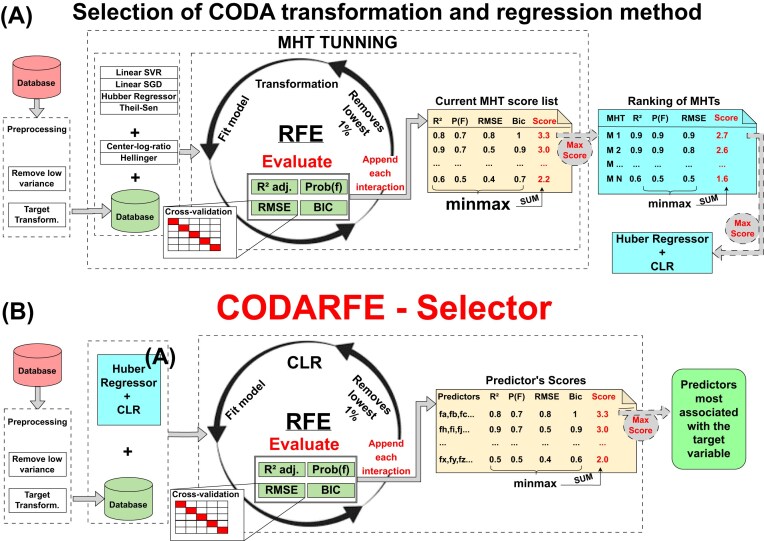
Pipelines from CODARFE selector and selection of CLR and Huber regressor as CODA transformation and regression method. (A) First, the CODARFE selector selects the closely related variables to the target. The data are preprocessed to remove low-variation variables, followed by the RFE, in which the trained model is evaluated using 4 metrics. Finally, the model with the highest “predictors score” (the sum of the “minmax” of the 4 metrics) is chosen, and the variables used to train the model are stated as the most associated with the target. (B) Subsequently, the selection of CODA transformation and regression method is performed. The data are preprocessed to remove low-variance variables, and then RFE is used to train the MHT and evaluate it using 4 metrics. Finally, the MHT with the highest score (the sum of the “minmax” of the 4 metrics) is selected.

Four criteria were used to evaluate each MHT: *R*^2^ adjusted was chosen to determine the correlation strength with the target variable while selecting a concise number of predictors due to its penalization effect, collaborating to reduce the type I error during the fit process. The Bayesian information criterion (BIC) was chosen to differentiate models with a similar *R*^2^ and with different predictor quantities, and it also helps maintain a low type I error due its penalization related to the number of predictors. Root mean squared error (RMSE) was used in cross-validation to reduce overfitting, which is the most important metric since overfitting has the worst impact in a prediction model [40]. Lastly, the *P* value of the *F* test is used to ensure the statistical significance of the selected predictors. All metrics were normalized using the “minmax” method (equation 1) and summed to create the model’s score. The selected MHT is the one with the highest model score. Figure 1A illustrates the complete process.

At the end of the process, the Huber regression yielded the highest scores when paired with the CLR transformation. The “epsilon” parameter, determining outlier influence, was set to 2, and the “alpha” value, controlling regularization, was set at 0.0003. Unfortunately, the CLR influences the nonselected predictors into the selected one due to the geometric mean used in its formula. This problem is mitigated with the use of recursive feature elimination (RFE), where in each interaction, the original data are rescued and retransformed using only the remaining predictors. Furthermore, the RFE helps with issues of data dimensionality, which iteratively reduces the number of columns by filtering for importance.


(1) \begin{eqnarray*}
\textit{minmax}\left( {\textit{data}} \right){\mathrm{\ }} = {\mathrm{\ }}\left( {\textit{data}{\mathrm{\ }} - {\mathrm{\ }}min\left( {\textit{data}} \right)} \right)/\left( {max\left( {\textit{data}} \right) - min\left( {\textit{data}} \right)} \right)\\
\end{eqnarray*}

### CODARFE selector

The predictor selector is the core of the CODARFE. The results of the selector are the predictors used for correlation to the target and the predictors for the prediction model. It starts with the preprocessing step, and then the CLR transformation is applied to the data. The data go under RFE with the Huber regression as an algorithm chosen to give weights (coefficients) to each predictor. The predictors are evaluated following the same process described in the previous section to produce the “Predictors scores.” The set of predictors with the highest score is defined as the predictors most associated with the target variable. Figure 1B depicts the complete process.

### Selection of a predictor algorithm

**Figure 2:**
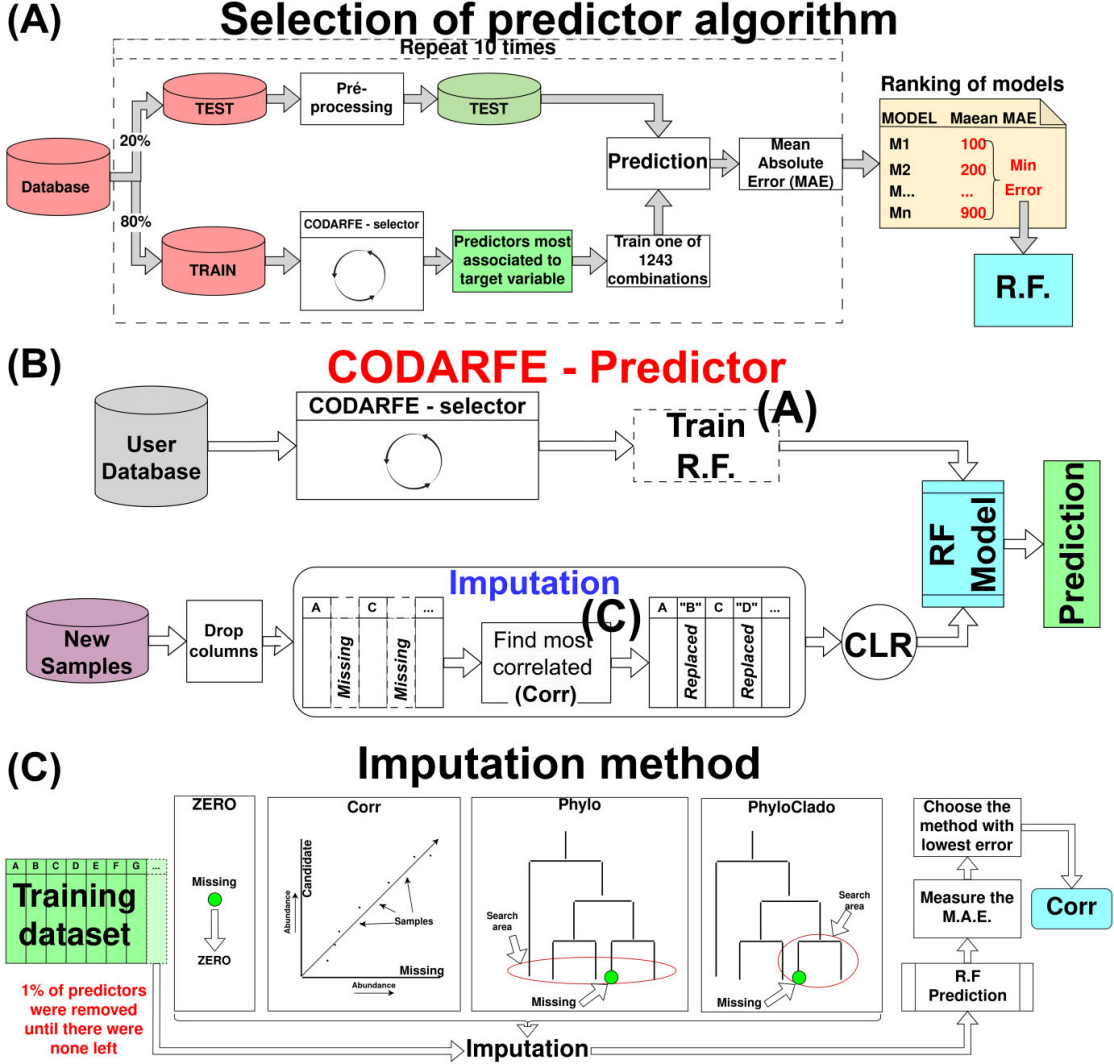
Workflow for CODARFE prediction module and the workflows for selecting random forest (RF) as the prediction algorithm and correlation (Corr) as the imputation method. (A) A view of the process used to select the random forest as a prediction algorithm. First, a training database was split so that 80% of the data were used for training and 20% for testing. Then the attributes most associated with the target variable were select using the CODARFE selector. A total of 1,243 combinations of ML algorithms and hyperparameters were tested, and the MAE was measured with 10 repetitions. As a result, a table of MAEs was created, and the algorithm with the lowest average MAE was selected as a prediction model (RF). (B) The user database is used to select the predictors associated with the target through the CODARFE selector and then training a random forest model. New samples are preprocessed and followed through the imputation step to be used as input for the RF model. The result is the prediction of the new sample’s target variable. (C) The techniques evaluated for replacing (inputting) missing taxa. Four methods were tested in a recursive process where 1% of the total variables from a test dataset was removed in each step, simulating increasing missing predictors. Each imputation method was used to replace the missing taxa, creating a new dataset for the prediction. This new dataset was used to predict the target, and the MAE was measured. The method with the lowest average MAE was selected as the imputation method.

For the predictor algorithm, a new set of regressors was tested: linear support vector regression [36], linear stochastic gradient descent [36], Huber regression [37], Theil-Sen estimator [38], random forest [41], and support vector machine [36] with various kernels, resulting in 1,243 combinations (Fig. 2A). Only the CLR transformation was used. Each model was trained on 80% of the dataset and evaluated on 20% with 10 repetitions to calculate the average of the mean absolute error (MAE) estimations (Fig. 2A). The random forest with parameters “n_estimators = 160” and “criterion = poisson” resulted in a better performance for predictions and was chosen for use in COADRFE.

### CODARFE predictor model

The CODARFE predictor model can only be used after the selector. The selected predictors are used for training a random forest algorithm. When new samples are provided for prediction, they first need to pass through 4 steps. First predictors (columns) that were not selected are removed from data. Then the data go through an imputation method that will replace missing predictors by the most correlated available predictor. The Pearson correlation on the training CLR-transformed data (CODARFE selector) is then measured. If a strongly correlated predictor (ρ ≥ 0.7) is present in the new samples, it will replace the missing one; otherwise, the value is set as zero. Finally, the data are CLR-transformed and predicted by the trained random forest, and the results are obtained (Fig. 2B).

### Imputation method

A missing data imputation method was developed to mitigate the issue of dealing with predictors selected by the model that are not present in a new dataset. Four methods were tested. The first method (“zero”) considered the abundance of the missing predictor as 0. The second method (“corr”) created a list containing “K” possible imputation predictors, where each one had a strong correlation (ρ ≥ 0.7) with the missing one. The Pearson correlation coefficient was calculated from the CLR-transformed data, and the value of “K” was arbitrarily set to 50. The presence of “K” was to increase the chance that at least 1 predictor was available in the new samples. The value of the predictor with the highest correlation found in the new sample is used to substitute the value of the missing predictor. However, this method did not ensure the existence of a correlated attribute, returning zero in such cases. The third method (“phylo”) selected the most similar taxa in a phylogenetic tree generated from the union of the new sample with the training set. The distance between 2 nodes was calculated using the “get-distance” function from the et3 library in Python version 3.10.6. The selected predictor’s value is assigned in place of the missing predictor. The fourth method (“phyloclade”) was similar to the third method, but it was limited to the clade in which the missing attribute was placed. To test each method, the database “aggregate-stability” present in “Group A: Literature” was used. It was divided in an 80–20 scheme where 80% of the data were used for training a random forest and 20% were used for testing. The test data were filtered to contain only the selected predictors. Then, it had 1% of its columns removed to simulate missing predictors. Afterward, each method was used to replace the missing ones, and the random forest model predicted the results. This process was repeated until there were no predictors left. The MAE was used to choose the imputation method, which led to the lowest error rate. Figure 2C illustrates the complete process.

### Comparison against 4 state-of-art tools

A comparison was done between CODARFE and 4 other tools in terms of performance on calculating/extracting/evaluating associations between the microbiome and environmental variables. The following describes the tools and how they were used. These tools represent significant advancements in microbiome analysis, each with its advantages and limitations (Table 3). Every tool, including CODARFE, was trained with its default parameters. No fine tuning was performed for any tool to ensure a fair comparison. BRACoD employs Bayesian regression models [17] to find biological markers of diseases. As recommended by the authors, we used BRACoD with its default settings and filtered the input data to keep only the top 300 most prevalent bacteria. Coda4Microbiome takes into account the inherent variability of count data and uses hierarchical models based on the Bayesian posterior approach [18], while Selbal uses the balances method to identify coabundance patterns among microbiome species [42]. For both tools, all parameters were set to default, and the “nearZeroSum” function (R package “caret”) was used to filter the input data [43]. Finally, CLR-LASSO uses the CLR transformation for Euclidean representation of data and penalizes LASSO regression for variable selection [19]. Nonetheless, it requires the user to set up a lambda value that mimics the number of variables selected by CODARFE as closely as possible.

**Table 3:** Comparisons of tools used in this study for performance benchmark. Each tool has its own mode of operation, which is detailed in this table

	CODARFE	Coda4Microbiome	BRACoD	Selbal	CLR-LASSO
**Filtering method**	RFE	Elastic-Net	MCMC	Balances	LASSO
**Transformation**	CLR	ILR*	CLR	ILR*	CLR
**Limit of columns**	No	No**	300	No**	No
**Create graphics**	Yes	Yes	No	No	No
**Prediction feature**	Yes	No	No	No	No

* Isometric log ratio (ILR).

** Theoretical limit does not exist, but it has a quadratic computational time in relation to the number of columns.

### Simulating CODARFE’s prediction in identical studies

For the simulations, the datasets from Group A were used. For each dataset, 20% of the samples were randomly removed to calculate the MAE, and 80% were used for model training. This procedure was repeated 10 times, and the average MAE was calculated. The accuracy was measured through the mean absolute percentage error (MAPE) (equation 2).


(2) \begin{eqnarray*}
\textit{MAPE} = \frac{{\frac{1}{N}\mathop \sum \nolimits_1^N \left| {rea{l}_i\ - \ pre{d}_i} \right|}}{{\textit{mean}\left( {\textit{target}} \right)}}
\end{eqnarray*}

where, *N*” is the total number of samples, real*_i_* represents the true value in position “*i*,” and pred*_i_* represents the predicted value in position “*i*.”

### Cross-studies prediction simulation

To fairly capture the predictive power, we used the cross-studies approach. We gathered datasets from several projects with different topics but with at least 1 environmental variable that could serve as a prediction target included in their metadata. Then we trained the CODARFE predictor in 1 dataset and used it to predict the target from the other datasets. The predictive power was measured with the following metrics: *R*^2^ values to gauge model generalization, error percentages (equation 2) to quantify predictive accuracy, and the percentage of missing taxa in test datasets that was impossible to replace (imputation method returned zero). This simulation was conducted using the data from “Group C: Cross-Studies,” consisting of 2 subsets, and all taxa were identified by their taxonomy.

The first subset consists of 2 separate soil experiments with different environmental contexts, both of which sequenced identical 16S rRNA variable regions and assessed the pH of the soil. The second subset consists of 4 human studies, each with different primary research objectives and experimental protocols (sequenced 16S rRNA variable regions, data collection techniques, and material type). Nevertheless, in all cases, patient age was present in the metadata, which were used as a prediction target.

### Comparison of computation complexity and running time

To measure the computational time of CODARFE and compare its performance against other tools, a benchmark was conducted using simulated compositional data organized in 2 sets of data, as follows: (i) 1 set contained 100 predictors, while the number of samples varied, and (ii) the other set had a fixed number of samples (*n* = 50), while altering the number of predictors. All experiments were conducted on a Linux Ubuntu 24.04 server with the physical configuration of Xeon E5-2620 v4@2.10 GHz and 192 GB (2,133 MHz DIMM DDR4 Dual Rank) Dell PowerEdge R630. After the fifth day, Coda4Microbiome and Selbal algorithms were prematurely terminated due to excessive time required for each analysis. Only the datasets completed by all tools were considered for comparison.

## Results and Discussion

### CODARFE has a linear runtime, and pure correlation outperforms phylogeny for imputation

**Figure 3:**
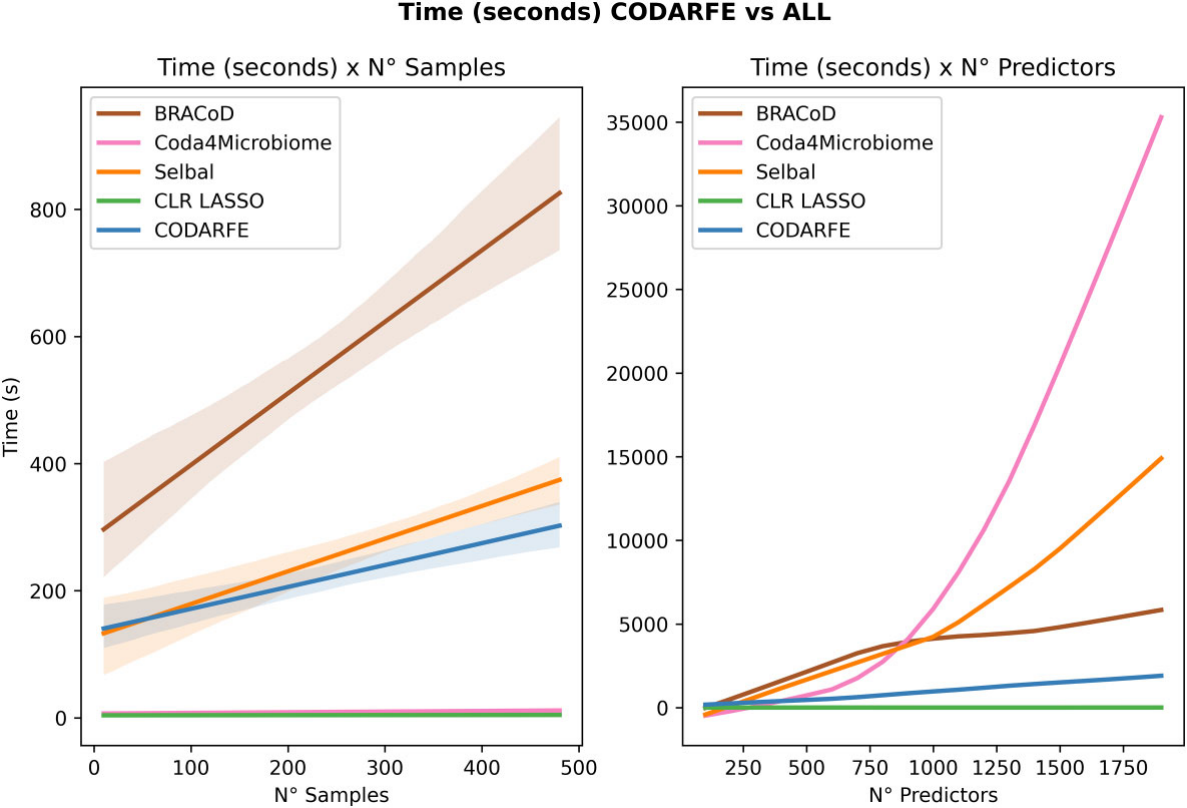
Time consumption comparison between tools regarding number of samples (rows) and number of predictors (columns) in the training dataset. The graph on the left depicts the time in seconds required by each tool to finish its process as the number of samples in the database increases. The graph on the right depicts the time in seconds required by each tool to finish its process as the number of predictors in the database increases.

It is possible to note in Fig. 3 that the computational time regarding the number of samples for BRACOD demonstrated a considerable increase in runtime, while Coda4Microbiome and CLR-LASSO exhibited the lowest runtimes. On the other hand, Selbal had a moderate upswing in runtime, followed closely by CODARFE, but none surpassed 400 seconds (6 minutes) per database.

Regarding the number of predictors, it is clear that Coda4Microbiome and Selbal consume quadratic time (due to data transformation), limiting their usage for datasets with high numbers of predictors (e.g., >1,500 predictors). This can become a problem, since the growth of the number of predictors (columns) is being driven by the development of more data-generating sequencing techniques [44]. BRACoD’s execution time significantly increases with the number of predictors. However, as a “solution,” the authors recommend limiting the dataset to the top 300 most prevalent taxa. This may raise a fundamental issue in the microbiome, which is that the most abundant taxa may not be the most relevant to the target variable [45].

CLR-LASSO has the lowest time in both cases. Nonetheless, it is the simplest approach possible, and interpreting its results can be challenging due to the applied transformation and the effect of the mean of nonselected variables. Furthermore, the user’s choice of the value of lambda directly affects the number of selected predictors, and it lacks generalization capability, as evidenced by previous trials.

Finally, CODARFE grows linearly even when a high number of predictors (e.g., >1,500) are utilized, making it the best choice for largest datasets.

Regarding the imputation method, missing predictors is not a common problem in the field of machine learning, as one of the processes in the traditional ML pipeline is predictor extraction [46]. In this case, the predictors are the taxonomic labels that are used to train the model, but they are not always present in new samples, making the prediction very unstable [47]. Two methods of correlating taxa were considered to try to replace missing predictors (taxa) in new samples: abundance-correlation and the phylogenetic relationship. Although abundance-correlation may have statistical significance, it might lack biological meaning, as having the same abundance does not necessarily mean 2 bacteria have the same biological functions [48]. Additionally, there is no guarantee that highly correlated bacteria will exist in the new sample. On the other hand, the phylogenetic relationship approach is based on the idea that if 2 bacteria are closely related evolutionarily, there is a possibility that they are fulfilling a similar biological role [49]. Although the phylogenetic tree is constructed using both new samples and the training dataset, it is possible that the closest evolutionarily related bacterium may not be “close enough” [50] to have the same function.

**Figure 4:**
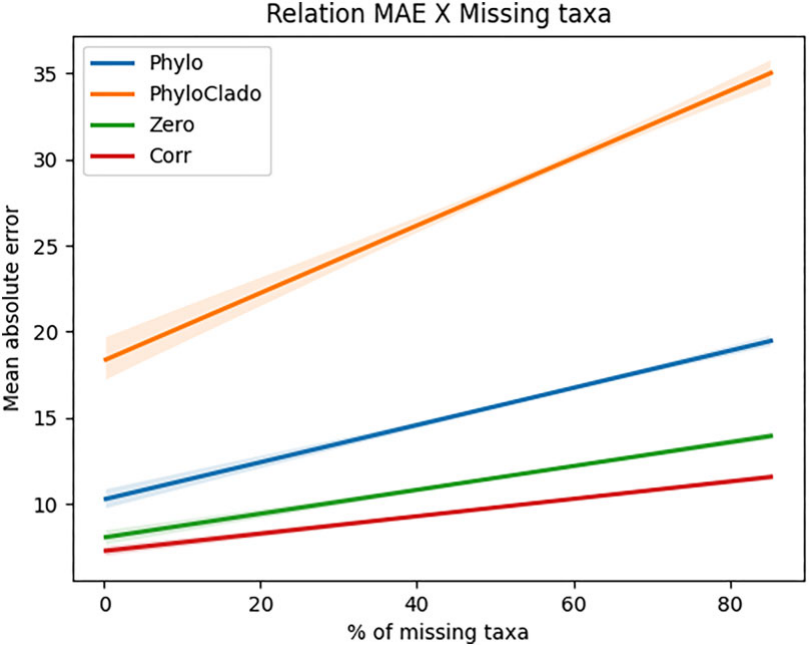
The results of total MAE by percentage of missing taxa for each of the 4 tested imputation methods. The database used for testing was “aggregate-stability” from “Group A: Literature,” with targets ranging from 1.16 to 91.37 units with an average of 21.08 and a standard deviation of 17.48. Starting with all taxa, in each step, 1% of the total taxa are removed until no more taxa are present. In each step, the MAE is calculated for each of the 4 tested imputation methods (Zero, Corr, Phylo, and PhyloClade). The MAE is shown on the y-axis, and the percentage of missing taxa is presented on the x-axis.

As shown in Fig. 4, the abundance-correlation method obtained the lowest mean absolute error concerning the percentage of missing data, making it the chosen imputation method for the tool. Additionally, if more than 75% of the missing bacteria cannot be replaced (not enough correlation), CODARFE immediately stops the prediction.

There may be at least 2 hypotheses for why the correlation method outperformed the others: (i) The chosen bacterium’s abundance value was closer to the original bacterium’s value, which helped the model correctly predict the target variable. Although the biological explanation suggested the use of techniques based on phylogenetic distance, the abundance values between closely related species can be very different. Since the model does not consider the biological aspect and relies solely on abundance values, pure correlation will tend to yield better results. (ii) The second hypothesis is based on the redundancy of biological roles within the microbiome. In this hypothesis, 2 bacteria with a strong correlation of abundance may possess this similarity precisely because they have redundant roles [51].

### CODARFE outperformed other tools regarding data generalization and proportions of correctly selected taxa

**Figure 5:**
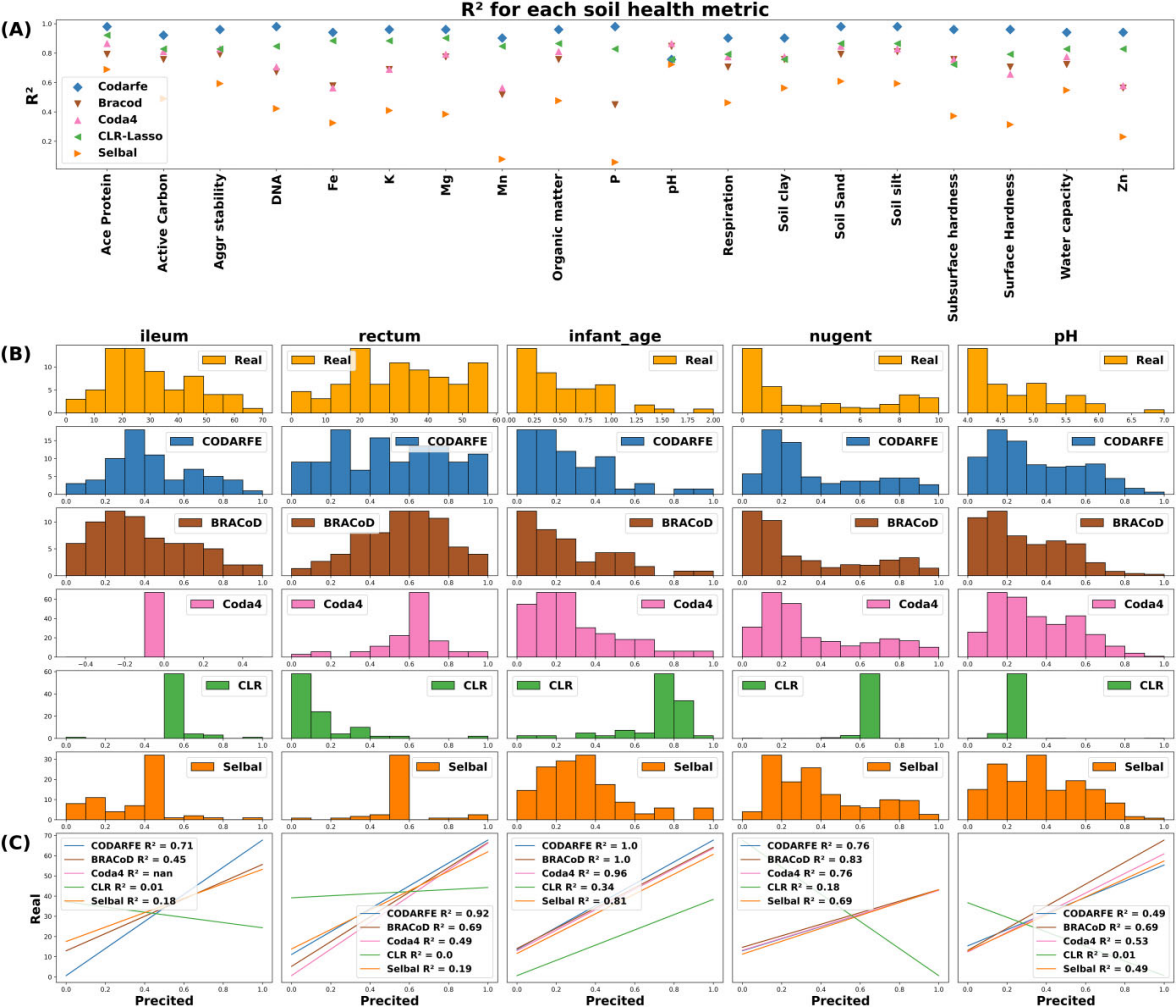
Performance comparison of different machine learning tools for soil and human health prediction. (A) The performance on 19 soil health metrics. CODARFE achieved the best correlation for 18 metrics. BRACoD and Coda4Microbiome had a similar performance, but Coda4Microbiome failed to predict phosphorus (P). Selbal had the worst performance. The complete distribution of the data is available in Supplementary Fig. S1.2. (B) The performance on 5 human disease datasets through histograms of frequency distribution. A distribution similar to the real one indicates good generalization ability of the model. CODARFE outperformed other tools for pediatric Crohn’s disease. Coda4Microbiome failed to predict ileum samples, and CLR-LASSO failed on all datasets. BRACoD and Coda4Microbiome outperformed CODARFE for vaginal health datasets. (C) The R^2^ values for each method on all datasets. CODARFE achieved the best overall performance, except for pH and Nugent score.

Regarding data generalization, 2 groups of datasets were used for benchmarking: 19 soil health metrics (Group A: Literature) and 5 human diseases (Group B: ML Repo). For “Group A: Literature,” CODARFE achieved the best correlation between the generalized and real values for 18 of the 19 measurements. BRACoD and Coda4Microbiome showed similar results, but the latter failed to generalize the amount of phosphorus (“P”), returning “not a number” (nan) during the process. Selbal had the lowest correlation coefficient among all methods, being unable to generalize the data for 4 of the 19 measurements (Fig. 5A). (The complete data distribution is shown in Supplementary Fig. S1.2.)

For “Group B: ML Repo,” CODARFE outperformed the other tools by at least 0.2 in terms of *R*^2^ for the pediatric Crohn’s disease dataset. Coda4Microbiome failed to generalize the data for the samples taken from the ileum, and CLR-LASSO was unable to generalize any of the datasets. Regarding the vaginal health datasets, BRACoD and Coda4Microbiome outperformed CODARFE (Fig. 5).

Except for pH measurements and Nugent scores, CODARFE outperformed all others in the “learned vs. real” correlation. This suggests that the combination of the coefficient shrinkage method with RFE has a higher ability to associate predictors with the target, as previously proposed in [52], where a similar proposal was used and compared with other methods for cervical cancer prediction. Methods primarily based on coefficient shrinkage, such as Coda4Microbiome and Selbal, do not test for subsets smaller than the shrinkage suggested. These subsets may hide combinations of predictors that provide better insights and can be found through more exhaustive tests, such as RFE.

Finally, the lower CODARFE performance in the vaginal pH and Nugent score datasets may be attributed in part to the way the values were measured. Both datasets had 388 samples, but there were only 11 distinct Nugent score values and 7 pH values. Such a low data variation can affect the generalization performance [53] and make the problems more classifiable rather than prone to regression.

To verify the relevance of the taxa selected by the different tools, we performed a more in-depth analysis of the human microbiome data. The 3 publications that were used are each briefly detailed here, and a description of the taxa that were discovered to be connected to the variable of interest is available in Supplementary Material 2 (S2).

**Figure 6:**
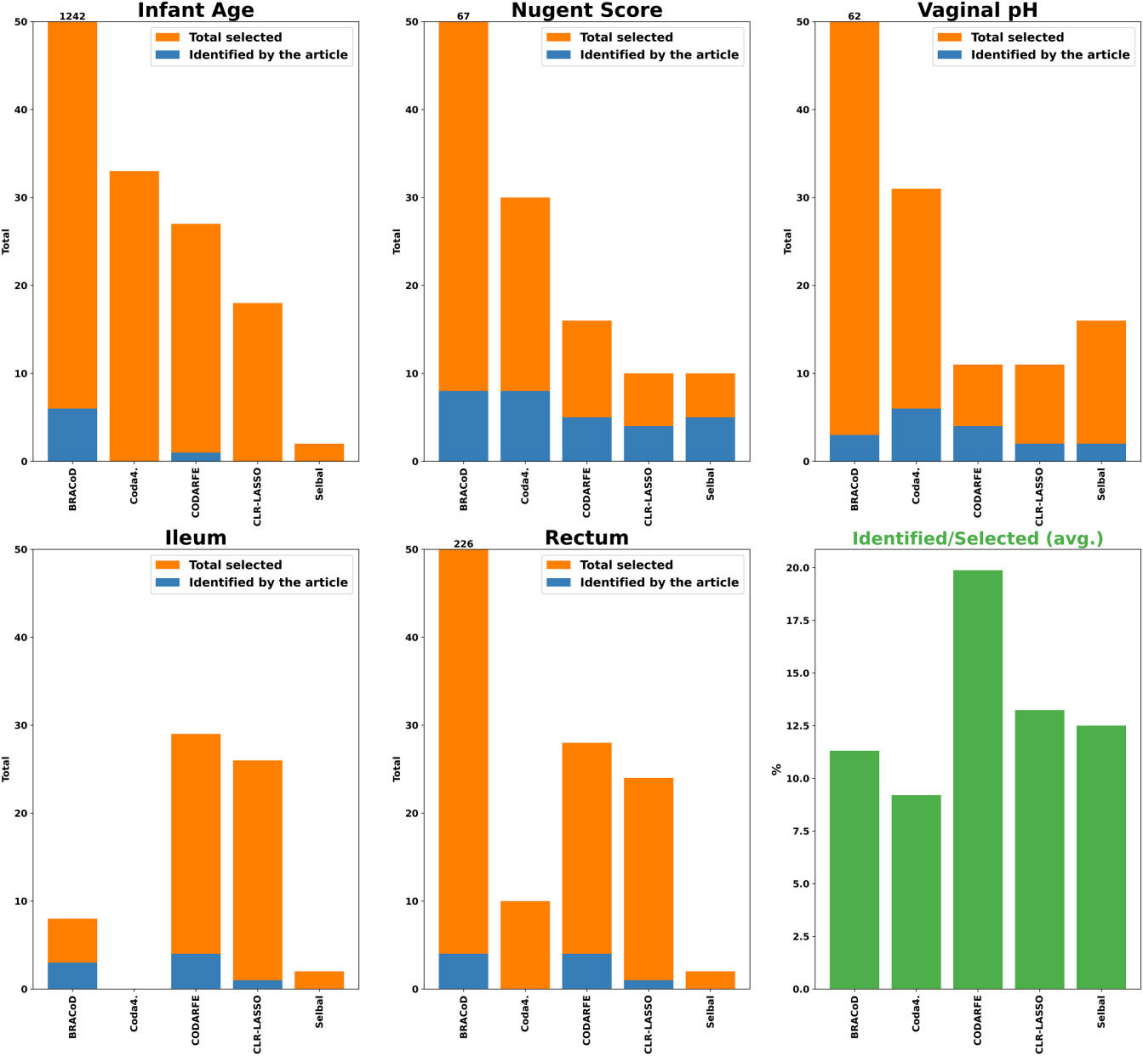
The figure illustrates the performance of all 5 tools for selecting relevant taxa identified in 3 different studies (with 5 different target variables) on the human microbiome. The bars show the number of taxa correctly selected by each tool (blue) and the number of taxa not supported by the original study (orange) for each dataset. CODARFE generally selected more taxa identified in the original studies compared to other tools and also had a lower proportion of false positives (taxa not supported by the studies) compared to other tools, as illustrated by the last panel (green).

Yatsunenko et al. [23] conducted a study utilizing fecal samples from diverse populations, including healthy Amerindians from the Amazon and Venezuela, rural communities in Malawi, and metropolitan areas in the United States. Their aim was to characterize postnatal developmental stages and their correlation with environmental factors. Although the study did not explicitly seek direct associations between the gut microbiome bacteria and postnatal age, *Bifidobacterium* was pointed out by the authors as a potential biomarker for infant age. As shown in green in Fig. 6, only CODARFE and BRACoD selected *Bifidobacterium* species, with CODARFE selecting 41 times fewer false positives than BRACoD. The other selected taxa (red) are not supported by the original article.

Ravel et al. [25] characterized bacteria present in vaginal samples from 4 ethnic groups (White, Black, Hispanic, and Asian) to ascertain vaginal pH and Nugent scores. In total, 20 phylotypes were highlighted as related to the Nugent score, with 19 being related to high levels and 1 to low levels. BRACoD and Coda4Microbiome correctly selected a total of 8 different phylotypes, CODARFE and Selbal correctly selected 5 phylotypes, and CLR-LASSO correctly selected a total of 4 phylotypes (the difference between taxa selected by each tool is described on Supplementary Material 2). For vaginal pH, only the presence or absence of *Lactobacillus* was reported to be relevant, with its presence being inversely proportional to the pH measurement. BRACoD selected 3 species, Coda4Microbiome selected 6 species, CODARFE selected 4 species, CLR-LASSO selected 2 species, and finally Selbal selected 2 species. It is worth noting that the proportions of correctly selected and total selected vary drastically between tools (Fig. 6).

The work developed by Gevers et al. [24] characterized bacteria present in ileum and rectal samples to understand recent cases of pediatric Crohn’s disease through the Paediatric Crohn’s Disease Activity Index (PCDAI). The authors identified 21 species with a high correlation to PCDAI. The results in Fig. 6 are shown for 2 separate datasets (ileum and rectum), but all species are valid for both datasets. BRACoD correctly selected 3 species for the ileum dataset and 3 species for rectum dataset. As previously described, Coda4Microbiome was unable to finish its process for the ileum dataset and did not select any of the 21 species pointed out by the article in the rectum dataset. CODARFE correctly selected 4 species for the ileum dataset and 4 species for the rectum dataset. CLR-LASSO selected only 1 for ileum and rectum, and Selbal did not select any of the 21 species.

In these 3 cases, CODARFE found more species highlighted in the original articles compared to other tools (green bars in Fig. 6), suggesting that it has a lower false discovery rate (FDR) than the other tools on average. We believe that CODARFE was able to achieve these results due to the combination of metrics with the RFE [54]. Two of the metrics, *R*^2^ adjusted and the BIC, are strong indications of a high quantity of predictors [55]. Both of these metrics serve as penalization measures for the number of predictors selected, giving high scores as the number of predictors decrease. In addition, we combined them with the ρ-value of the *F* test in the final predictors score. This value basically indicates how beneficial the inclusion of a predictor is to the model (it can be thought of as a value representing “how much these predictors explain the target”). Since these 3 metrics are weighted and summed together, the final score will indicate “how worthwhile it is to keep all these predictors in relation to how much they explain.” This value is weighted with an “overfitting value” and used to evaluate all (at most) 100 predictor sets during the RFE step, increasing the chance of finding a subset of predictors with optimal scores that are not explored by other methods (such as shrinkage used by Coda4Microbiome) [39].

### Simulating prediction in identical and cross-studies

**Figure 7:**
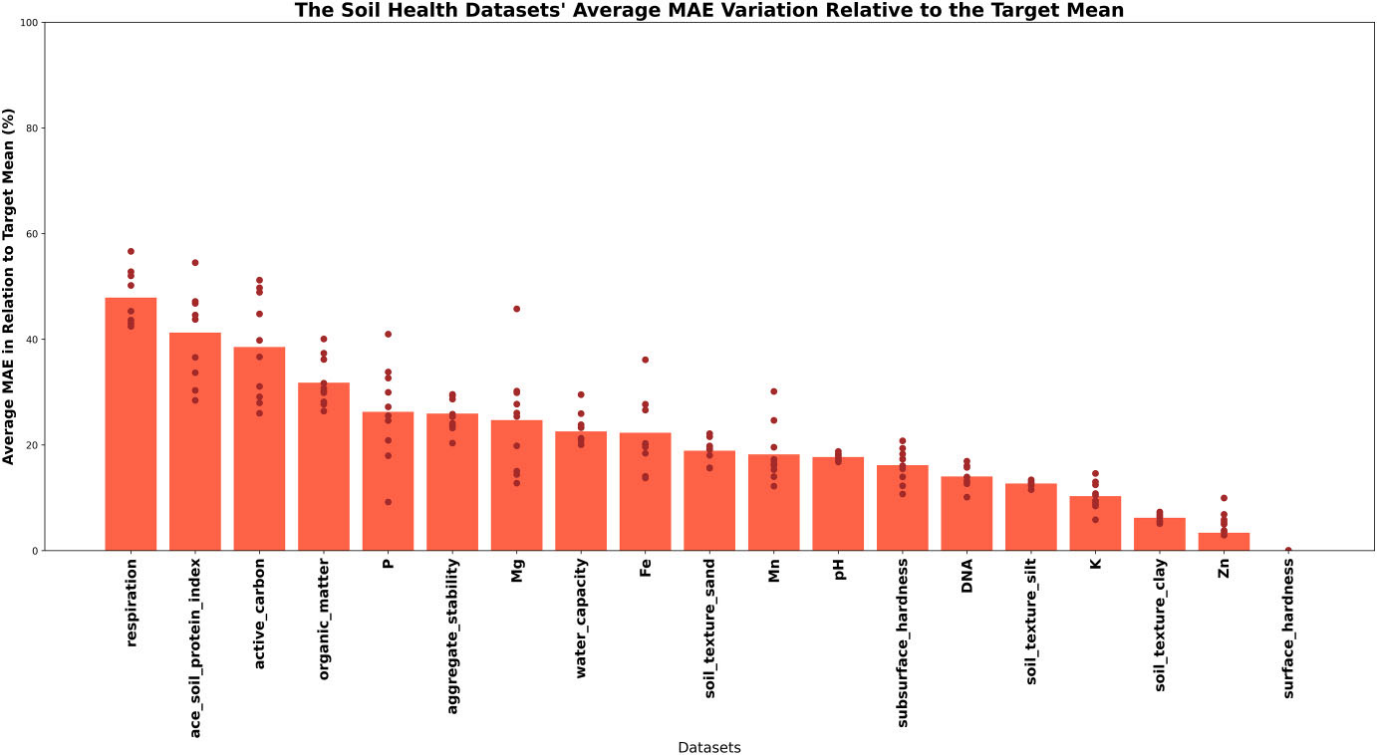
Mean absolute percentage error distribution for soil health and fertility metrics. Individual values across a 10-repetition hold-out validation are represented by the dots, and the average is indicated as the bar. It is possible to see a trend related to the target amplitude, where a narrow distribution has the highest error rate (“respiration”), and a wide distribution has the lowest error (“surface_hardness”).

The next test involved simulating studies through the hold-out method to simulate predictions. Equation 2 was used to calculate the MAPE, which expresses the error as a percentage of each target variable’s mean. Using data from “Group A: Literature” (Fig. 7), “surface hardness” resulted in the lowest error of 0.01% of the target’s mean (target range: 0.001–743.126), followed by “Zn” with 3.37% (target range: 0.001–23.617) and “soil-texture clay” with 6.21% (target range: 0.356–39.436). The highest error was found for “respiration” with 47.88% (target range: 0.077–1.781).

One hypothesis for this arising pattern is related to the data distribution. The “respiration” metric had the narrowest distribution of all the metrics (Supplementary Fig. S1.3), varying only 1.7 units from minimum to maximum with a standard deviation of 0.23 units, making any deviation from the true value to result in a high error. On top of that, the narrow amplitude has high peaks of density, which may create a bias in the model, resulting in even higher errors when the true value is outside the high density range [55]. In contrast, the “surface_hardness” had one of the most widely distributed distributions, ranging from 0 to over 700 units with a standard deviation of 105 units. Even with all these difficulties, most of the results obtained were superior (in terms of *R*^2^) to those reported in the original article [21], supporting the high predictive power of CODARFE in most of the scenarios.

**Figure 8:**
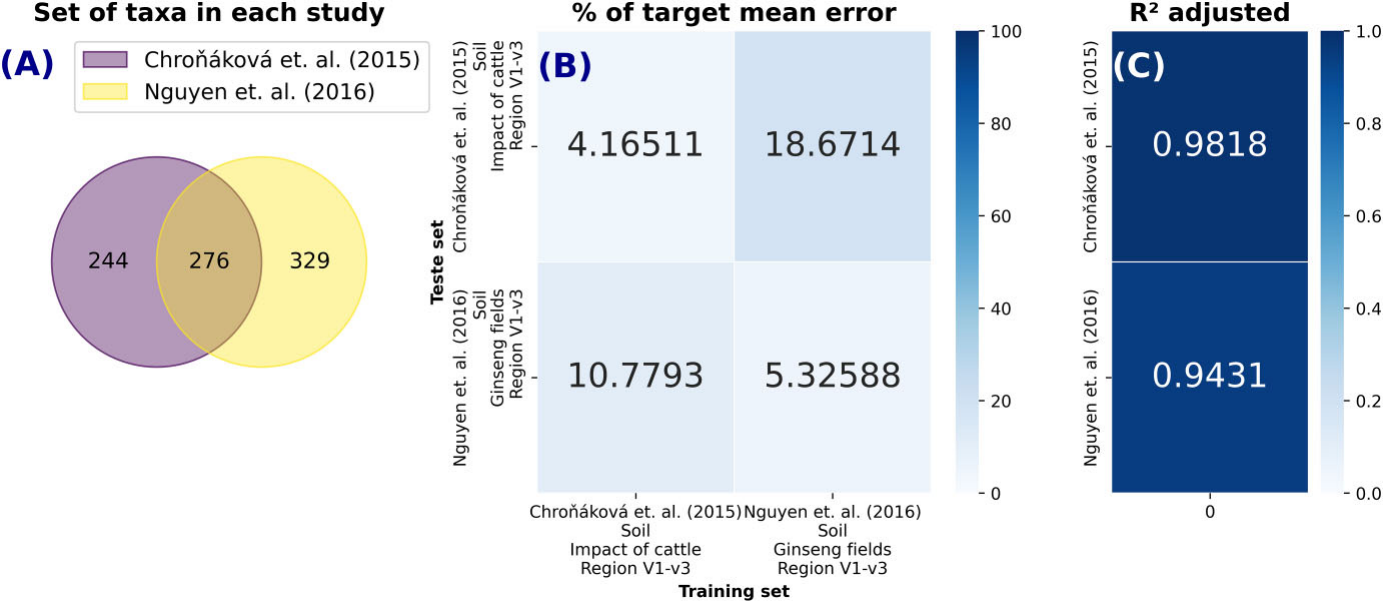
Cross-studies prediction for tow soil datasets measuring pH. The number of identical predictors in each study is shown in (A). The percentage of mean absolute error in relation to the average of the target variable when training CODARFE with data from 1 work and predicting another is shown in (B). The coefficient of determination (*R*^2^) obtained in the generalization of the CODARFE model is shown in (C).

**Figure 9:**
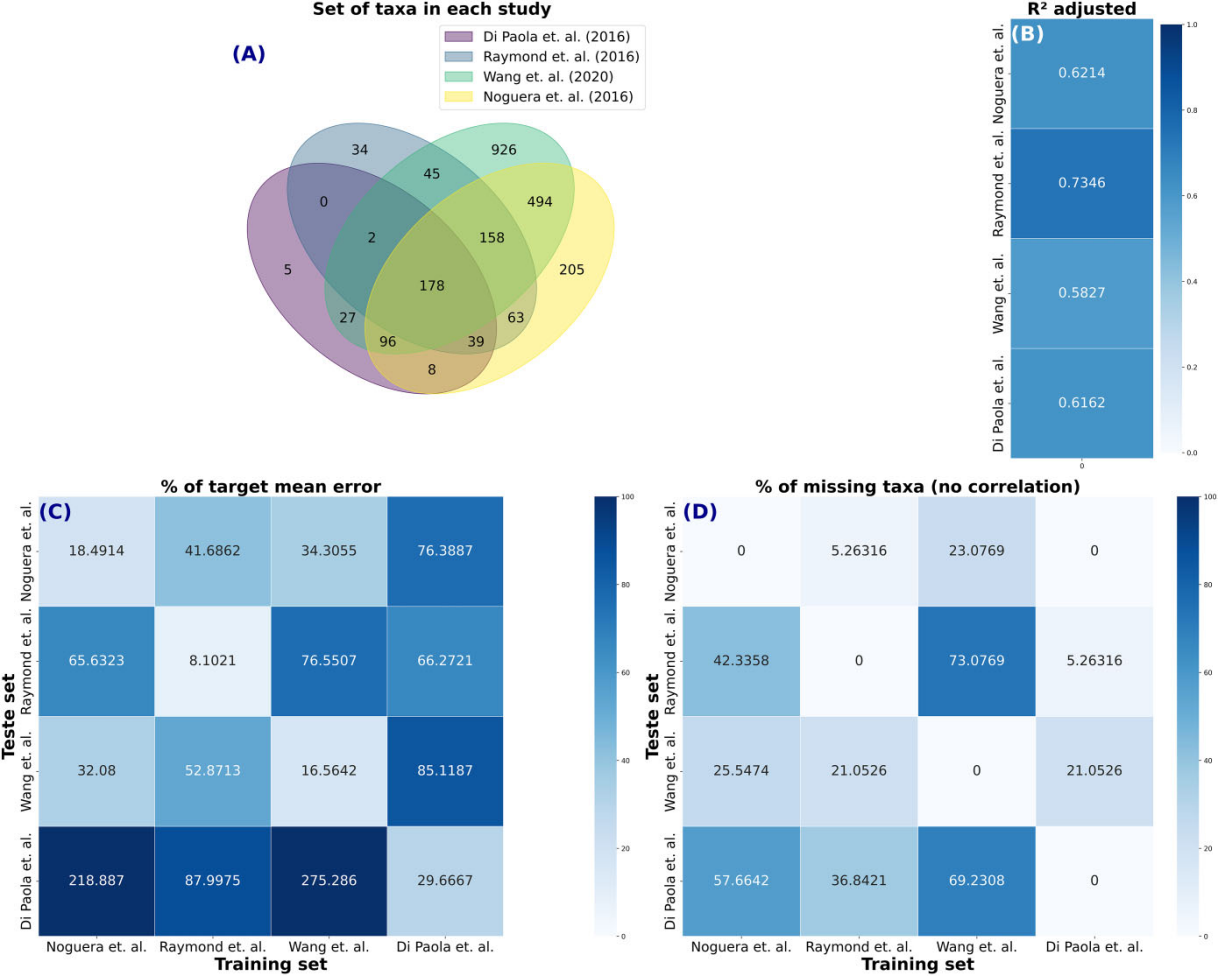
Cross-studies prediction for 4 very distinct human datasets measuring human age. The number of identical predictors (taxa) in each study is shown in (A). The coefficient of determination (*R*^2^) obtained in the generalization of the CODARFE model is shown in (B). The percentage of mean absolute error in relation to the average of the target variable when training CODARFE with data from 1 work and predicting another is shown in (C). The percentage of predictors in the prediction dataset that were unable to be replaced by the imputation method (the imputation method returned zero) is shown in (D).

For the cross-studies prediction, we separated “Group C: Cross-Studies” into 2 groups, one similar to each other and one highly different, to better examine the effects of studies that were similar and studies that were quite different in many areas, such as subject of investigation, region of sequencing, data collection, external effects, preprocessing pipelines, and others (the differences between each dataset are indicated in Tables 1 and 2). We used the *R*² values to judge model generalization (Figs. 8C and 9B), MAPE to quantify predictive accuracy (Figs. 8B and 9C), and the percentage of missing taxa in test datasets (imputation method returned zero) to verify the effect of missing taxa in the final prediction (Fig. 9D).

In the first subset, Chroňáková et al. [27] evaluated the response of soil bacterial communities to changes associated with outdoor cattle overwintering, while Nguyen et al. [28] analyzed how bacterial diversity and community structure in Korean ginseng fields were altered by cultivation time. Both datasets achieved a high *R*^2^ (Fig. 8C) which indicates a high predictive power. For the Chroňáková et al. [27] dataset, the error percentages were 4.17% and 10.78% (Fig. 8B) when tested on itself and the Nguyen et al. [28] dataset, respectively. Similarly, Nguyen et al. [28] exhibited error percentages of 18.67% and 5.33% (Fig. 8B) when tested on the Chroňáková et al. [27] dataset and itself, respectively.

Both the Chroňáková et al. [27] and Nguyen et al. [28] datasets were generated from the 16S rRNA V1–V3 region (Table 1), and all the predictors (taxa) selected by CODARFE were present in both studies (in the intersection, Fig. 8A), meaning that no imputation was needed and reinforcing the idea that pH-related taxa are similar for various types of soils, as described in [56]. This may have helped reduce the error even when the agent of the study effect is entirely different and the soil samples are at least 8,000 km apart.

On top of that, the sequenced region directly influences the assigned taxonomy [57], which is then used as a predictor by the tool. In this case, if different taxonomies are assigned to the same predictor, they will be considered different predictors. As a result, it is prudent to assume that a key factor influencing the observed prediction errors may be the choice of amplified region, since both the Chroňáková et al. [27] and Nguyen et al. [28] datasets were generated from the V1–V3 region (Table 1). The effect of this factor is better observed in the second subset.

In the second subset, 4 completely different studies were used (Table 2), with the human age as the target.

The best results yielded an error of 32% of the mean (Fig. 9C), indicating a low predictive power between studies. A reduced *R*^2^ in these studies suggested that the microbiota may be more influenced by the study object (disease) than by age. Furthermore, although it had a 25% missing taxa rate (Fig. 9D), it is not only the absence of taxa that affects the error rate. As shown by Di Paola et al. [29], the model was unable to predict the age accurately in other works, despite containing most taxa (maximum of 21% missing). This may be partially explained by the fact that this work had the lowest fitting power (*R*^2^) (Fig. 9B) among all, leading us to believe that the error may be a combination of fitting power with missing taxa and sequencing region.

The influence of missing taxa becomes evident when observing the models trained on datasets with the highest missing taxa rate, such as the Wang et al. [31] and Noguera-Julian et al. [32] models predicting the data of Di Paola et al. [29]. The percentage of missing taxa in the Di Paola et al. [29] dataset (Fig. 9D) is substantially higher compared to the training datasets. The Wang et al. [31] model, trained on V3–V4 region, and the Noguera-Julian et al. [32] model, trained on 16S/18S rRNA (Table 2), might not have adequately captured predictors relevant to the V5–V6 region utilized by the Di Paola et al. [29] dataset (Fig. 9A, D). This may suggest a critical relationship between missing taxa and predictive accuracy.

Further examination of the sequencing regions used to generate the data suggested that different regions can impact predictive performance. For instance, the Noguera-Julian et al. [32] model, trained on 16S/18S rRNA data (Table 2), exhibits relatively strong cross-dataset performance, while the Raymond et al. [30] model, trained on the V3 region (Fig. 9C), showcases the highest *R*^2^ value and lowest error on average. Another indication that the sequenced region is an essential factor in the tool’s proper functioning comes from the cross-study analyses of Di Paola et al. [29] and Wang et al. [31]. These 2 datasets did not share the 16S sequenced region and resulted in the highest error rates. Conversely, the models created from Wang et al. [31] and Raymond et al. [30], both sequencing the V3 region, when used to predict the Noguera-Julian et al. [32] study, which sequenced the complete 16S, yielded errors of 34% and 41%, respectively, which are relatively low compared to its other case with a 275% error. Finally, it is fair to assume that the preprocessing pipeline used to generate the count table (including the taxonomy assignment and databases versions) has a deep impact on the final results if 2 different ones are used combined (e.g., train and prediction).

Regarding the batch effect in this cross-study analyses, CODARFE completely avoided it since the models were trained on a single dataset without batch mixing. The batch effect—particularly its most severe manifestation—arises when different experimental batches are combined, creating a spurious correlation between the batch and the target variable. Notably, the pH model selected only bacterial taxa present in both datasets, even without their mixing, suggesting that CODARFE successfully identified bacteria specifically associated with pH. Additionally, any batch effects inherent to a single dataset would affect all samples within that dataset uniformly, rendering them less informative. Consequently, such effects are likely to be eliminated during the preprocessing phase.

### Feasibility and limitations

Based on the results obtained, we present 8 hypotheses regarding CODARFE feasibility and limitations:

- The main factor determining predictive power is the number of predictors that could not be replaced by the suggested imputation method, as observed in the models trained with the Wang et al. [31] or Noguera-Julian et al. [32] datasets and tested in the Di Paola et al. [29] and Raymond et al. [30] datasets. These models had the highest associated error and the highest percentage of missing predictors. Considering such a limitation, it is likely that the sequenced region needs to be the same for better results since the sequenced region directly affects the assigned taxonomy. Furthermore, we made CODARFE automatically stop the prediction if the number of missing taxa was greater than 75%.
- As long as 2 distinct pipelines—including database versions used for taxonomy assignment—are not combined (e.g., train and prediction), CODARFE remains agnostic of the preprocessing pipeline used to create the count table. It is reasonable to believe, based on the results, that there would be no harm if the same pipeline were utilized for the collection and analysis of additional samples. However, the prediction power significantly decreases when using different pipelines. Considering that the data used to “test” the model differ from that used to train it, this behavior can be expected and presents challenges for any machine learning problem.
- The samples from which the sequences are extracted should be similar, as the microbiome is closely linked to the environment. Thus, samples from different types (e.g., feces and gastric mucosa) also negatively impact the model’s predictive ability, as seen in the models trained withthe Wang et al. [31] dataset.
- The “objective of the study” for new samples should be similar to that used for training. For example, the model generated from Di Paola et al. [29], which focused on children, could not accurately predict ages in other studies, indicating that the taxa associated with infant age differ from those associated with adult age, supporting the idea discussed in [23].
- To build a robust model, the metadata must be complete and “clean,” just like in any machine learning process. For instance, samples with sick children should not be included in the metadata if the researcher wants to build a model that links health taxa to infant age, since the model might lean on this bias from these sick samples. However, if the model were properly constructed, it might be possible to use it to identify sick samples, since if the new sample’s prediction deviates significantly from the actual value (supposing you know the real value), it may indicate the existence of a disease.
- Regarding the adequacy of the training datasets, the metrics offered by CODARFE can be used to determine whether the present sample size is inadequate. For instance, if the hold-out test’s *R*² is insufficient (~<0.4) or the *P* value is not significant (≥0.05), the user will be aware that the samples are insufficient and will have the freedom to decide whether or not to utilize the model. All these metrics and functions are built in CODARFE class.
- The current version of CODARFE only performs regression for a single variable, which means that it does not control the effects of multiple variables. If the researcher is interested in the association between the microbiome and a numerical variable while ignoring the effects of a confounder variable, one way to do this would be to apply CODARFE to the residuals of a regression between the 2 variables. This logic even allows CODARFE to be expanded to other types of variables. Applying a logistic transformation on relative data, for instance, can result in a logistic CODARFE.
- CODARFE can be applicable to data other than taxonomic units, such as protein functions and metabolic pathways, since the mathematical constraints are the same for all of them. As proof of concept, a 10-repetition train–test split on 2 different projects collected from MGnify linking GO and InterPRO terms to water temperature are made available in Supplementary Material 3 (Supplementary Fig. S3.1 and S3.2).

## Conclusion

We present CODARFE, a powerful tool capable of selecting sparse compositional predictors and associating them with continuous environmental variables, which in turn can be used to predict these variables in new samples. CODARFE’s performance was benchmarked against 4 other tools and exhibited superior results in 21 of 24 tested datasets, with over 0.1 units stronger in terms of *R*^2^ on average and 17 times faster than the most recent published tools, particularly when the number of predictors surpassed 1,500. For human-related data, CODARFE achieved the highest number of identified taxa linked to the target variable that were also identified in the publication describing the study. Furthermore, we demonstrated CODARFE’s predictive power in cross-study analyses, where a model trained on data from 1 project could be successfully used to predict data from another project, with a maximum error rate of 18% in relation to the target variable mean, as long as the sequence region and sample type are the same. These results indicate CODARFE’s potential for generalization and robustness across multiple studies, even under different experimental settings. Furthermore, CODARFE’s uniqueness lies in its ability to predict the target variable in new samples, due to its imputation method for missing predictors. This feature allows for predicting environmental variables in previously unexplored contexts, providing researchers with a reliable and versatile tool.

## Availability of Source Code and Requirements

- Project name: CODARFE
- Project homepage: https://github.com/alerpaschoal/CODARFE
- Operating system(s): Platform independent (except for the graphical user interface, which was developed specifically for Windows)
- Programming language: Python
- Other requirements: Python 3.10 or higher
- License: Apache License Version 2.0
- SciCrunch.org ID: SCR_026180
- biotools ID: biotools:codarfe
- Software Heritage Library with the PID: swh:1:snp:cf926f8bf9f8ab5b14d9a2fb174d6b2d445ac425 [58]

## Supplementary Material

giaf055_Supplemental_File

giaf055_Authors_Response_To_Reviewer_Comments_original_submission

giaf055_Authors_Response_To_Reviewer_Comments_Revision_1

giaf055_GIGA-D-24-00556_original_submission

giaf055_GIGA-D-24-00556_Revision_1

giaf055_GIGA-D-24-00556_Revision_2

giaf055_Reviewer_1_Report_Original_submission Jaak Truu -- 1/30/2025

giaf055_Reviewer_1_Report_Revision_1 Jaak Truu -- 3/24/2025

giaf055_Reviewer_2_Report_Original_submission Reviewer 2 -- 2/4/2025

giaf055_Reviewer_2_Report_Revision_1 Reviewer 2 -- 3/28/2025

## Data Availability

The CODARFE plots and analysis are hosted in Zenodo [60], and data and code for CODARFE analysis are also hosted in Zenodo [61]. The original raw data of group A can be found in [59]; the Group B can be found in GitHub: infant age [62], PCDAI using baseline CD ileum [63], PCDAI using baseline CD rectum [64], Nugent score [65], and vaginal pH [66]. Group C is from the MGnify Projects [67], with accession numbers MGYS00000916, MGYS00001160, MGYS00000580, MGYS00001175, MGYS00001188, and MGYS00001255. The machine learning annotations have been deposited in the DOME registry [68].

## Additional Files

**Supplementary Fig. S1**. This flowchart shows CODARFE’s data acquisition and usage. Group A: Literature comprises 19 soil health metrics extracted from the article [Wilhelm et al. [59]]. Group B: Machine learning repository [Vangay et al. [22]], composed of 5 human disease indexes and linked to articles explaining taxa association. Group C: The MGnify database [Richardson et al. [26]] comprises 2 sets: 1 of 2 soil datasets and 4 human health measures. Each group was used for 1 or more analyses, as depicted by the image.

**Supplementary Fig. S2**. Complete distribution of all 19 soil health metrics across all tools. The y-axis represents the real value, while the x-axis represents the normalized (values between 0-1) prediction of each tool.

**Supplementary Fig. S3**. Distribution of the best, median and worst case of the hold-out validation process. The best case (surface_hardness) presents the highest amplitude, while the worst case (respiration) presents the lowest amplitude.

**Supplementary Table. S1**. Number of samples and columns (taxa) for each soil health metric present in “Group A: Literature”.

**Supplementary Table S2**. Number of samples and columns (taxa) for each human health metric present in “Group B: ML Repo”.

**Supplementary Table S3**. Number of samples and columns (taxa) for each soil health metric present in “Group C: cross studies”. The first four are related to human studies, and the last two are regarding soil health.

**Supplementary Fig. S3.1**. The *R*² of 10 repetitions train-test split for the GO and IPR terms for project MGYS00002008. The IPR explains the water temperature significantly better than the GO terms.

**Supplementary Fig. S3.2**. The *R*² of 10 repetitions train-test split for the GO and IPR terms for project MGYS00006074. There is no significant difference between the explainability of temperature in GO and IPR terms.

## Abbreviations

CLR: centered log ratio; CODA: compositional data analysis; CV: coefficient of variation; ILR: isometric log ratio; MAE: mean absolute error; MAPE: mean absolute percentage error; MHT: model hyperparameter transformation; ML: machine learning; PCDAI: Paediatric Crohn’s Disease Activity Index; RF: random forest; RFE: recursive feature elimination; RMSE: rooted mean squared error.
