# Supplementary material for: CODARFE: Unlocking the prediction of continuous environmental variables based on microbiome
Source: Gigascience. 2025 Jun 23;14:giaf055. doi: 10.1093/gigascience/giaf055 (PMC12365963; doi:10.1093/gigascience/giaf055)
Supplement: giaf055_Supplemental_File [file giaf055_supplemental_file.zip › SUPPLEMENTARY MATERIAL 1 .docx]

SUPPLEMENTARY MATERIAL


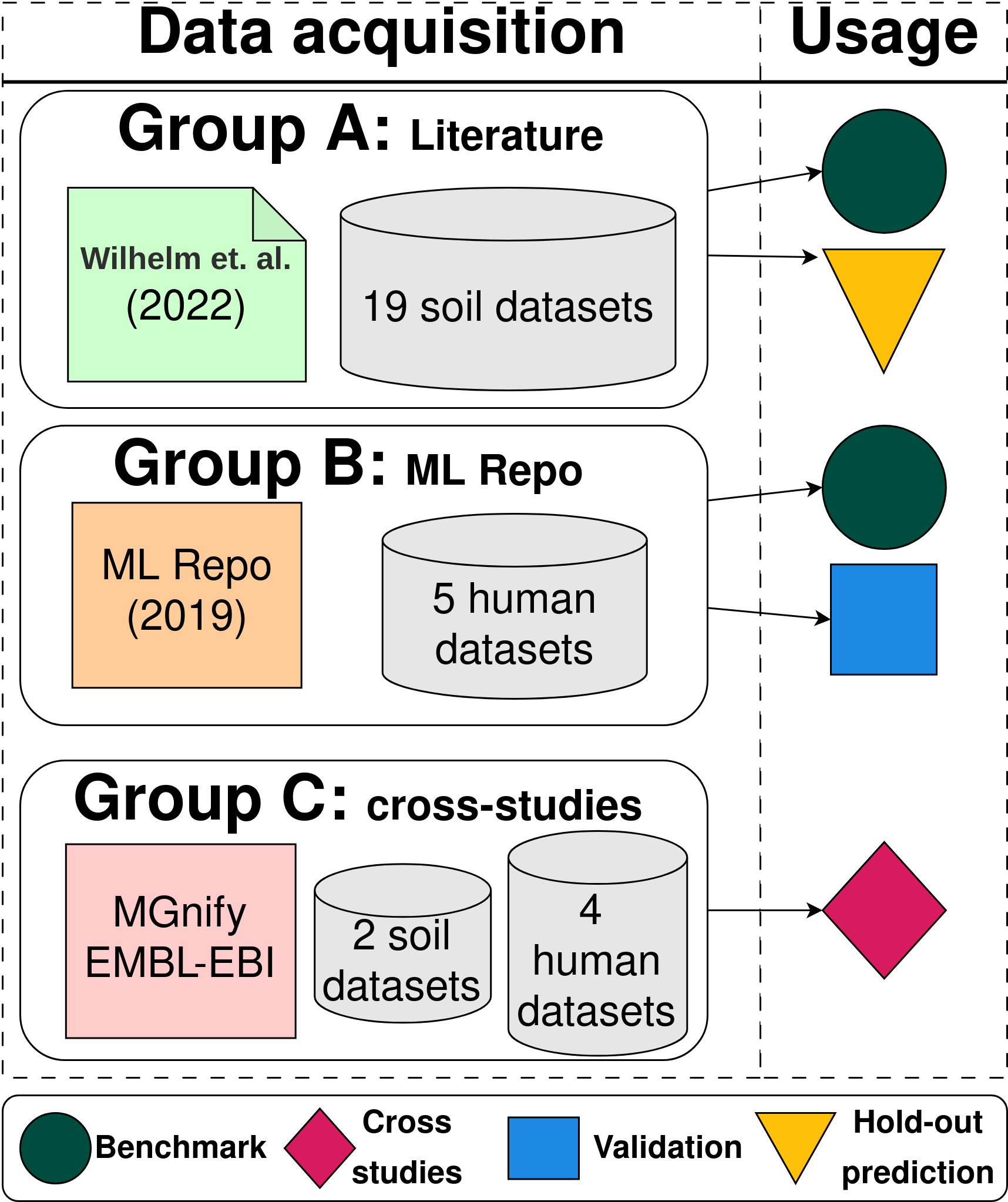

**Fig. S1. This flowchart shows the CODARFE’s data acquisition and usage. Group A: Literature comprises 19 soil health metrics extracted from the article [Wilhelm et al., 2022]. Group B: Machine Learning repository [Vangay et al., 2019], composed of five human disease indexes and linked to articles explaining taxa association. Group C: The MGnify database [Richardson et al., 2023] comprises two sets: one of two soil datasets and four human health measures. Each group was used for one or more analyses, as depicted by the image.**


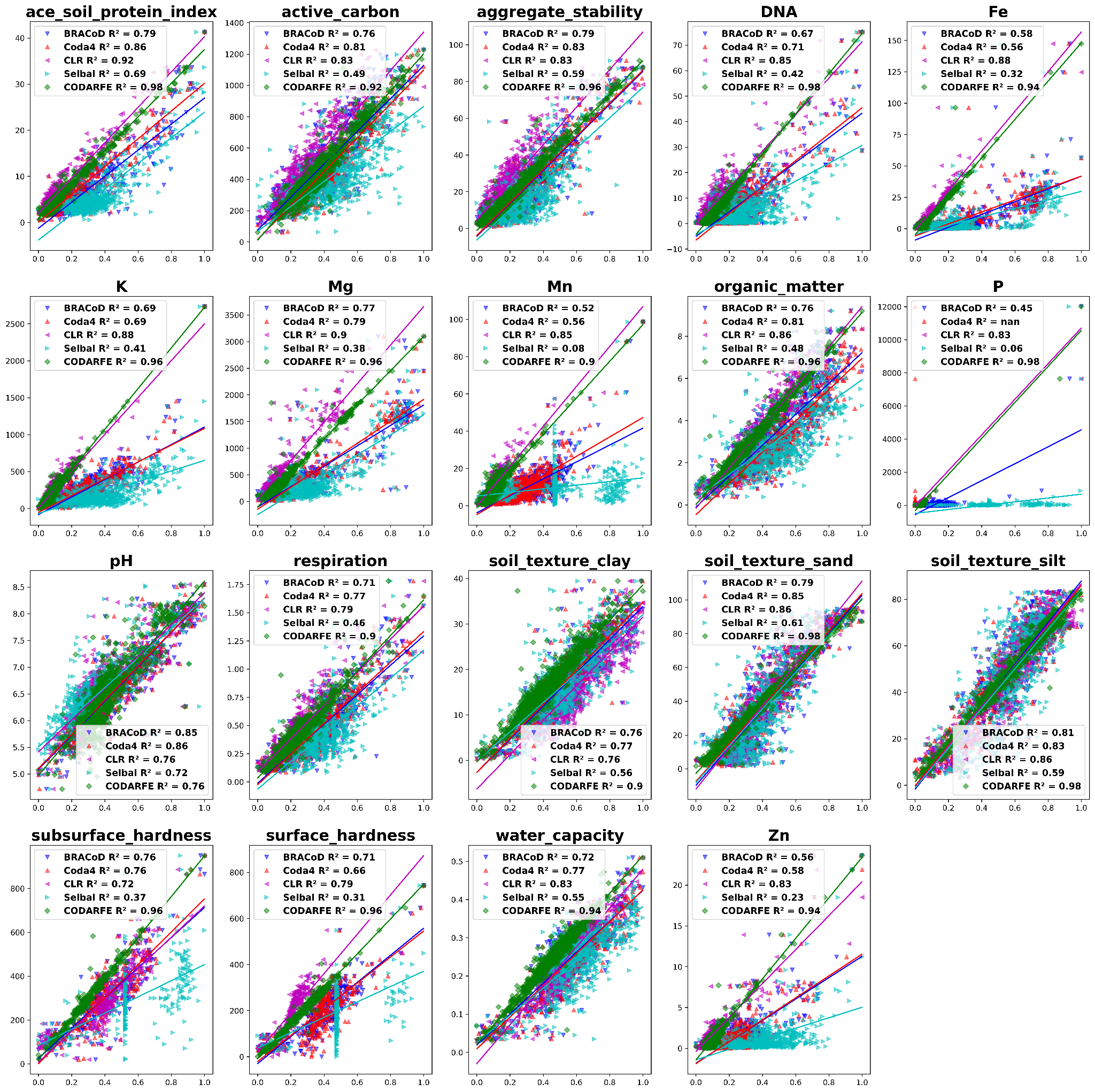

**Fig. S2. Complete distribution of all 19 soil health metrics across all tools. The y-axis represents the real value, while the x-axis represents the normalized (values between 0-1) prediction of each tool.**


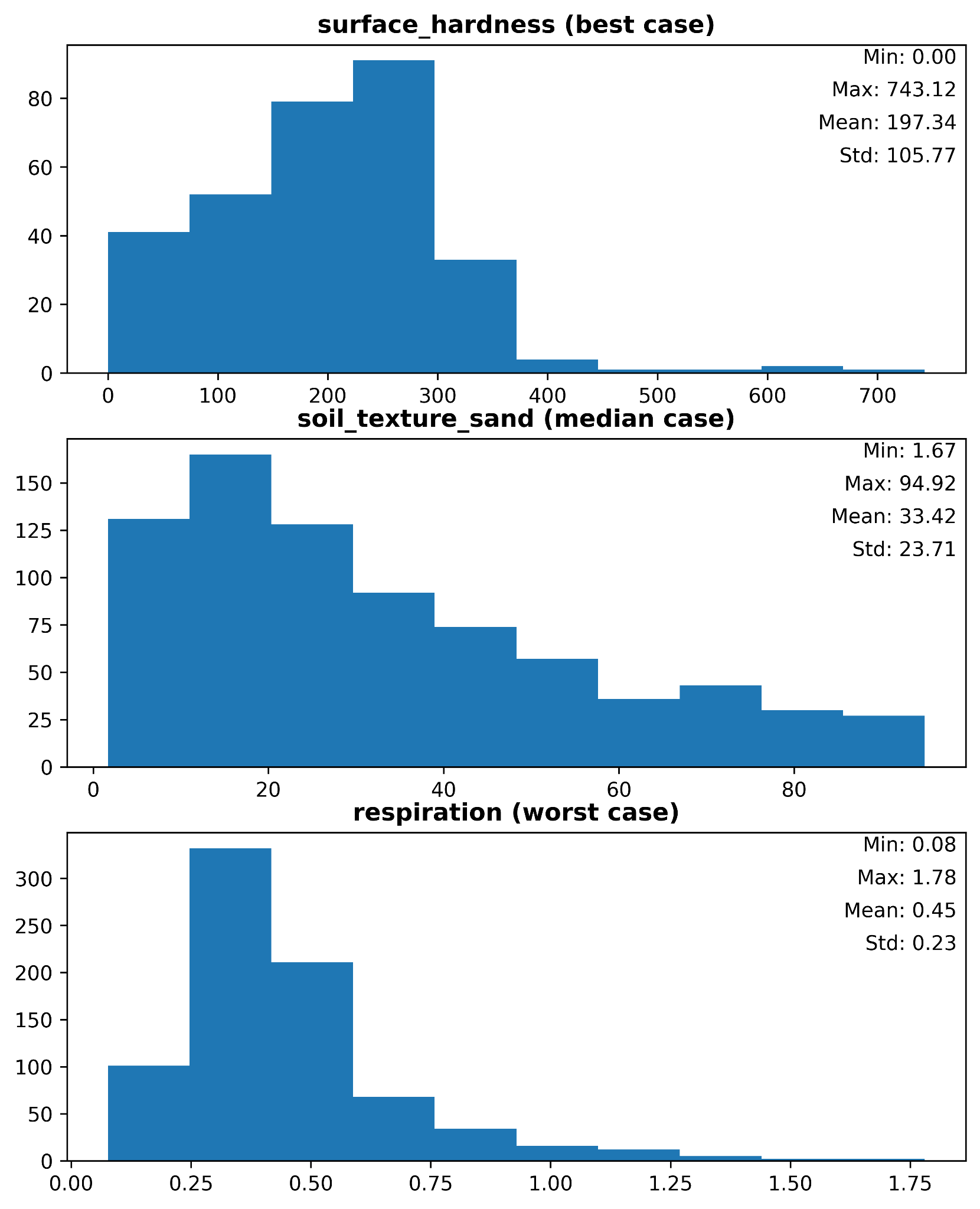


**Fig. S3. Distribution of the best, median and worst case of the hold-out validation process. The best case (surface_hardness) presents the highest amplitude, while the worst case (respiration) presents the lowest amplitude.**

| **Target name** | **Number of samples** | **Number of columns** |
| --- | --- | --- |
| aggregate_stability | 783 | 94014 |
| P | 783 | 94014 |
| K | 783 | 94014 |
| pH | 783 | 94014 |
| subsurface_hardness | 288 | 94014 |
| surface_hardness | 305 | 94014 |
| water_capacity | 780 | 94014 |
| ace_soil_protein_index | 783 | 94014 |
| DNA | 783 | 94014 |
| Fe | 783 | 94014 |
| Mn | 783 | 94014 |
| organic_matter | 783 | 94014 |
| respiration | 783 | 94014 |
| Zn | 783 | 94014 |
| Mg | 783 | 94014 |
| active_carbon | 783 | 94014 |
| soil_texture_sand | 783 | 94014 |
| soil_texture_silt | 783 | 94014 |
| soil_texture_clay | 783 | 94014 |

**Table S1. Number of samples and columns (taxa) for each soil health metric present in “Group A: Literature”.**

| **Target name** | **Number of samples** | **Number of columns** |
| --- | --- | --- |
| ileum | 68 | 944 |
| infant_age | 49 | 4661 |
| nugent | 388 | 587 |
| pH | 388 | 587 |
| rectum | 51 | 944 |

**Table S2. Number of samples and columns (taxa) for each human health metric present in “Group B: ML Repo”.**

| **Target name** | **Number of samples** | **Number of columns** |
| --- | --- | --- |
| MGYS00001175 | 50 | 521 |
| MGYS00001188 | 100 | 1928 |
| MGYS00001255 | 325 | 1243 |
| MGYS00000580 | 50 | 357 |
| MGYS00000916 | 25 | 522 |
| MGYS00001160 | 25 | 607 |

**Table S3. Number of samples and columns (taxa) for each soil health metric present in “Group C: cross studies”. The first four are related to human studies, and the last two are regarding soil health.**
