# Supplementary material for: CODARFE: Unlocking the prediction of continuous environmental variables based on microbiome
Source: Gigascience. 2025 Jun 23;14:giaf055. doi: 10.1093/gigascience/giaf055 (PMC12365963; doi:10.1093/gigascience/giaf055)

**The taxa that were found to be associated with the interest variable for each of the three publications (Group B: ML Repo) that were utilized to confirm the relevance of the taxa chosen by the various methods are described here.**

**1) Yatsunenko et al. (2012):** *Bifidobacteruim* was pointed out by the authors as a potential biomarker for infant age.

Selected by each tool: Only CODARFE and BRACoD selected *Bifidobacterium* species

**
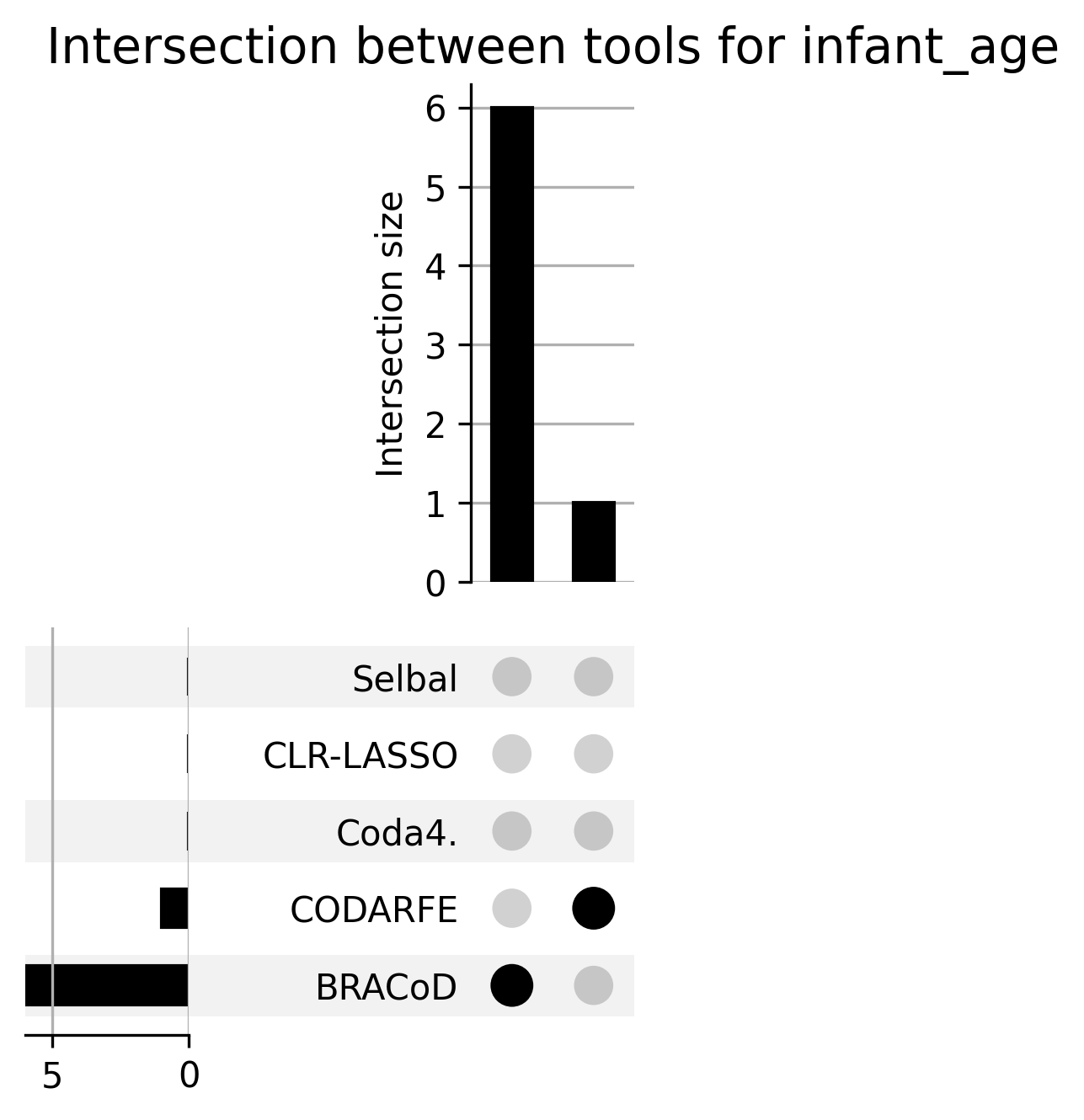


2) Ravel et al. (2010):** The following phylotypes were associate with high Nugent scores: *Aerococcus, Anaeroglobus, Anaerotruncus, Atopobium, Coriobacteriaceae 2, Dialister, Eggerthella, Gardnerella, Gemella, Megasphaera, Mo- biluncus, Parvimonas, Peptoiphilus, Prevotella, Porphyomonas, Prevotellaceae1, Prevotellaceae 2, Ruminococcaceae*, and *Snethia*. Conversely, phylotypes of *Lactobacillus* were associated with a low Nugent.

Selected by each tool: BRACoD correctly selected a total of 8 different phylotypes being them: *Anaeroglobus, Prevotella , Lactobacillus , Gemella , Atopobium , Aerococcus , Gardnerella,* and *Dialister*; Coda4Microbiome correctly selected 8 phylotypes being them: *Mobiluncus, Prevotella, Lactobacillus, Gemella, Atopobium, Aerococcus, Gardnerella,* and *Dialister*; CODARFE correctly selected 5 phylotypes being them: *Prevotella, Lactobacillus, Atopobium, Gardnerella,* and *Dialister*; CLR-LASSO correctly selected a total of 4 phylotypes being them: *Prevotella, Lactobacillus, Gemella,* and *Atopobium*; and Selbal selected correctly 5 phylotypes being them: *Prevotella, Lactobacillus, Atopobium, Gardnerella,* and *Dialister*.


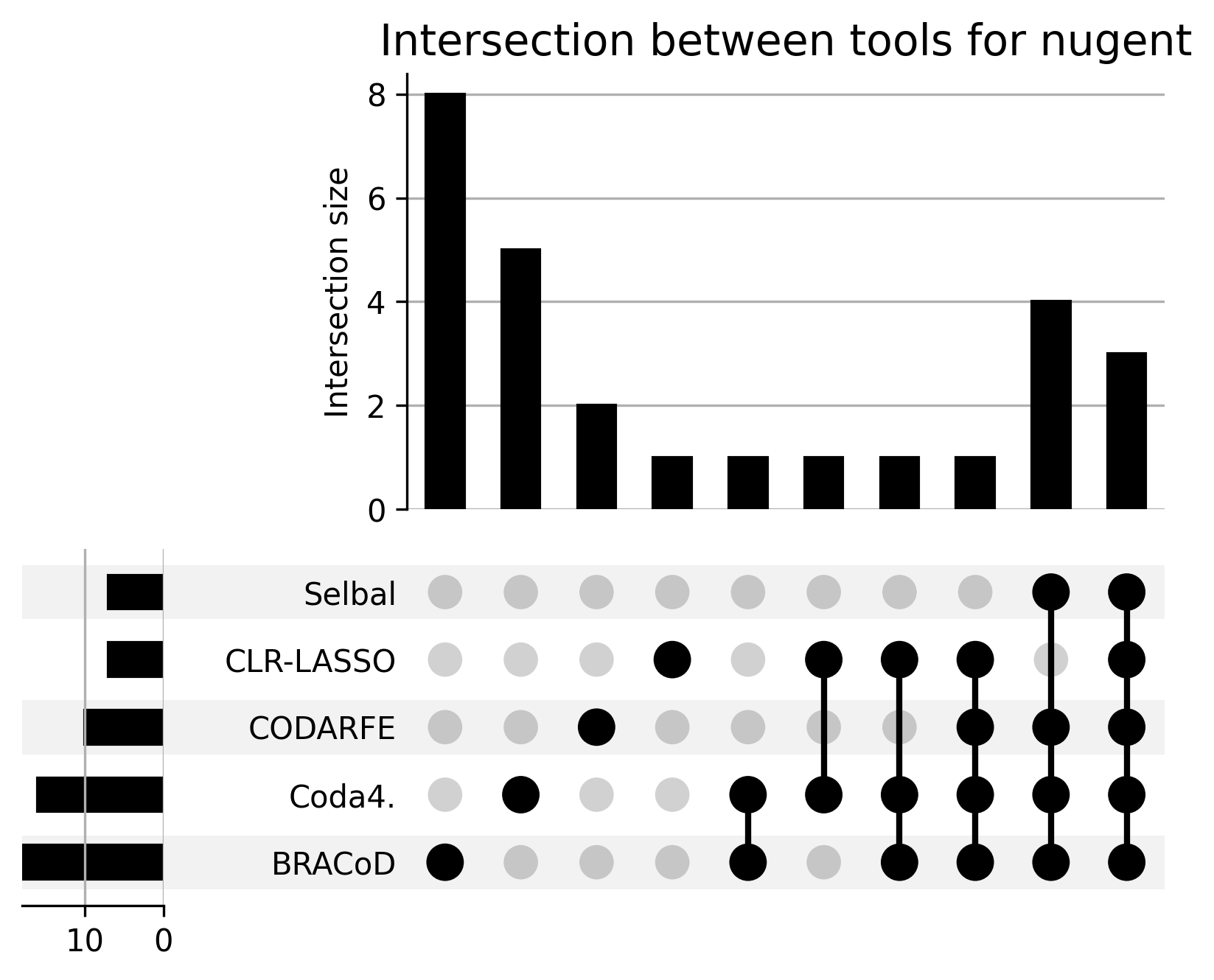


For vaginal pH, only the presence or absence of *Lactobacillus* was reported to be relevant, with its presence being inversely proportional to the pH measurement.


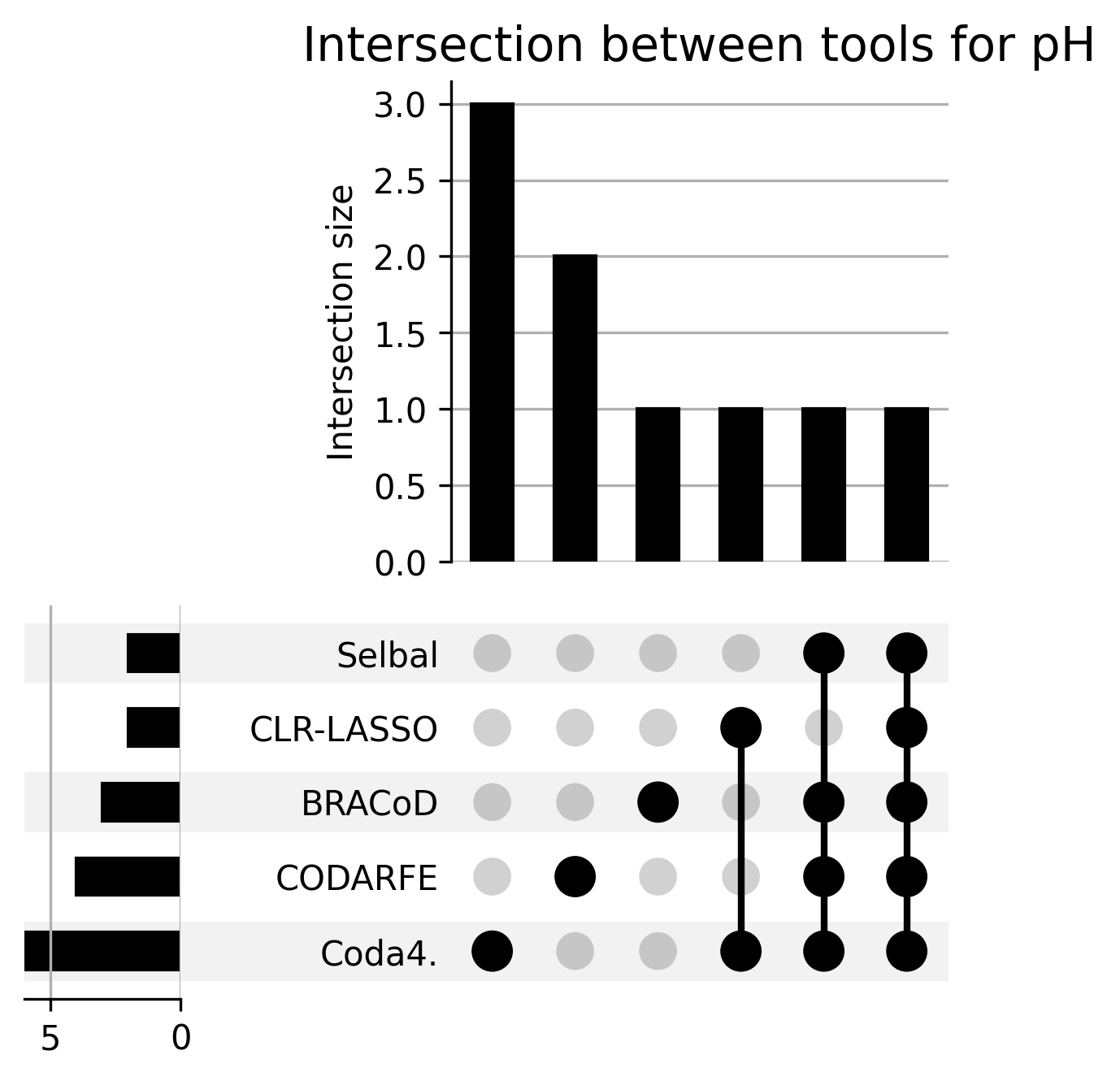


**3) Gevers et al. (2014):** The authors identified 21 species with a high correlation to PCDAI: *Escherichia coli, Fusobacterium nucleatum, Haemophilus parainfluenzae, Veillonella parvula, Eikenella corrodens, Gemella moribillum, Bacteroides vulgatus, Bacteroides caccae, Bifidobacterium bifidum, Bifidobacterium longum, Bifidobacterium ado- lescentis, Bifidobacterium dentum, Blautia hansenii, Ruminococ- cus gnavus, Clostridium nexile, Faecalibacterium prausnitzii, Ruminoccus torques, Clostridium bolteae, Eubacterium rectale, Roseburia intestinalis,* and *Coprococcus comes*.

Selected by each tool: BRACoD correctly selected *Haemophilus parainfluenzae, Veillonella parvula,* and *Bacteroides vulgatus* for the Ileum dataset and *Fusobacterium nucleatum Eikenella corrodens, Bifidobacterium bifidum,* and *Bifidobacterium longum* for rectum dataset; As previously described, Coda4Microbiome was unable to finish its process for the Ileum dataset, and did not select any of the 21 species pointed out by the article in the rectum dataset; CODARFE correctly selected *Fusobacterium nucleatum, Bacteroides vulgatus, Faecalibacterium prausnitzii,* and *Roseburia intestinalis* for ileum dataset and *Haemophilus parainfluenzae, Bacteroides vulgatus, Bifidobacterium longum,* and *Eubacterium rectale* for rectum dataset; CLR-LASSO selected only *Veillonella parvula* for ileum and rectum; and Selbal did not select any of the 21 species


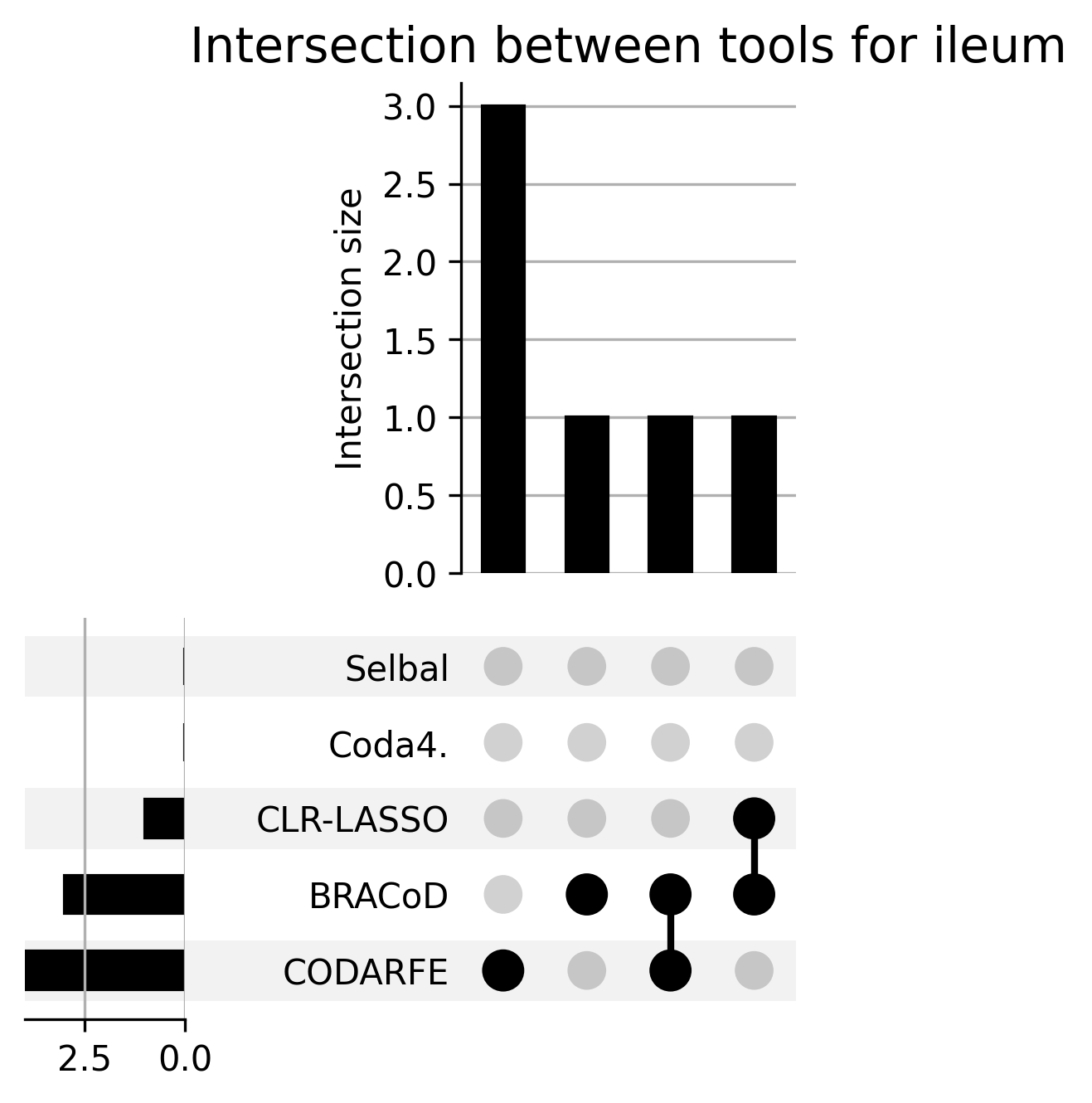

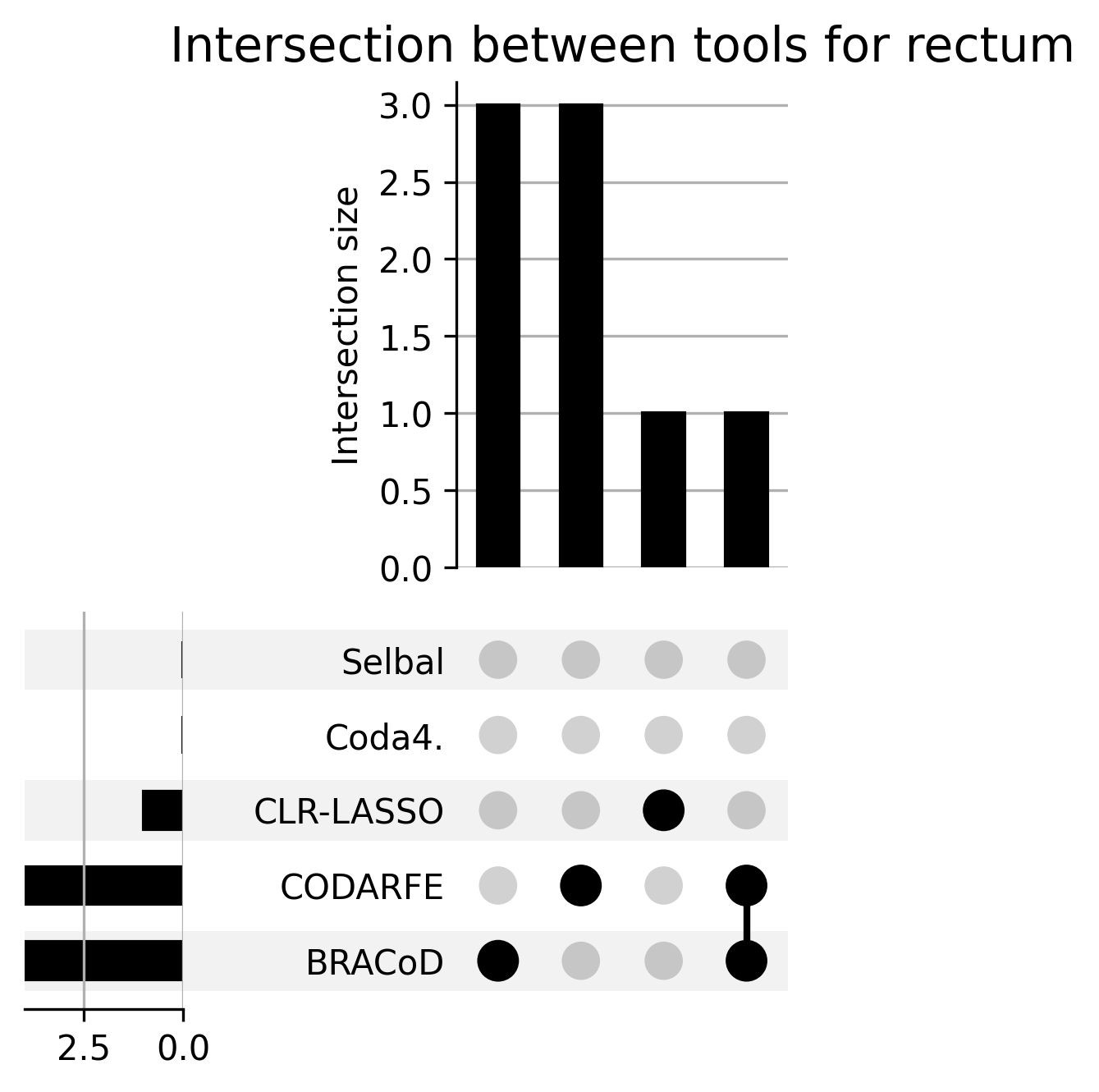

Supplement: giaf055_Supplemental_File [file giaf055_supplemental_file.zip › SUPPLEMENTARY MATERIAL 2.docx]
