# Supplementary material for: CODARFE: Unlocking the prediction of continuous environmental variables based on microbiome
Source: Gigascience. 2025 Jun 23;14:giaf055. doi: 10.1093/gigascience/giaf055 (PMC12365963; doi:10.1093/gigascience/giaf055)
Supplement: giaf055_Supplemental_File [file giaf055_supplemental_file.zip › SUPPLEMENTARY MATERIAL 3.docx]

**Here, we demonstrate the feasibility of using CODARFE for data formats other than taxonomic units.**

To accomplish this, we randomly select two MGnify projects that have a continuous environmental element to use as targets. Projects MGYS00002008 and MGYS00006074, which deal with aquatic studies, were chosen since the metadata included the water temperature that could be used as a target.

Both projects contain the GO (Gene Ontology - <https://doi.org/10.1093/bib/6.3.298>) and InterPRO (called just IPR - <https://doi.org/10.1093/nar/gkac993>) annotations in the format of “feature count”, similar to the taxonomic units table used in this work. The same metadata is associated with each of these terms (GO and IPR). In this manner, it is possible to compare the predictive power of each term in relation to the water temperature. The figures S3.1 and S3.2 illustrate the results of a 10 repetitions train-test split for each term in terms of R².


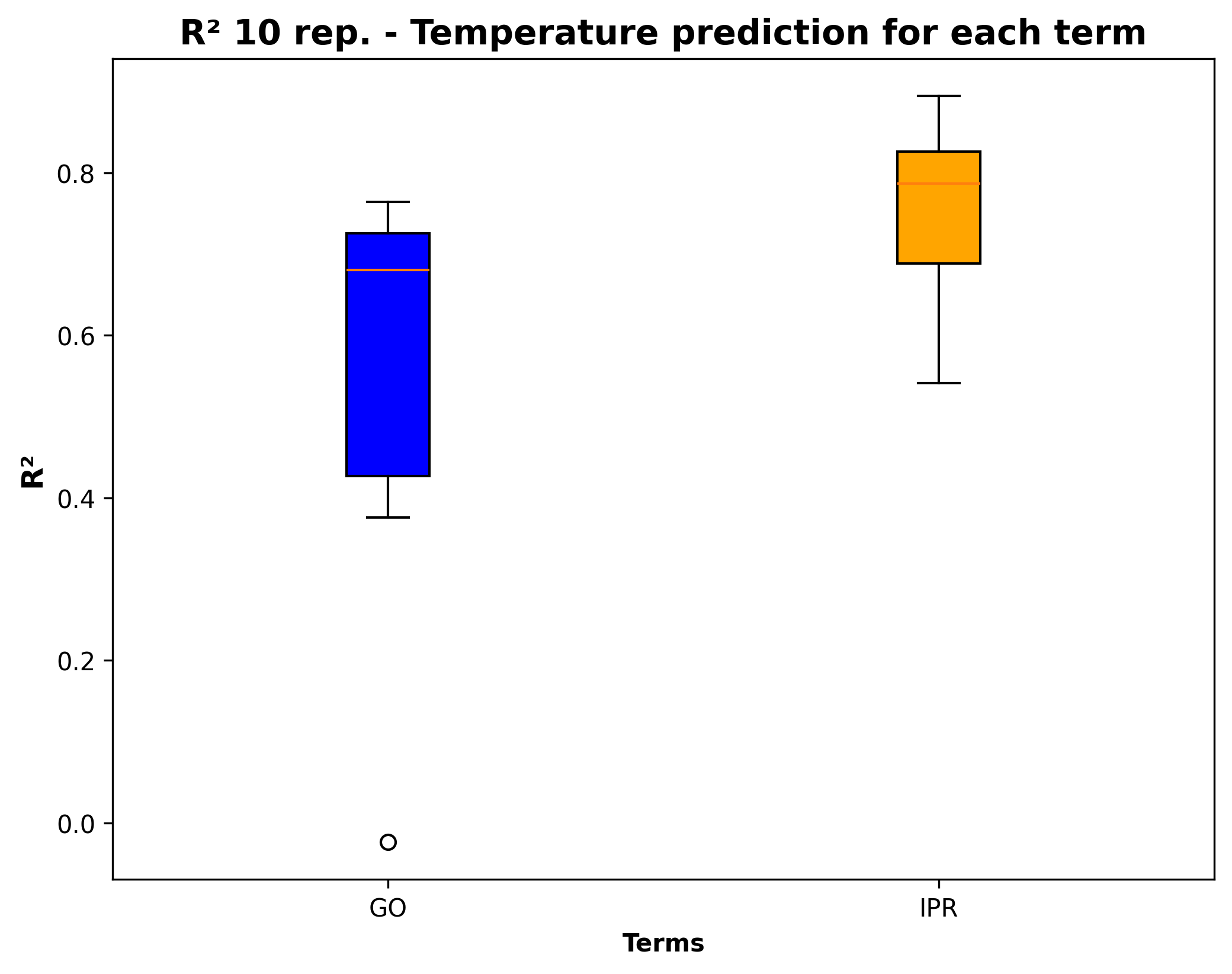


**Figure S3.1) The R² of 10 repetitions train-test split for the GO and IPR terms for project MGYS00002008. It is possible to note that the IPR explains the water temperature significantly better than the GO terms.**


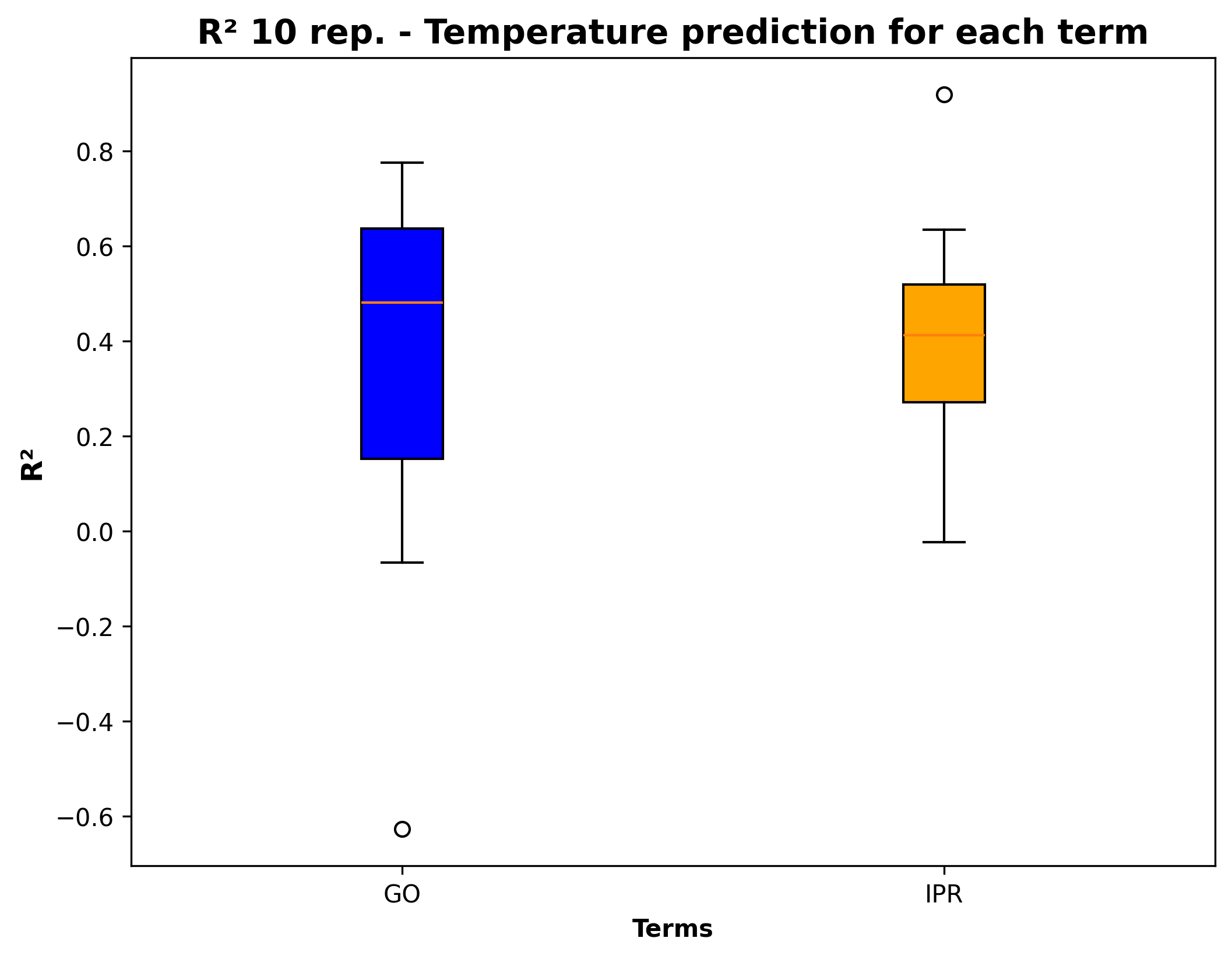


**Figure S3.2) The R² of 10 repetitions train-test split for the GO and IPR terms for project MGYS00006074. It is possible to note that there is no significant difference between the explainability of temperature in GO and IPR terms.**

All the datasets and codes used to generate these images are publicly available at: [**https://doi.org/10.5281/zenodo.14278004**](https://doi.org/10.5281/zenodo.14278004) .
