# Supplementary material for: CODARFE: Unlocking the prediction of continuous environmental variables based on microbiome
Source: Gigascience. 2025 Jun 23;14:giaf055. doi: 10.1093/gigascience/giaf055 (PMC12365963; doi:10.1093/gigascience/giaf055)
Supplement: giaf055_GIGA-D-24-00556_original_submission [file giaf055_giga-d-24-00556_original_submission.pdf]

GigaScience

CODARFE: Unlocking the prediction of continuous environmental variables based on microbiome

--Manuscript Draft--

**Manuscript Number:**	GIGA-D-24-00556
**Full Title:**	CODARFE: Unlocking the prediction of continuous environmental variables based on microbiome
**Article Type:**	Research
**Funding Information:**	Fundação Araucária (#66.2021) — Dr Alexandre Rossi Paschoal<br>CNPq (#440412/2022-6) — Dr Alexandre Rossi Paschoal<br>European Bioinformatics Institute — Dr Robert D Finn<br>SUPERBAC Biotechnology Solutions (02,2021) — M.Sc Murilo Caminoto Barbosa<br>CAPES, PDSE (88881.846275/2023-01) — M.Sc Murilo Caminoto Barbosa

Abstract:

Background

Despite the surge in microbiome data acquisition, there is a limited availability of tools capable of effectively analyzing it and identifying correlations between taxonomic compositions and continuous environmental factors. Furthermore, existing tools also do not predict the environmental factors in new samples, underscoring the pressing need for innovative solutions to enhance our understanding of microbiome dynamics and fulfill the prediction gap. Here we introduce CODARFE, a novel tool for sparse compositional microbiome-predictors selection and prediction of continuous environmental factors.

Results

We tested CODARFE against four state-of-the-art tools in two experiments. First, CODARFE outperformed predictor selection in 21 out of 24 databases in terms of correlation. Second, among all the tools, CODARFE achieved the highest number of previously identified bacteria linked to environmental factors for human data—that is, at least 7% more. We also tested CODARFE in a cross-study, using the same biome but under different external effects, using a model trained on one dataset to predict environmental factors on another dataset, achieving 11% of mean absolute percentage error. Finally, CODARFE is available in five formats, including a Windows version with a graphical interface, to installable source code for Linux servers and an embedded Jupyter notebook available at MGnify.

Conclusions

Our findings underscore the robustness and broad applicability of CODARFE across diverse fields, even under varying experimental conditions. Additionally, the ability to predict outcomes in new samples allows for the generation of new insights in previously unexplored contexts, providing researchers with a versatile tool.

**Corresponding Author:**	Murilo Caminoto Barbosa, MD<br>UTFPR: Universidade Tecnologica Federal do Parana<br>Cornelio Procopio, Paraná BRAZIL
**Corresponding Author Secondary Information:**	
**Corresponding Author's Institution:**	UTFPR: Universidade Tecnologica Federal do Parana
**Corresponding Author's Secondary Institution:**	

Powered by Editorial Manager® and ProduXion Manager® from Aries Systems Corporation

First Author:	Murilo Caminoto Barbosa, MD
First Author Secondary Information:	
Order of Authors:	Murilo Caminoto Barbosa, MD
	João Fernando Marques da Silva
	Leonardo Cardoso Alves
	Robert D Finn
	Alexandre Rossi Paschoal
Order of Authors Secondary Information:	
Additional Information:	
**Question**	**Response**
Are you submitting this manuscript to a special series or article collection?	No
**Experimental design and statistics**<br><br>Full details of the experimental design and statistical methods used should be given in the Methods section, as detailed in our Minimum Standards Reporting Checklist. Information essential to interpreting the data presented should be made available in the figure legends.<br><br>Have you included all the information requested in your manuscript?	Yes
**Resources**<br><br>A description of all resources used, including antibodies, cell lines, animals and software tools, with enough information to allow them to be uniquely identified, should be included in the Methods section. Authors are strongly encouraged to cite Research Resource Identifiers (RRIDs) for antibodies, model organisms and tools, where possible.<br><br>Have you included the information requested as detailed in our Minimum Standards Reporting Checklist?	Yes
**Availability of data and materials**	Yes

All datasets and code on which the conclusions of the paper rely must be either included in your submission or deposited in publicly available repositories (where available and ethically appropriate), referencing such data using a unique identifier in the references and in the "Availability of Data and Materials" section of your manuscript.<br><br>Have you have met the above requirement as detailed in our Minimum Standards Reporting Checklist?	
GigaScience has policies and guidelines in place for the use of generative AI-writing tools such as ChatGPT. If you have used such writing tools to assist with writing the manuscript this must be declared and cited in the text. Authors should not list AI-writing tools and other AI-assisted technologies as an author or co-author and should acknowledge that they are fully responsible for text generated or refined by AI-writing tools.<p><br><br>A summary of use (particularly in the introduction or among methods) needs to be included at the end of the paper, and the outputs should also be included as a supplementary file hosted in GigaDB or other open repositories. Please read our guidelines for more information. <p><br><br>By submitting to GigaScience, you are aware of the journal's AI-writing tools policy, and if you have declared use of such tools below, you have acknowledged this where appropriate in your manuscript and have made a summary of use and outputs available. <p><br><b>AI-assisted writing tools have been used in the preparation of this manuscript?	No

Powered by Editorial Manager® and ProduXion Manager® from Aries Systems Corporation

Click here to access/download;Manuscript;Manuscript - Reviewed.docx

CODARFE: Unlocking the prediction of continuous environmental variables based on microbiome

Murilo Caminotto Barbosa[1,*,†,‡,§,¶,**], João Fernando Marques da Silva[2,†,‡,¶,**], Leonardo Cardoso Alves[2,†,‡,¶,**], Robert D Finn[3,¶,**] and Alexandre Rossi Paschoal[1,4,*,†,‡,¶,**]

[1] Universidade Tecnológica Federal do Paraná, Avenida Alberto Carazzai, 1640, 86300-000, Paraná, Brazil

[2] Centro de Inovação, Superbac Biotechnology Solutions, Estr. Vitória de São Pedro 685, 86975-000, Mandaguari, Brazil

[3] European Molecular Biology Laboratory, European Bioinformatics Institute (EMBL-EBI), Wellcome Genome Campus, Hinxton, Cambridge, UK

[4] Rosalind Franklin Institute, Harwell Campus Road Thirteen, OX11 0QX, Didcot, UK

*murilobarbosa@alunos.utfpr.edu.br; paschoal@utfpr.edu.br

\†Conceptualization
‡Methodology
§Software development
¶Validation
**Revision and Writing

Abstract

Background

Despite the surge in microbiome data acquisition, there is a limited availability of tools capable of effectively analyzing it and identifying correlations between taxonomic compositions and continuous environmental factors. Furthermore, existing tools also do not predict the environmental factors in new samples, underscoring the pressing need for innovative solutions to enhance our understanding of microbiome dynamics and fulfill the prediction gap. Here we introduce CODARFE, a novel tool for sparse compositional microbiome-predictors selection and prediction of continuous environmental factors.

Results

We tested CODARFE against four state-of-the-art tools in two experiments. First, CODARFE outperformed predictor selection in 21 out of 24 databases in terms of correlation. Second, among all the tools, CODARFE achieved the highest number of previously identified bacteria linked to environmental factors for human data—that is, at least 7% more. We also tested CODARFE in a cross-study, using the same biome but under different external effects, using a model trained on one dataset to predict environmental factors on another dataset, achieving 11% of mean absolute percentage error. Finally, CODARFE is available in five formats, including a Windows version with a graphical interface, to installable source code for Linux servers and an embedded Jupyter notebook available at MGnify.

Conclusions

Our findings underscore the robustness and broad applicability of CODARFE across diverse fields, even under varying experimental conditions. Additionally, the ability to predict outcomes in new samples allows for the generation of new insights in previously unexplored contexts, providing researchers with a versatile tool.

Key words: Compositional-Data-Analysis; Microbiome; Prediction; Feature Selection; Machine Learning

Key Points

- CODARFE is a novel tool designed to select sparse compositional microbiome predictors and predict continuous environmental factors, filling a critical gap in microbiome analysis
- Its predictive power extends across different studies and conditions, achieving 11% mean absolute percentage error in cross-study predictions, showing generalization potential even with varying external effects.
- In testing against four state-of-the-art tools, CODARFE outperformed 21 out of 24 datasets, showing stronger correlation and significantly faster performance.
- CODARFE is versatile, available in multiple formats, including a graphical interface for Windows and a Jupyter notebook, and has potential for use with data types other than 16s.

Background

Microbiomes encompass a vast array of microorganisms, including bacteria, viruses, fungi, and protozoa [1], inhabiting diverse environments including the human body, plants, soils, and even the International Space Station [2]. This microbial diversity has become a focal point of research to understand the role of microbes in health and disease [3, 4, 5]. Consequently, this has led to significant interest in identifying specific microbial biomarkers that can serve as indicators for these conditions, providing valuable insights into both the diseases themselves and the environmental factors associated with them [6, 7]. However, comparison between microbiome data coming from two or more samples can present specific challenges owing to the composition variability, hence the need to develop appropriate statistical approaches [8, 9].

Analyzing microbial communities typically means measuring the proportions of different microbial taxa present in a sample, however this information is conveyed as ratios or percentages relative to the total microbial community in that sample, creating dependencies among the species proportions [10]. Additionally, data sparsity arises from the presence of rare or low-abundance species that are difficult to detect and quantify [8, 11].

Given these complexities, compositional data analysis (CoDA) techniques have emerged as a prominent research focus in the field of microbiome research [12]. With its

help, the statistical analysis then can be used to associate the microbiome predictors – in this research being exclusively taxa – to any target variable [13]. In the context of this research, the “target variable” can be any continuous environmental or clinical variable related to the microbiome.

Although machine learning (ML) techniques have shown promise [14,15], and their application to microbiome data analysis tools is expanding [16, 17, 18, 19, 20], a basic need still exists: the ability to predict the variable of interest in new samples [21]. This challenge arises from the improbability of two samples collected in different times - even if they are collected in very close areas - to have the same set of bacterias. To address this challenge, we present CODARFE (COmpositional Data Analysis with Recursive Feature Elimination), a new tool that combines CoDA methods with a Recursive Feature Elimination (RFE) process and ML. This approach enables the association of microbiome compositional data with a continuous environmental variable. It also integrates a module for missing predictor imputation and enables the prediction of target environmental variables in new samples.

Material and Methods

Evaluation datasets

In this study we collected a total of 30 datasets from three data-sources, and for each source a set of analyses were conducted (Figure S1.1). The called “Group A: Literature” was collected with the aim of evaluating the coefficient of correlation and processing time required by different methods, and for a hold-out prediction evaluation. In total there are 19 datasets, one for each environmental variable [21].The “Group B: ML Repo” was collected to serve as a positive control. This is due to pre-existing evidence of a relationship between one or more taxa and the sample metadata variable of interest in the original publication associated with the dataset. The validation is achieved by calculating the number of taxa selected by the tools that are also supported by articles as linked to the target variable. It is composed of five human gut datasets obtained from the collection available in the “ML Repo” [22, 23, 24, 25], where two of these datasets pertain to pediatrics’s Crohn disease,

with samples collected from the rectum (n=51) and ileum (n=67); one from a study on the infant microbiome (age 0-2 years) (n=49); and two from the study on the vaginal health, which captured pH (n=388) and Nugent scores (n=388). The “Group C: cross studies” was collected with the purpose to evaluate CODARFE‘s generalization power and potential failings when training a model on samples from one project and predicting the variable of interest on a different project (from the same biome). Group C consists of two sets of datasets extracted from MGnify [26]. The first set comprises two projects with pH measurements in arable soil under the effect of ginseng field and cattle (Table 2) [27, 28] (MGnify identifiers: MGYS00000916 and MGYS00001160). The second set involves age measurements in humans from four projects with samples collected from different parts of the digestive system and subjected to various external effects (such as: antibiotic treatment, HIV, etc) (Table 3) [29, 30, 31, 32] (MGnify identifiers: MGYS00000580, MGYS00001175, MGYS00001188, MGYS00001255).

Data Preprocessing

The data preprocessing consists of two steps. In the first step, predictors whose variance across samples approximate to zero are removed. A value is considered close to zero if it is less than or equal to ⅛ of the mean variance found in the dataset, by this manner, removing columns that are considered poor in information [33].

The second step concerns only the target variable, where values are squared rooted and rearranged between 0-100 if its coefficient of variation (CV) is greater than or equal to 0.2, indicating a wide variation, which may suggest the presence of noise [34]. The square root serves to mitigate this data dispersion if required, which has been proven effective for reducing errors in machine learning models [35]. Additionally, if the CV is less than 0.2 and the target contains negative numbers, a simple shift is applied to allow the model to predict the target (due to the poisson distribution used in the model, described ahead).

Selection of CODA transformation and regression method

Subsequently, four machine learning algorithms were evaluated, using two distinct compositional data transformations, namely Hellinger and Center Log Ratio (CLR) [8]. These transformations were chosen due to their ability to mitigate the inherent issues of compositional data, where the Hellinger transformation is effective for preserving the Euclidean structure of the data [8], and the CLR transformation for its ability to transform compositional data into a real-valued space where traditional statistical methods can be applied without the risk of generating misleading results [13].

The algorithms evaluated were Linear Support Vector Regression [36], Linear Stochastic Gradient Descent[36], Huber regression [37], and Theil Sen estimator [38], collectively chosen for their ability to return weights corresponding to the importance of each predictor. Each algorithm was tested with a wide range of hyper- parameters and applied to each transformation, termed model-hyperparameter-transformation (MHT), resulting in 2270 combinations. Each MHT was evaluated using RFE [39], which removes 1% of the total predictors (columns) with the smallest weights at each iteration, generating up to a hundred trained models.

Four criteria were used to evaluate each MHT: the R^2 adjusted was chosen to determine the correlation strength with the target variable while selecting a concise number of predictors due to its penalization effect, collaborating to reduce the type 1 error during the fit process; BIC was chosen to differentiate models with similar R^2 and with different predictor quantities, and it also helps keep low type 1 error due its penalization related to the number of predictors; Rooted Mean Squared Error (RMSE) was used in cross-validation to reduce overfitting, being this the most important metric since overfitting has the worst impact in a prediction model [40]; and lastly, the p-value of the F-test is used to ensure the statistical significance of the selected predictors. All metrics were normalized using the “minmax” method (equation 1) and summed to create the model’s score. The selected MHT is the one with the highest model score. Figure 1A illustrates the complete process.

In the end of the process, the Huber regression yielded the highest scores when paired with the Centered-Log-Ratio (CLR) transformation. The “epsilon” parameter,

determining outlier influence, was set to 2, and the “alpha” value, controlling regularization, was set at 0.0003. Unfortunately, the CLR creates an influence of the non-selected predictors into the selected one due to the geometric mean used in its formula. This problem is mitigated with the use of the RFE, where on each interaction, the original data is rescued and re-transformed using only the remaining predictors. Furthermore, the RFE helps with the issues of data dimensionality, which iteratively reduces the number of columns by filtering for importance.

$$minmax(data) = (data - min(data))/(max(data) - min(data)) \quad \textbf{(1)}$$

CODARFE - Selector

The predictor selector is the core of the CODARFE. The results of the selector are the predictors used for correlation to the target and the predictors for the prediction model. It starts with the preprocessing step, then the CLR transformation is applied to the data. The data goes under a Recursive-Feature-Elimination with the Huber regression as an algorithm chosen to give weights (coefficients) to each predictor. The predictors are evaluated following the same process described in the previous section (“Selection of CODA transformation and regression method”), to produce the “Predictors scores”. The set of predictors with the highest score is defined as the predictors most associated with the target variable. Figure 1B depicts the complete process.

Figure 1. Pipelines from CODARFE-selector and selection of CLR and Huber Regressor as CoDA transformation and regression method.(A) Firstly, CODARFE-selector selects the closely related variables to the target. The data is preprocessed to remove low variation variables, followed by the RFE, in which the trained model is evaluated using four metrics. Finally, the model with the highest “predictors score” (the sum of the “minmax” of the four metrics) is chosen, and the variables used to train the model are stated as the most associated with the target. (B) Subsequently, the selection of CoDA transformation and regression method is performed. The data is preprocessed to remove low variance variables, then RFE is used to train the MHT and evaluate it using four metrics. Finally, the MHT with the highest score (the sum of the “minmax” of the four metrics) is selected.

Selection of predictor algorithm

For the predictor algorithm, a new set of regressors were test, namely: Linear Support Vector Regression [36], Linear Stochastic Gradient Descent[36], Huber regression

[37], Theil-Sen estimator [38], Random-Forest [41], and Support Vector Machine [36] with various kernels, resulting in 1243 combinations (Figure 2A). Only the CLR transformation was used. Each model was trained on 80% of the dataset and evaluated on 20% with 10 repetitions to calculate the average of the Mean Absolute Error (MAE) estimations (Figure 2A). The Random Forest with parameters "n_estimators = 160" and "criterion = poisson" resulted in a better performance for predictions and was chosen for use in COADRFE.

CODARFE - Predictor Model

The CODARFE predictor model can only be used after the selector. The selected predictors are used for training a Random Forest algorithm. When new samples are provided for prediction, it first needs to pass through four steps. First predictors (columns) that were not selected are removed from data. Then the data goes through an imputation method that will replace missing predictors by its most correlated available predictor. The Pearson's correlation on the training CLR-transformed data (CODARFE-selector) is then measured. If a strongly correlated predictor ($\rho >= 0.7$) is present in the new samples, it will replace the missing one, otherwise the value is set as zero. Finally, the data is CLR-transformed and predicted by the trained Random Forest and the results are obtained (Figure 2B).

Imputation method

A missing data imputation method was developed to mitigate the issue of dealing with predictors selected by the model which are not present in a new dataset. Four methods were tested. The first method ("zero") considered the abundance of the missing predictor as 0. The second method ("corr") created a list containing "K" possible imputation predictors, where each one had a strong cor- relation ($\rho >= 0.7$) with the missing one. The Pearson correlation coefficient was calculated from the CLR transformed data, and the value of "K" arbitrarily set to 50. The presence of "K" was to increase the chance that at least one predictor was available in the new samples. The value of the predictor with the highest

correlation found in the new sample is used to substitute the value of the missing predictor. However, this method did not ensure the existence of a correlated attribute, returning zero in such cases. The third method ("phylo") selected the most similar taxa in a phylogenetic tree generated from the union of the new sample with the training set. The distance between two nodes was calculated using the "get-distance" function from the et3 library in Python version 3.10.6. The selected predictor's value is assigned in place of the missing predictor. The fourth method ("phyloclade") was similar to the third method, however it was limited to the clade in which the missing attribute was placed. To test each method, the database 'aggregate-stability' present in "Group A: Literature" was used. It was divided in an 80-20 scheme where 80% of the data were used for training a Random Forest and 20% were used for testing. The test data was filtered to contain only the selected predictors. Then it had 1% of its columns removed to simulate missing predictors. After, each method was used to replace the missing ones, and the Random Forest model predicted the results. This process was repeated until there were no predictors left. The Mean Absolute Error was used to choose the imputation method which led to the lowest error rate. Figure 2C illustrates the complete process.

Figure 2. Workflow for CODARFE-Prediction module and the workflows for selecting Random-Forest (RF) as prediction algorithm and correlation (Corr) as the imputation method. (A) A view of the process used to select the Random Forest as a prediction algorithm. First a training database was split so that 80% of the data was used for training and 20% for testing; Then the attributes most associate to target variable were select using the CODARFE-selector; A total of 1243 combinations of ML algorithms and hyper-parameter were tested and the Mean Absolute Error (MAE) was measured with 10 repetitions; As a result, a table of MAEs was created and the algorithm with lowest average MAE was selected as prediction model (RF). (B) The user database is used to select the predictors associated with the target through the CODARFE-selector and then training a Random Forest model. New samples are preprocessed and followed through the imputation step to be used as input for the RF model. The result is the prediction of the new sample's target variable. (C) The techniques evaluated for replacing (inputting) missing taxa. Four methods were tested in a recursive process where 1% of the total variables from a test dataset was removed on each step simulating increasing missing predictors. Each imputation method was used to replace the missing taxa creating a new dataset for the prediction. This new dataset was used to predict the target and the MAE error was measured. The method with the lowest average MAE was selected as the imputation method.

Comparison against four state-of-art tools

A comparison was done between CODARFE and four other tools in terms of performance on calculating/extracting/evaluating associations between the microbiome and environmental variables. The following describes the tools and how they were used. These

tools represent significant advancements in microbiome analysis, each with its advantages and limitations (Table 1). Every tool, including CODARFE, was trained with its default parameters. No fine tuning was performed for any tool to ensure a fair comparison. BRACoD employs Bayesian regression models [17] to find biological markers of diseases. As recommended by the authors, we used BRACoD with its default settings and filtered the input data to keep only the top 300 most prevalent bacteria. Coda4Microbiome takes into account the inherent variability of count data and uses hierarchical models based on the Bayesian posterior approach [18], while Selbal uses the balances method to identify co-abundance patterns among microbiome species [42]. For both tools all parameters were set to default, and the "nearZeroSum" function (R package “caret”) was used to filter the input data [43]. Finally, CLR-LASSO uses the CLR transformation for Euclidean representation of data and penalized LASSO regression for variable selection [19]. Nonetheless it requires the user to set up a lambda value that was defined as the value that mimics the most the number of variables selected by CODARFE.

	**CODARFE**	**Coda4microbiome**	**BRACoD**	**Selbal**	**CLR-LASSO**
**Filtering method**	RFE	Elastic-Net	MCMC	Balances	LASSO
**Transformation**	CLR	ILR*	CLR	ILR*	CLR
**Limit of columns**	No	No**	300	No**	No
**Create graphics**	Yes	Yes	No	No	No
**Prediction feature**	Yes	No	No	No	No

Table 1. Comparisons of tools used in this study for performance benchmark. Each tool has its own mode of operation, which is detailed in this table. *Isometric Log-Ratio (ILR). ** Theoretical limit does not exist, but it has a quadratic computational time in relation to the number of columns.

Simulating CODARFE’s prediction in identical studies

For the simulations, the datasets from group A were used. For each dataset, 20% of the samples were randomly removed to calculate the mean absolute error (MAE), and 80% were used for model training. This procedure was repeated 10 times, and the average MAE was calculated. The accuracy was measured through the Mean Absolute Percentage Error (MAPE) (equation 2).

$$MAPE = \frac{\frac{1}{N}\sum_{1}^{N} |real_i - pred_i|}{mean(target)} \quad \textbf{(2)}$$

where, "N" is the total number of samples, $real_i$ represents the true value in the position "i" and $pred_i$ represents the predicted value in the position "i".

Cross-studies prediction simulation

To fairly capture the predictive power, we used the cross-studies approach. We gathered datasets from several projects with different topics, but with at least one environmental variable that could serve as a prediction target included in their metadata. Then we trained the CODARFE - predictor in one dataset and used it to predict the target from the others datasets. The predictive power was measured with the following metrics: R^2 values to gauge model generalization, error percentages (equation 2) to quantify predictive accuracy, and the percentage of missing taxa in test datasets that was impossible to replace (imputation method returned zero). This simulation was conducted using the data from 'Group C: cross-studies', consisting of two subsets, and all taxa were identified by their taxonomy.

The first subset consists of two separate soil experiments with different environmental contexts, still both sequenced identical 16S rRNA variable regions and assessed the pH of the soil. The second subset consists of four human studies, each with different primary research objectives and experimental protocols (sequenced 16S rRNA variable regions, data collection techniques, and material type), nevertheless in all cases patient age was present in the metadata, which was used as a prediction target.

Comparison of computation complexity and running time

To measure the computational time of CODARFE and compare its performance against other tools, a benchmark was conducted using simulated compositional data organized in two sets of data, as follows: (i) containing one hundred predictors, while the number of samples varied; and (ii) fixed number of samples (n=50), while altering the number of predictors. All experiments were conducted on a Linux Ubuntu 24.04 server with the physical configuration of Xeon E5-2620 v4@2.10GHz and 192GB [2133Mhz DIMM DDR4 Dual Rank] Dell PowerEdge R630. After the fifth day, Coda4Microbiome and Selbal algorithms were prematurely terminated due to excessive time required for each analysis. Only the datasets completed by all tools were considered for comparison.

Results and Discussion

CODARFE has linear runtime, and pure correlation outperforms phylogeny for imputation

Figure 3 shows the computational time used to perform the analysis for each tool. Regarding the number of samples, BRACOD demonstrated a considerable increase in runtime correlated with increased sample sizes, Coda4Microbiome and CLR-LASSO exhibited the lowest runtimes. Selbal had a moderate upswing in runtime, followed closely by CODARFE, but none surpasses 400s (6 minutes) per database.

Regarding the number of predictors, the data transformation used by Coda4microbiome and Selbal tools consumes quadratic time, increasing its computational costs and limiting its usage of datasets with high numbers of predictors (e.g.: >1500 predictors), which can become a problem since the growth of the number of predictors (columns) is being driven by the development of more data-generating sequencing techniques [58]. The BRACoD's execution time significantly increases with the number of

predictors. Furthermore, there is a limitation of 300 predictors suggested by the authors together with the “solution” of selecting just the top 300 most abundant taxa in the dataset. This may raise a fundamental issue in microbiome, which is that the most abundant taxa may not be the most relevant to the target variable [59].

While CLR-LASSO has the lowest time in both cases, interpreting its results can be challenging due to the applied transformation and the effect of the mean of non-selected variables. Since this transformation is used in the whole dataset and just a few predictors are selected, the analysis of the method suffers effects from the unselected predictors. Furthermore, the user’s choice of the value of lambda directly affects the number of selected predictors, and it lacks generalization capability as evidenced by previous trials.

Finally, CODARFE remain computationally viable even when a high number of predictors (e.g., > 1500) are utilized, making it the best choice for largest datasets.

Figure 3. Time consumption comparison between tools regarding number of samples (rows) and number of predictors (columns) in the training dataset. The graph on the left depicts the time in seconds required by each tool to finish its process as the number of samples in the database increases. The graph on the right depicts the time in seconds required by each tool to finish its process as the number of predictors in the database increases.

Regarding the imputation method, missing predictors is not a common problem in the field of machine learning, as one of the processes in the traditional ML pipeline is predictor extraction [44]. In this case the predictors are the taxonomic labels that are used to train the model, but are not necessarily present in the new samples, making the prediction very unstable [45]. Two possibilities were considered to address this problem: the abundance-correlation and the phylogenetic relationship. Although the abundance-correlation may have statistical significance, it might lack biological meaning, as having the same abundance does not necessarily mean two bacteria have the same biological functions [46]. Additionally, there is no guarantee that a high correlated bacteria will exist in the new sample. The phylogenetic relationship approach is based on the idea that if two bacteria are closely related evolutionarily, there is a possibility that they are fulfilling a similar biological role [47]. Although the phylogenetic tree is constructed using both new samples and the training

dataset, it is possible that the closest evolutionarily related bacterium may not be 'close enough' [48] to have the same function.

As shown in Figure 4, the abundance-correlation method obtained the lowest mean absolute error concerning the percentage of missing data, making it the chosen imputation method for the tool. The imputation method works by replacing a missing taxa for an existing one, for example, if bacteria "A" from train dataset 1 is not present in dataset 2, it can be replaced with bacteria "B" from dataset 2 if B has a high correlation with A. Additionally, if more than 75% of the missing bacteria (from dataset 1) cannot be replaced by bacteria found in dataset 2, CODARFE immediately stops the prediction.

There may be at least two hypotheses for why the correlation method outperformed the others: (i) The chosen bacterium's abundance value was closer to the original bacterium's value, which helped the model correctly predict the target variable. Although the biological explanation suggested the use of techniques based on phylogenetic distance, the abundance values between closely related species can be very different. Since the model does not consider the biological aspect and relies solely on abundance values, pure correlation will tend to yield better results; (ii) The second hypothesis is based on the redundancy of biological roles within the microbiome. In this hypothesis, two bacteria with a strong correlation of abundance may possess this similarity precisely because they have redundant roles [49].

Figure 4. The results of total MAE by percentage of missing taxa for each one of the four tested imputation methods. The database used for testing was the 'aggregate-stability' from "Group A: Literature", with targets ranging from 1.16 to 91.37 units with an average of 21.08 and standard deviation of 17.48. Starting with all taxa, on each step 1% of the total taxa are removed until there is no more taxa present. On each step the MAE is calculated for each one of the four tested imputation methods (Zero, Corr, Phylo and PhyloClade). The MAE is shown in the y-axis and the percentage of missing taxa is presented in the x-axis.

CODARFE outperformed other tools regarding data generalization and proportions of correctly selected taxa

Regarding data generalization, two groups of datasets were used for benchmarking: 19 soil health metrics (Group A: Literature) and 5 human diseases (Group B: ML Repo). For "Group A: Literature", CODARFE achieved the best correlation between the generalized and real values for 18 out of the 19 measurements. BRACoD and Coda4Microbiome showed similar results (0.03 units on average), but the latter failed to generalize the amount of phosphorus ("P"), returning "not a number" (nan) during the process. Selbal had the lowest correlation coefficient among all methods, being unable to generalize the data for 4 out of the 19 measurements, namely "P", "Mn", "subsurface hardness", and "surface hardness" (Figure 5A) (The complete data distribution is shown in Figure S1.2).

For "Group B: ML Repo", CODARFE outperformed the other tools by at least 0.2 in terms of R^2 for the pediatric Crohn's disease dataset. Coda4Microbiome failed to generalize the data for the samples taken from the Ileum, and the CLR-LASSO was unable to generalize any of the datasets. Regarding the vaginal health datasets, BRACoD and Coda4Microbiome outperformed CODARFE (Figure 5), suggesting a complex pattern that is difficult for CODARFE to understand.

Except for pH measurements and Nugent scores, CODARFE outperforms all others in the "learned vs real" correlation. This suggests that the combination of the coefficient shrinkage method with RFE has a higher ability to associate predictors with the target, as previously proposed in [50], where a similar proposal was used and compared with other methods for cervical cancer prediction. Methods primarily based on coefficient shrinkage, such as Coda4microbiome and Selbal, do not test for subsets smaller than the shrinkage suggested. These subsets may hide combinations of predictors that provide better insights. This problem can be mitigated through more exhaustive tests, such as using RFE.

Finally, the lower performance from CODARFE in the vaginal pH and Nugent score datasets may be attributed in part to the way the values were measured. Both datasets had 388 samples, but there were only 11 distinct Nugent score values and 7 pH values. Such a low data variation can affect the generalization performance [51] and make the problems more classifiable rather than prone to regression.

Figure 5. Performance comparison of different machine learning tools for soil and human health prediction. Panel (A): Shows the performance on 19 soil health metrics. CODARFE achieved the best correlation for 18 metrics. BRACoD and Coda4Microbiome had similar performance, but Coda4Microbiome failed to predict phosphorus (P). Selbal had the worst performance. The complete distribution of the data is available in Figure S1.2.Panel (B): Shows the performance on five human disease datasets through histograms of frequency distribution. A distribution similar to the real one indicates good generalization ability of the model. CODARFE outperformed other tools for pediatric Crohn's disease. Coda4Microbiome failed to predict ileum samples, and CLR-LASSO failed on all datasets. BRACoD and Coda4Microbiome outperformed CODARFE for vaginal health datasets. Panel (C): Shows the R^2 values for each method on all datasets. CODARFE achieved the best overall performance except for pH and Nugent score.

In order to verify the relevance of the taxa selected by the different tools, we performed a more in-depth analysis of the human microbiome data contained within the "Group B: ML Repo". The three publications that were used are each briefly detailed here, and the description of the taxa that were discovered to be connected to the interest variable are available at Supplementary material 2 (S2).

Yatsunenko et al. (2012) conducted a study utilizing fecal samples from diverse populations, including healthy Amerindians from the Amazon and Venezuela, rural communities in Malawi, and metropolitan areas in the United States. Their aim was to characterize postnatal developmental stages and their correlation with environmental factors. Although the study did not explicitly seek direct associations between the gut microbiome bacteria and postnatal age, Bifidobacteruim was pointed out by the authors as a potential biomarker for infant age. As shown in green in the bar plots (Figure 6), only CODARFE and BRACoD selected Bifidobacterium species, with CODARFE selecting 41 times less false-positives than BRACoD. The other selected taxa (red) are not supported by the original article.

Ravel et al. (2010) characterized bacteria present in vaginal samples from four ethnic groups (White, Black, Hispanic, and Asian) to ascertain vaginal pH and Nugent scores. In total, 20 phylotypes were highlighted as related to Nugent score, 19 being related to high levels and one to low levels. BRACoD and Coda4Microbiome correctly selected a total of 8 different phylotypes; CODARFE and Selbal correctly selected 5 phylotypes; and CLR-LASSO correctly selected a total of 4 phylotypes (the difference between taxa selected by each tool is described in S2). For vaginal pH, only the presence or absence of Lactobacillus

was reported to be relevant, with its presence being inversely proportional to the pH measurement. BRACoD selected 3 species; Coda4Microbiome selected 6 species; CODARFE selected 4 species; CLR-LASSO selected 2 species; and finally Selbal selected 2 species. It is worth noting that the proportions of correctly selected and total selected vary drastically between tools (Figure 6).

The work developed by Gevers et al. (2014) characterized bacteria present in ileum and rectal samples to understand recent cases of Paediatric Crohn's disease through its activity index (PCDAI). The authors identified 21 species with a high correlation to PCDAI. The results in figure 6 are shown for two separated datasets (ileum and rectum), but all species are valid for both datasets. BRACoD correctly selected 3 species for the Ileum dataset and 3 species for rectum dataset; As previously described, Coda4Microbiome was unable to finish its process for the Ileum dataset, and did not select any of the 21 species pointed out by the article in the rectum dataset; CODARFE correctly selected 4 species for ileum dataset and 4 species for rectum dataset; CLR-LASSO selected only one for ileum and rectum; and Selbal did not select any of the 21 species.

In these three cases, CODARFE finds more species highlighted in the original articles compared to other tools (green bar chart in figure 6), suggesting that it has lower False Discovery Rate (FDR) than the other tools on average. We believe that CODARFE was able to achieve these results due to the combination of metrics with the RFE [53]. Two of the metrics are strong indications of a high quantity of predictors, being the R^2 adjusted and the BIC metrics [54, 55]. Both these metrics account as a penalization metric for the number of predictors selected, giving high scores as the number of predictors decrease. In addition, we combined them with the ρ-value of the F-test in the final predictors score. This value basically indicates how beneficial the inclusion of a predictor is to the model (it can be thought of as a value representing "how much these predictors explain the target"). Since these three metrics are weighted and summed together, the final score will indicate "how worthwhile it is to keep all these predictors in relation to how much they explain". This value is weighted with an "overfitting value" and used to evaluate all (at most) 100 predictors sets

during the RFE step, increasing the chance of finding a subset of predictors with optimal scores that are not explored by other methods (such as shrinkage used by Coda4Microbiome) [39].

Figure 6. The figure illustrates the performance of all five tools for selecting relevant taxa identified in three different studies (with 5 different target variables) on the human microbiome. The bars show the number of taxa correctly selected by each tool (blue) and the number of taxa not supported by the original study (orange) for each dataset. CODARFE generally selected more taxa identified in the original studies compared to other tools, and also had a lower proportion of false positives (taxa not supported by the studies) compared to other tools as illustrated by the last panel (green).

Simulating prediction in identical and cross studies

The next test involved simulating studies through the hold-out method to simulate predictions. Equation 2 was used to calculate the measure of error (MAPE), which expresses the error as a percentage of each target variable's mean. Using data from "Group A: Literature" (Figure 7), "surface-hardness" resulted in the lowest error of 0.01% of the target's mean (target range: 0.001 - 743.126), followed by "Zn" with 3.37% (target range: 0.001 - 23.617) and "soil-texture clay" with 6.21% (target range 0.356 - 39.436). The highest error was found for "respiration" with 47.88% (target range 0.077 - 1.781).

One hypothesis for this arising pattern is related to the data distribution. The "respiration" metric had the narrowest distribution of all the metrics (Figure S1.3), varying only 1.7 units from minimum to maximum with a standard deviation of 0.23 units, making any deviation from the prediction to the true value to result in a high error. In contrast, the "surface_hardness" had one of the most widely distributed distributions, ranging from 0 to over 700 units with a standard deviation of 105 units. On top of that, the narrow amplitude has high peaks of density, which may create a bias in the model reflecting in even higher errors when the true value is outside the high density range [52]. On the other hand, most of the results obtained were superior (in terms of R^2) than the reported in the original article [21], supporting the high predictive power of CODARFE in most of the scenarios.

Figure 7. Mean Absolute Percentage Error distribution for soil health and fertility metrics. Individual values across a 10-repetition hold-out validation are represented by the dots, and the average is indicated as the bar. It is possible to see a trend related to the target amplitude, where a narrow distribution has the highest error rate ("respiration") and a wide distribution has the lowest error ("surface_hardness").

For the cross-studies prediction, we separated "Group C: cross-studies" into two groups, one similar to each other and one highly different, in order to better examine the effects of studies that were similar and studies that were quite different in many areas, such as subject of investigation, region of sequencing, data collection, external effects, and others (the differences between each dataset are indicated in Tables 2 and 3). To assess CODARFE's robustness we used the R^2 values to judge model generalization (Figures 8C and 9B), mean absolute percentage error (MAPE) to quantify predictive accuracy (Figure 8B and 9C), and the percentage of missing taxa in test datasets (imputation method returned zero) to verify the effect of missing taxa in the final prediction (Figure 9D).

In the first subset, Chroňáková (2015) evaluated the response of soil bacterial communities to changes associated with outdoor cattle overwintering, while Nguyen et. al. (2016) analyzed how bacterial diversity and community structure in Korean ginseng fields were altered by cultivation time. Both datasets achieved high R^2 (Figure 8C) which indicates a high predictive power. For the Chroňáková (2015), the error percentages were observed to be 4.17% and 10.78% (Figure 8B) when tested on itself and the Nguyen et. al. (2016) dataset, respectively. Similarly, the Nguyen et. al. (2016) exhibited error percentages of 5.33% and 18.67% (Figure 8B) when tested on the Chroňáková (2015) dataset and itself, respectively.

Both the Chroňáková (2015) and Nguyen et. al. (2016) datasets were generated from the 16S rRNA V1-V3 region (Table 2), and all the predictors (taxa) selected by CODARFE were present in both studies (in the intersection, Figure 8A), meaning that no imputation was needed, and reinforcing the idea that pH related taxa are similar for various types of soils [56]. This will have helped with the low error even considering the agent of the study effect is entirely different, and the soil samples are at least 8,000 kilometers apart. The sequenced region directly influences the assigned taxonomy [57], which is then used as a

predictor by the tool, since, if different taxonomies are assigned to the same predictor, they will be considered as different predictors. With these results, a key factor influencing the observed prediction errors may be the choice of amplified region, since both the Chroňáková (2015) and Nguyen et. al. (2016) datasets were generated from the V1-V3 region (Table 2).This factor is better observed in the second subset.

	**Chron´akov´a et. al. (2015)**	**Nguyen et. al. (2016)**
**Sample source**	Soil	Soil
**Aim of the study**	Investigation of prokaryotic diversity in soil impacted by outdoor cattle husbandry	Investigation of bacterial communities in the soil of ginseng fields
**Location**	Czech republic	South Korea
**Year of collection**	2011	2012
**Sequenced region**	V1-V3	V1-V3
**MGnify study identifier**	MGYS00000916	MGYS00001160

Table 2. Details of each dataset used in the soil cross-studies analysis.

Figure 8. Cross-studies prediction for tow soil datasets measuring pH. The number of identical predictors in each study are shown in (A). The percentage of mean absolute error in relation to the average of the target variable when training CODARFE with data from one work and predicting another is shown in (B). The coefficient of determination (R^2) obtained in the generalization of the CODARFE model is shown in (C).

In the second subset, four completely different studies were used (Table 3). Raymond et al. (2016) model demonstrates the lowest error when tested on itself (8.10%) (Figure 9C) and exhibits the lowest error on average (53.41%) when predicting others datasets. Conversely, the model by Di Paola et al. (2016) yields the highest average error (80.11%) (Figure 9C).

The best results yielded an error of 32% of the mean (Figure 9C), indicating low predictive power between studies. A reduced R^2 in these studies suggested that the microbiota may be more influenced by the study object than by age. Furthermore, although the case with the best error (32%) has a 25% missing taxa rate (Figure 9D), it is not only the absence of taxa that affects the error rate, as shown by the model trained with Di Paola et. al. (2015), which was unable to predict the age accurately in other works, despite containing

most taxa. This may be partially explained by the fact that this work had the lowest fitting power (R^2) (Figure 9B) among all, leading us to believe that the error may be a combination of fitting power with missing taxa and sequencing region.

The influence of missing taxa becomes evident when comparing the models trained on datasets with fewer missing taxa to the highest ones. This trend can be observed with the Wang et al. (2020) model trained on the dataset with the lowest missing taxa percentage (0%) also demonstrating the lowest average error across test datasets (Figure 9C and D). This trend is reaffirmed by the challenges faced by the Wang et. al. (2020) and Noguera et. al. (2016) models in predicting the data of Di Paola et. al. (2016) highlight the effects of missing taxa and sequencing regions in the model effectiveness. The percentage of missing taxa in the Di Paola et. al. (2016) dataset (Figure 9D) is substantially higher compared to the training datasets. The Wang et. al. (2020) model, trained on V3–V4 region, and the Noguera et. al. (2016) model, trained on 16S/18S rRNA (Table 3), might not have adequately captured predictors relevant to the V5-V6 region utilized by the Di Paola et. al. (2016) dataset (Figure 9A and D). This may suggest a critical relationship between missing taxa, and predictive accuracy.

Further examining the sequencing regions used to generate the data, it again suggested that different regions can impact the predictive performance. For instance, the Noguera et al. (2016) model, trained on 16S/18S rRNA data (Table 3), exhibits relatively strong cross-dataset performance, while the Raymond et al. (2016) model, trained on the V3 region (Figure 9C) showcases the highest R^2 value and lowest error on average. Another indication that the sequenced region is an essential factor in the tool's proper functioning comes from the cross-study analyses of Di Paola et. al. (2016) and Wang et. al. (2020). These two datasets did not share the 16S sequenced region and resulted in the highest error rates. Conversely, the models created from Wang et. al. (2020) and Raymond et. al. (2016), both sequencing the V3 region, when used to predict Noguera et. al. (2016) study, which sequenced the complete 16S, yielded errors of 34% and 41%, respectively, which are relatively low compared to its other case with 275% error.

	**Di Paola et. al. (2016)**	**Raymond et. al. (2016)**	**Wang et. al. (2020)**	**Noguera et. al. (2016)**
**Sample source**	Large intestine	Large intestine	Digestive system	Large intestine
**Sample type**	Feces	Feces	Gastric mucosa	Feces
**Aim of the Study**	Characterization of gut microbiota in Juvenile Idiopathic arthritis	Study of the impact of antibiotics on the gut microbiome	Microbiota in patients with gastritis, intestinal metaplasia, early gastric cancer and advanced gastric cancer	Understanding the impact of HIV on the microbiome
**Platform Seq.**	Illumina	Illumina	Illumina	Illumina
**Sequenced region**	V5-V6	V3	V3–V4	16s/18s rRNA
**Host**	Children	Adults between 21 and 35 years old	Adults	Adults
**MGnify study identifier**	MGYS00000580	MGYS00001175	MGYS00001188	MGYS00001255

Table 3. Table exhibiting pertinent data from each research paper used in the human cross-studies analysis.

Figure 9. Cross-studies prediction for four very distinct human datasets measuring the human age. The number of identical predictors (taxa) in each study is shown in (A); The coefficient of determination (R^2) obtained in the generalization of the CODARFE model is shown in (B); The percentage of mean absolute error in relation to the average of the target variable when training CODARFE with data from one work and predicting another is shown in (C); The percentage of predictors in the prediction dataset that were unable to be replaced by the imputation method (the imputation method returned zero) is shown in (D).

The CODARFE addresses the batch effect in these cross-study analyses. The batch effect—particularly its most severe manifestation—arises when different experimental batches are combined, creating a spurious correlation between the batch and the target variable. This issue is mitigated in the present study because the models were trained on a single dataset without batch mixing. Notably, the pH-model selected only bacterial taxa present in both datasets, even without their integration, suggesting that it successfully

identified bacteria specifically associated with pH. Additionally, any batch effects inherent to a single dataset would affect all samples within that dataset uniformly, rendering them less informative. Consequently, such effects are likely to be eliminated during the preprocessing phase.

Feasibility and limitations

Based on the results obtained, we elaborated seven hypotheses regarding CODARFE feasibility and limitations:

(i) The main factor determining predictive power is the number of predictors that could not be replaced by the suggested imputation method, as observed in the models trained with Wang et. al. (2020) or Noguera et. al. (2016) datasets and tested in Di Paola et. al. (2016) and Raymond et. al. (2016) datasets. These models had the highest associated error and the highest percentage of missing predictors. Considering such a limitation, it is likely that the sequenced region needs to be the same for better results since the sequenced region directly affects the assigned taxonomy. Furthermore, we made CODARFE automatically stop the prediction if the number of missing taxa is greater than 75%.

(ii) The samples from which the sequences are extracted should be similar, as the microbiome is closely linked to the environment. Thus, samples from different types (e.g.: Feces and gastric mucosa) also negatively impact the model's predictive ability, as seen in the models trained with Wang et. al. (2020) dataset.

(iii) The "objective of the study" for new samples should be similar to that used for training. For example, the model generated from Di Paola et. al. (2016) , which focused on children, could not accurately predict ages in other studies, indicating that the taxa associated with infant age differ from those associated with adult age, supporting the idea discussed in [23].

(iv) To build a robust model, the metadata must be complete, just like in any machine learning process. For instance, samples with sick children should not be included in the metadata if the researcher wants to build a model that links health taxa to infant age, since the model might learn on this bias from these samples. However, if the model was properly constructed and the new sample's prediction deviates significantly from the actual value (if you know it), it may indicate the existence of a disease.

(v) Regarding the adequacy of the training datasets, the metrics offered by CODARFE can be used to determine whether the present sample size is inadequate. For instance, if the hold-out test's R^2 is insufficient (~<0.4) or the p-value is not significant (>= 0.05), the user will be aware that the samples are insufficient and will have the freedom to decide whether or not to utilize the model

(vi) The current version of CODARFE only performs regression for a single variable when it comes to controlling the effects of multiple variables. If the researcher is interested in the association between the microbiome and a numerical variable while ignoring the effects of a confounder variable, one way to do this would be to apply CODARFE to the residuals of a regression between the two variables. This logic even allows CODARFE to be expanded to other types of variables. Applying a logistic transformation on relative data, for instance, can result in a logistic CODARFE.

(vii) CODARFE can be applicable to data other than taxonomic units, such as protein functions and metabolic pathways since the mathematical constraints are the same for all of them. As proof of concept, a 10-repetition train-test split on two different projects collected from MGnify linking GO and InterPRO terms to water temperature are made available in Supplementary material 3 (S3).

Conclusion

We present CODARFE, a powerful tool capable of selecting sparse compositional predictors and associating them with continuous environmental variables, which in turn can

be used to predict these variables in new samples. CODARFE's performance was benchmarked against four other tools and exhibited superior results in 21 out of 24 tested datasets, with over 0.1 units stronger in terms of R^2 on average, and 17 times faster than the most recent published tools, particularly when the number of predictors surpassed 1500. For human-related data, CODARFE achieved the highest number of identified taxa linked to the target variable that were also identified in the publication describing the study. Furthermore,we demonstrated CODARFE's predictive power in cross-study analyses, where a model trained on data from one project could be successfully used to predict data from another project, with a maximum error rate of 18% in relation to the target variable mean, as long as the sequence region and sample type are the same. These results indicate CODARFE's potential for generalization and robustness across multiple studies, even under different experimental settings. Furthermore,the CODARFE uniqueness lies in its ability to predict the target variable in new samples, due to its imputation method for missing predictors. This feature allows for predicting environmental variables in previously unexplored contexts, providing researchers with a reliable and versatile tool.

Availability of source code and requirements

- Project name: CODARFE
- Project home page: https://github.com/alerpaschoal/CODARFE
- Operating system(s): Platform independent (except for the graphical user interface, which was developed specifically for Windows)
- Programming language: Python
- Other requirements: Python 3.10 or higher
- License:Apache License Version 2.0

Data availability

All tool versions can be found at https://github.com/alerpaschoal/CODARFE, and https://doi.org/10.5281/zenodo.12751711 contains the data and code used to create all of the analyses and plots.

Declarations

List of abbreviations

- CLR: Centered-Log-Ratio
- CoDA: Compositional Data Analysis
- CV: Coefficient of Variation
- ILR: Isometric Log-Ratio
- MAE: Mean Absolute Error
- MAPE: Mean Absolute Percentage Error
- MHT: Model-Hyperparameter-Transformation
- ML: Machine Learning
- PCDAI: Paediatric Crohn's disease Activity index
- RF: Random-Forest
- RFE: Recursive Feature Elimination
- RMSE: Rooted Mean Squared Error

Competing Interests

LCA and JFMS are employees of SUPERBAC Biotechnology Solutions, however the company did not influence any aspects of the study, besides funding.

Funding

- Fundação Araucária, NAPI Bioinformática, #66.2021, A R Paschoal;
- CNPq, #440412/2022-6, A R Paschoal;
- European Bioinformatics Institute, R D Finn;

- SUPERBAC Biotechnology Solutions, PhD Fellowship, 02,2021 M C Barbosa;
- CAPES, PDSE (PDSE - Programa de Doutorado Sanduíche no Exterior), 88881.846275/2023-01, M C Barbosa;

Author's Contributions

- Conceptualization: MCB, ARP, LCA, JFMS
- Methodology: MCB, ARP, LCA, JFMS
- Software development: MCB
- Validation: all authors.
- Revision: all authors.
- Writing: all authors.

Acknowledgements

Authors would like to thank the "Postgraduate Associate Program in Bioinformatics (PPGAB)" between UTFPR and UFPR and the Group of Pattern Recognition and Bioinformatics also from UTFPR for the mentoring.

References

1. Gilbert JA, Blaser MJ, Caporaso JG, Jansson JK, Lynch SV, Knight R. Current understanding of the human microbiome. Nature medicine 2018;24(4):392–400.

2. Novikova N, De Boever P, Poddubko S, Deshevaya E, Polikarpov N, Rakova N, et al. Survey of environmental biocontamination on board the International Space Station. Research in microbiology 2006;157(1):5–12.

3. Johnson CL, Versalovic J. The human microbiome and its potential importance to pediatrics. Pediatrics 2012;129(5):950–960.

4. Lavelle A, Hill C. Gut microbiome in health and disease: emerging diagnostic opportunities. Gastroenterology Clinics 2019;48(2):221–235.

5. Wen C, Zheng Z, Shao T, Liu L, Xie Z, Le Chatelier E, et al. Quantitative metagenomics reveals unique gut microbiome biomarkers in ankylosing spondylitis. Genome biology 2017;18:1–13.

6. Damhorst GL, Adelman MW, Woodworth MH, Kraft CS. Current capabilities of gut microbiome–based diagnostics and the promise of clinical application. The Journal of Infectious Diseases 2021;223(Supplement_3):S270–S275.

7. Suman J, Rakshit A, Ogireddy SD, Singh S, Gupta C, Chandrakala J. Microbiome as a key player in sustainable agriculture and human health. Frontiers in Soil Science 2022;2:821589.

8. Legendre P, Gallagher ED. Ecologically meaningful transformations for ordination of species data. Oecologia 2001;129:271-280.

9. Lin H, Peddada SD. Analysis of microbial compositions: a review of normalization and differential abundance analysis. NPJ biofilms and microbiomes 2020;6(1):60.

10. Gloor GB, Macklaim JM, Pawlowsky-Glahn V, Egozcue JJ. Microbiome datasets are compositional: and this is not optional. Frontiers in microbiology 2017;8:2224.

11. Kurtz ZD, Müller CL, Miraldi ER, Littman DR, Blaser MJ, Bonneau RA. Sparse and compositionally robust inference of microbial ecological networks. PLoS computational biology 2015;11(5):e1004226.

12. Lutz KC, Jiang S, Neugent ML, De Nisco NJ, Zhan X, Li Q. A survey of statistical methods for microbiome data analysis. Frontiers in Applied Mathematics and Statistics 2022;8:884810.

13. Aitchison J. The statistical analysis of compositional data. Journal of the Royal Statistical Society: Series B (Methodological) 1982;44(2):139–160.

14. Ghannam RB, Techtmann SM. Machine learning applications in microbial ecology, human microbiome studies, and environmental monitoring. Computational and Structural Biotechnol ogy Journal 2021;19:1092–1107.

15. Huang S, Ailer E, Kilbertus N, Pfister N. Supervised learning and model analysis with compositional data. PLOS Computational Biology 2023;19(6):e1011240.

16. Chandrasekhar V. Disease2Vec: a method of determining disease from gut microbiome using neural embeddings. PhD thesis, Harvard University; 2020.

17. Verster A, Petronella N, Green J, Matias F, Brooks SP. A Bayesian method for identifying associations between response variables and bacterial community composition. PLoS Computational Biology 2022;18(7):e1010108.

18. Calle ML, Pujolassos M, Susin A. coda4microbiome: compositional data analysis for microbiome cross-sectional and longitudinal studies. BMC bioinformatics 2023;24(1):82.

19. Susin A, Wang Y, Lê Cao KA, Calle ML. Variable selection in microbiome compositional data analysis. NAR Genomics and Bioinformatics 2020;2(2):lqaa029.

20. Zhang W, Liu A, Zhang Z, Chen G, Li Q. An adaptive direction-assisted test for microbiome compositional data. Bioinformatics 2022;38(14):3493–3500.

21. Wilhelm RC, van Es HM, Buckley DH. Predicting measures of soil health using the microbiome and supervised machine learning. Soil Biology and Biochemistry 2022;164:108472.

22. Vangay P, Hillmann BM, Knights D. Microbiome Learning Repo (ML Repo): A public repository of microbiome regression and classification tasks. Gigascience 2019;8(5):giz042.

23. Yatsunenko T, Rey FE, Manary MJ, Trehan I, Dominguez-Bello MG, Contreras M, et al. Human gut microbiome viewed across age and geography. nature 2012;486(7402):222–227.

24. Gevers D, Kugathasan S, Denson LA, Vázquez-Baeza Y, Van Treuren W, Ren B, et al. The treatment-naive microbiome in new-onset Crohn’s disease. Cell host & microbe 2014;15(3):382–392.

25. Ravel J, Gajer P, Abdo Z, Schneider GM, Koenig SS, McCulle SL, et al. Vaginal microbiome of reproductive age women. Proceedings of the National Academy of Sciences 2011;108(supplement_1):4680–4687.

26. Richardson L, Allen B, Baldi G, Beracochea M, Bileschi ML, Burdett T, et al. MGnify: the microbiome sequence data analysis resource in 2023. Nucleic Acids Research 2023;51(D1):D753–D759.

27. Chroňáková A, Schloter-Hai B, Radl V, Endesfelder D, Quince C, Elhottová D, et al. Response of archaeal and bacterial soil communities to changes associated with outdoor cattle over wintering. PLoS One 2015;10(8):e0135627.

28. Nguyen NL, Kim YJ, Hoang VA, Subramaniyam S, Kang JP, Kang CH, et al. Bacterial diversity and community structure in Korean ginseng field soil are shifted by cultivation time. PloS one 2016;11(5):e0155055.

29. Di Paola M, Cavalieri D, Albanese D, Sordo M, Pindo M, Donati C, et al. Alteration of fecal microbiota profiles in juvenile idiopathic arthritis. Associations with HLA-B27 allele and disease status. Frontiers in microbiology 2016;7:1703.

30. Raymond F, Ouameur AA, Déraspe M, Iqbal N, Gingras H, Dridi B, et al. The initial state of the human gut microbiome determines its reshaping by antibiotics. The ISME journal 2016;10(3):707–720.

31. Wang L, Xin Y, Zhou J, Tian Z, Liu C, Yu X, et al. Gastric mucosa-associated microbial signatures of early gastric cancer. Frontiers in microbiology 2020;11:1548.

32. Noguera-Julian M, Rocafort M, Guillén Y, Rivera J, Casadellà M, Nowak P, et al. Gut microbiota linked to sexual preference and HIV infection. EBioMedicine 2016;5:135–146.

33. Ng KW, Tian GL, Tang ML. Dirichlet and related distributions: Theory, methods and applications 2011;.

34. Smith JK. Data transformations in analysis of variance. Journal of Verbal Learning and Verbal Behavior 1976;15(3):339–346.

35. Zheng A, Casari A. Feature engineering for machine learning: principles and techniques for data scientists. " O'Reilly Media,Inc."; 2018.

36. Chang CC, Lin CJ. LIBSVM: a library for support vector machines. ACM transactions on intelligent systems and technology (TIST) 2011;2(3):1–27.

37. Owen AB. A robust hybrid of lasso and ridge regression. Contemporary Mathematics 2007;443(7):59–72.

38. Dang X, Peng H, Wang X, Zhang H. Theil-sen estimators in a multiple linear regression model. Olemiss Edu 2008;.

39. Guyon I, Weston J, Barnhill S, Vapnik V. Gene selection for cancer classification using support vector machines. Machine learning 2002;46:389–422.

40. Shalev-Shwartz S, Ben-David S. Understanding machine learning: From theory to algorithms. Cambridge university press; 2014.

41. Breiman L. Random forests. Machine learning 2001;45:5–32.

42. Rivera-Pinto J, Egozcue JJ, Pawlowsky-Glahn V, Paredes R, Noguera-Julian M, Calle ML. Balances: a new perspective for microbiome analysis. MSystems 2018;3(4):10–1128.

43. Kuhn M. Building predictive models in R using the caret package. Journal of statistical software 2008;28:1–26.

44. Guyon I, Elisseeff A. An introduction to feature extraction. In: Feature extraction: foundations and applications Springer; 2006.p. 1–25.

45. Nijman S, Leeuwenberg A, Beekers I, Verkouter I, Jacobs J, Bots M, et al. Missing data is poorly handled and reported in prediction model studies using machine learning: a literature review. Journal of clinical epidemiology 2022;142:218–229.

46. Escalas A, Hale L, Voordeckers JW, Yang Y, Firestone MK, Alvarez-Cohen L, et al. Microbial functional diversity: From concepts to applications. Ecology and Evolution 2019;9(20):12000–12016.

47. Gill AS, Lee A, McGuire KL. Phylogenetic and functional diversity of total (DNA) and expressed (RNA) bacterial communities in urban green infrastructure bioswale soils. Applied and Environmental Microbiology 2017;83(16):e00287–17.

48. Sexton JP, Montiel J, Shay JE, Stephens MR, Slatyer RA. Evolution of ecological niche breadth. Annual Review of Ecology, Evolution, and Systematics 2017;48:183–206.

49. Rivett DW, Bell T. Abundance determines the functional role of bacterial phylotypes in complex communities. Nature microbiology 2018;3(7):767–772.

50. Hamada M, Tanimu JJ, Hassan M, Kakudi HA, Robert P. Evaluation of Recursive Feature Elimination and LASSO Regularization-based optimized feature selection approaches for cervical cancer prediction. In: 2021 IEEE 14th International Symposium on Embedded Multicore/Many-core Systems-on-Chip (MCSoC) IEEE; 2021. p. 333–339.

51. He H, Garcia EA. Learning from Imbalanced Data. IEEE Transactions on Knowledge and Data Engineering 2009;21(9):1263–1284.

52. Gyori NG, Palombo M, Clark CA, Zhang H, Alexander DC. Training data distribution significantly impacts the estimation of First et al. | 15 tissue microstructure with machine learning. Magnetic resonance in medicine 2022;87(2):932–947.

53. Guyon I, Elisseeff A. An introduction to variable and feature selection. Journal of machine learning research 2003;3(Mar):1157–1182.

54. Schwarz G. Estimating the Dimension of a Model. The Annals of Statistics 1978;6(2):461 – 464. https://doi.org/10.1214/ aos/1176344136.

55. Schwarz G. Estimating the dimension of a model. The annals of statistics 1978;p. 461–464.

56. Bååth E. Adaptation of soil bacterial communities to prevailing pH in different soils. FEMS Microbiology Ecology 1996;19(4):227–237.

57. Guo F, Ju F, Cai L, Zhang T. Taxonomic precision of different hypervariable regions of 16S rRNA gene and annotation methods for functional bacterial groups in biological wastewater treatment. PloS one 2013;8(10):e76185.

58. Quince C, Walker AW, Simpson JT, Loman NJ, Segata N. Shotgun metagenomics, from sampling to analysis. Nature biotechnology 2017;35(9):833–844.

59. Woodcock S, Curtis TP, Head IM, Lunn M, Sloan WT. Taxa-area relationships for microbes: the unsampled and the unseen. Ecology letters 2006;9(7):805–812.

Figure1

Click here to access/download;Figure;Fig1.png

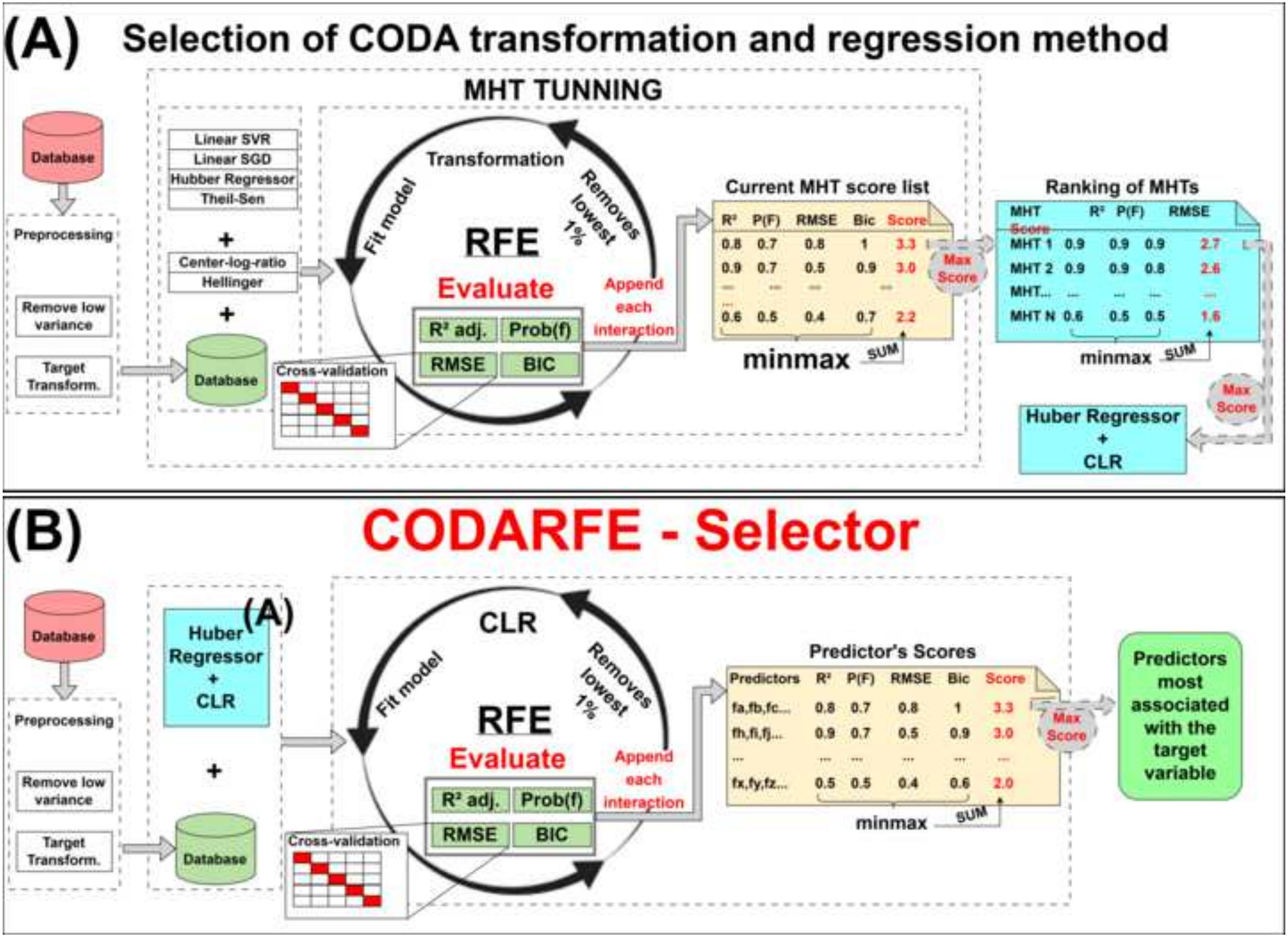

Figure2

Click here to access/download;Figure;Fig2.png

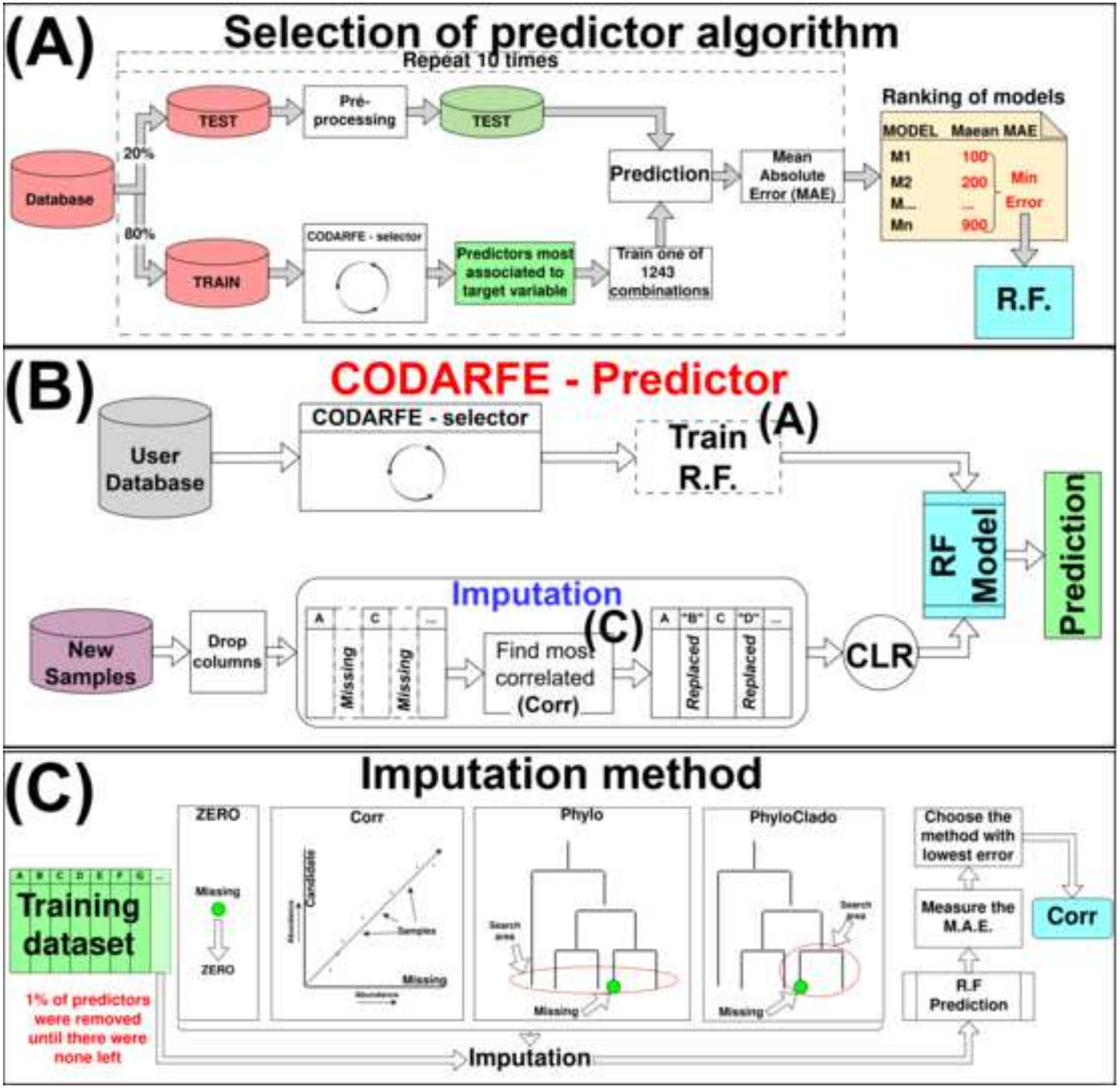

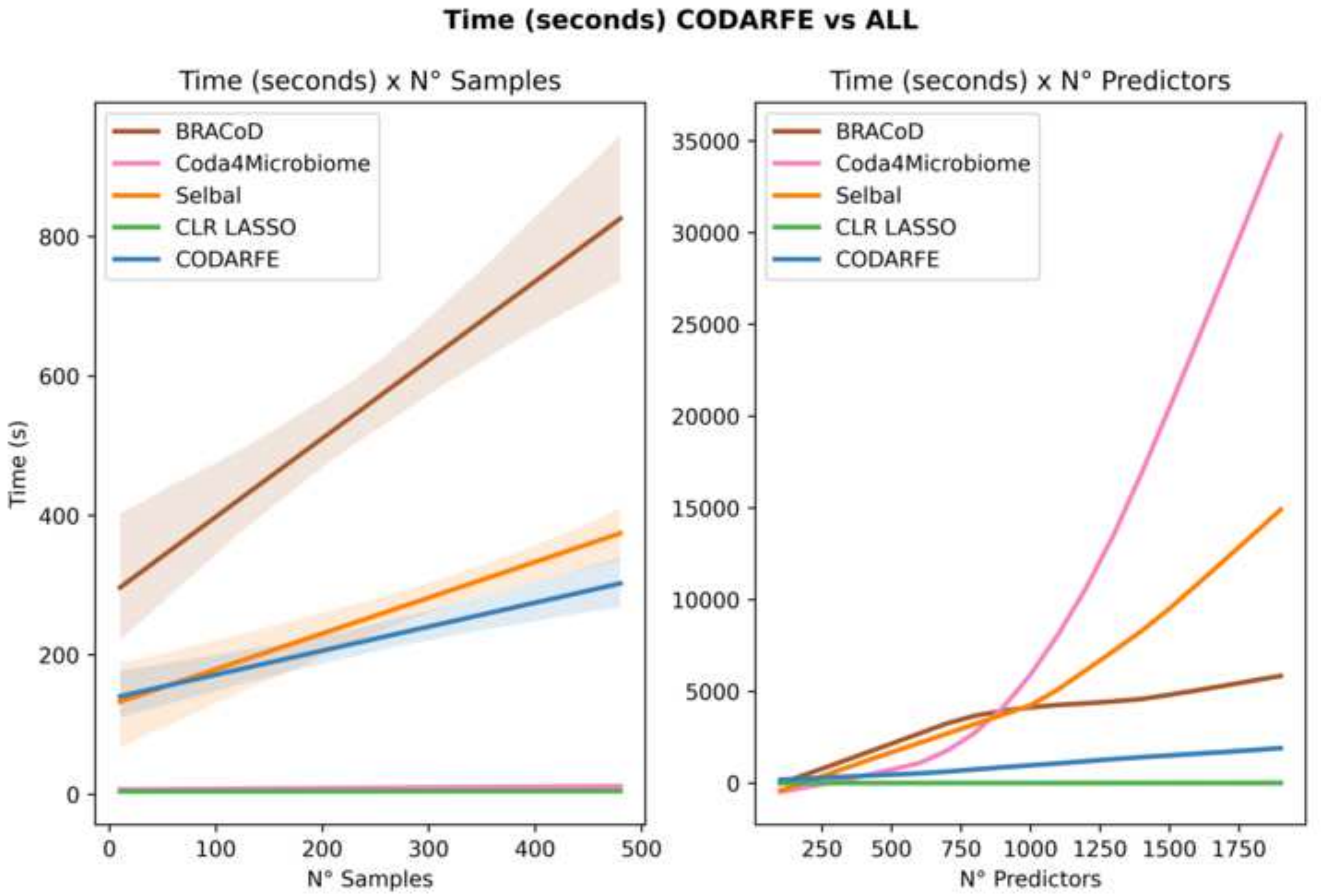
Time (seconds) CODARFE vs ALL
Time (seconds) x N° Samples
BRACoD
Coda4Microbiome
Selbal
CLR LASSO
CODARFE
Time (s)
800
600
400
200
0
0
100
200
300
400
500
N° Samples
Time (seconds) x N° Predictors
35000
30000
25000
20000
15000
10000
5000
0
250
500
750
1000
1250
1500
1750
N° Predictors

Figure4

Click here to access/download;Figure;Fig4.png

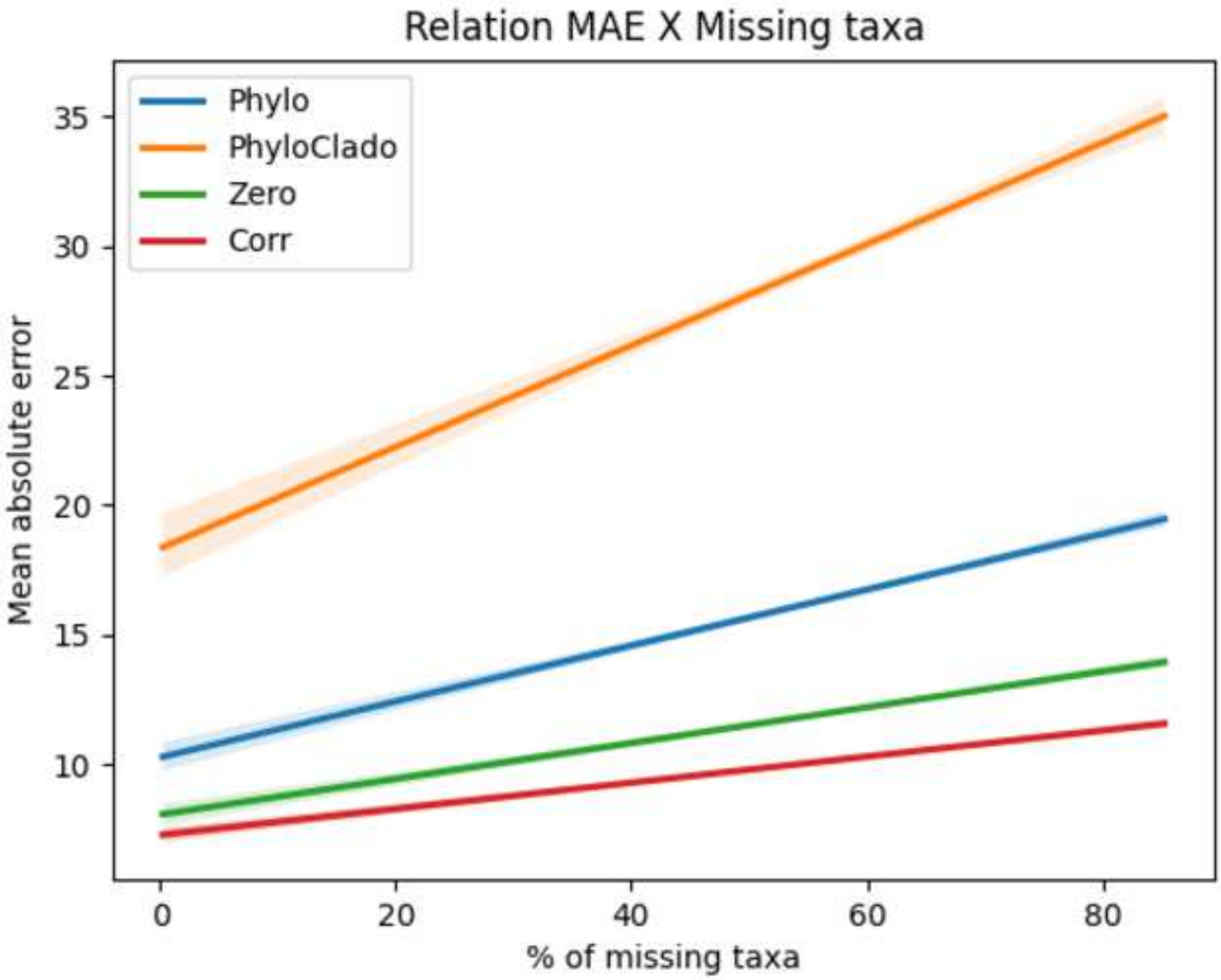

Figure5

Click here to access/download;Figure;Fig5.png

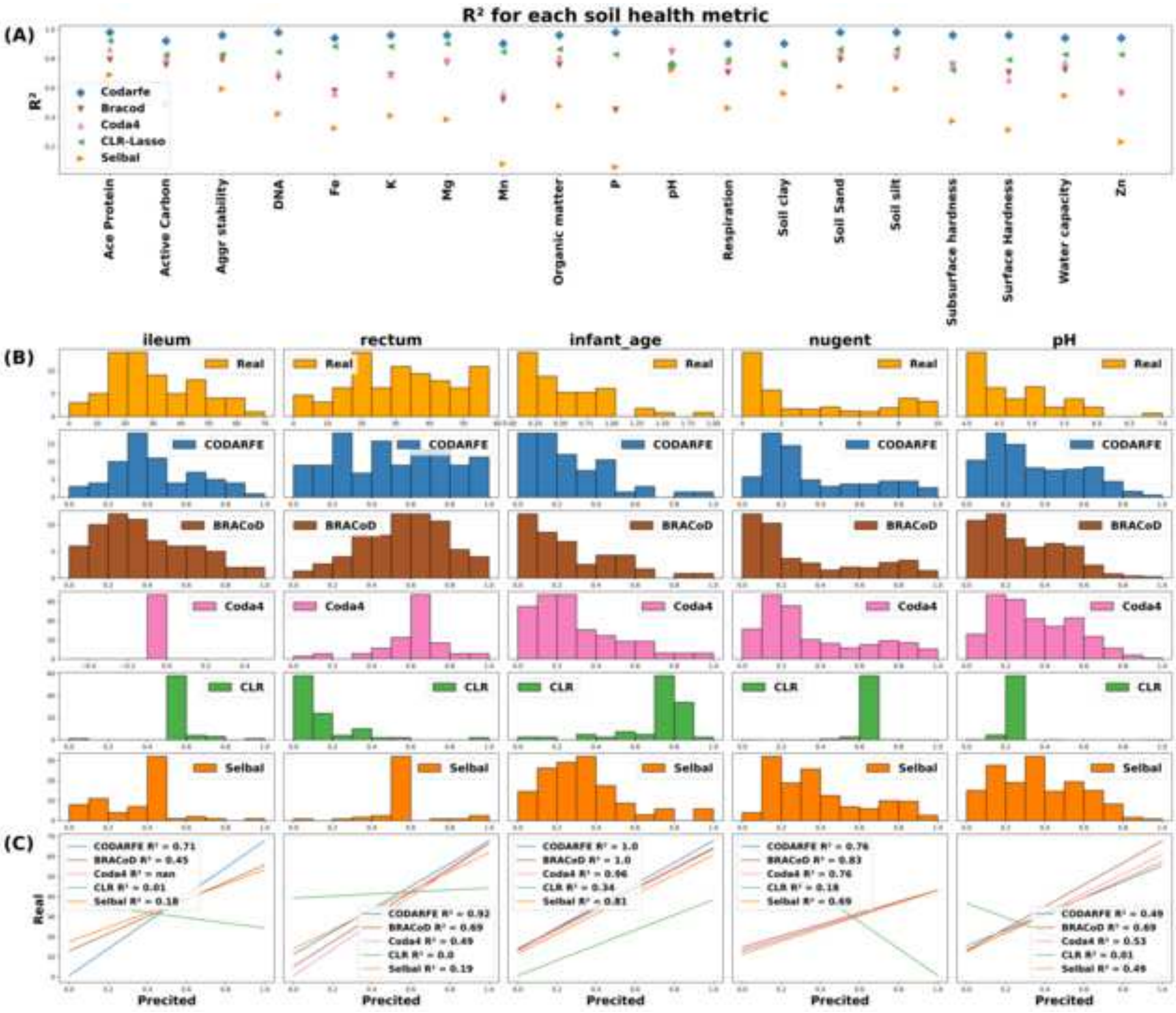

Figure6

Click here to access/download;Figure;Fig6.png

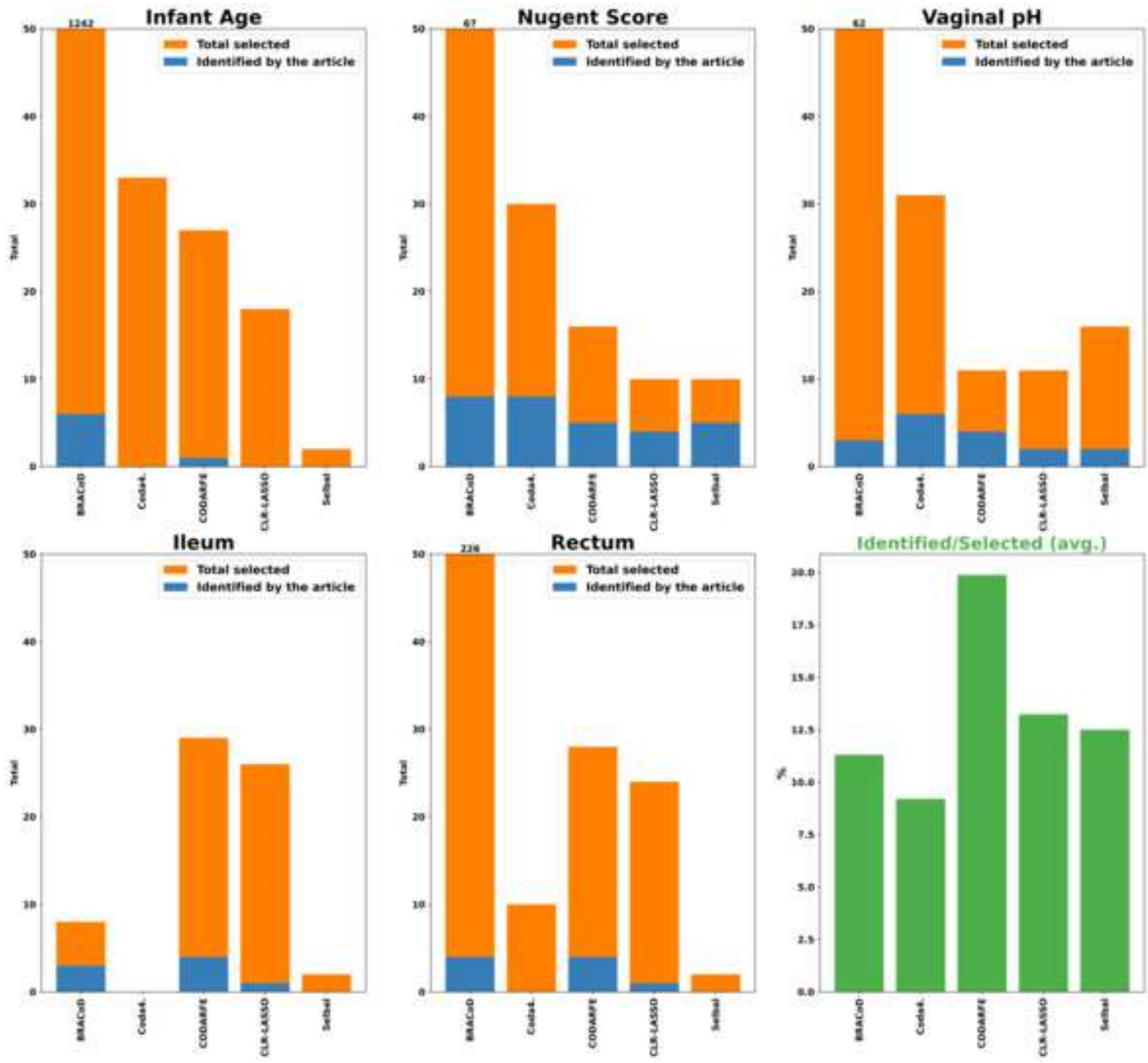

Figure7

Click here to access/download;Figure;Fig7.png

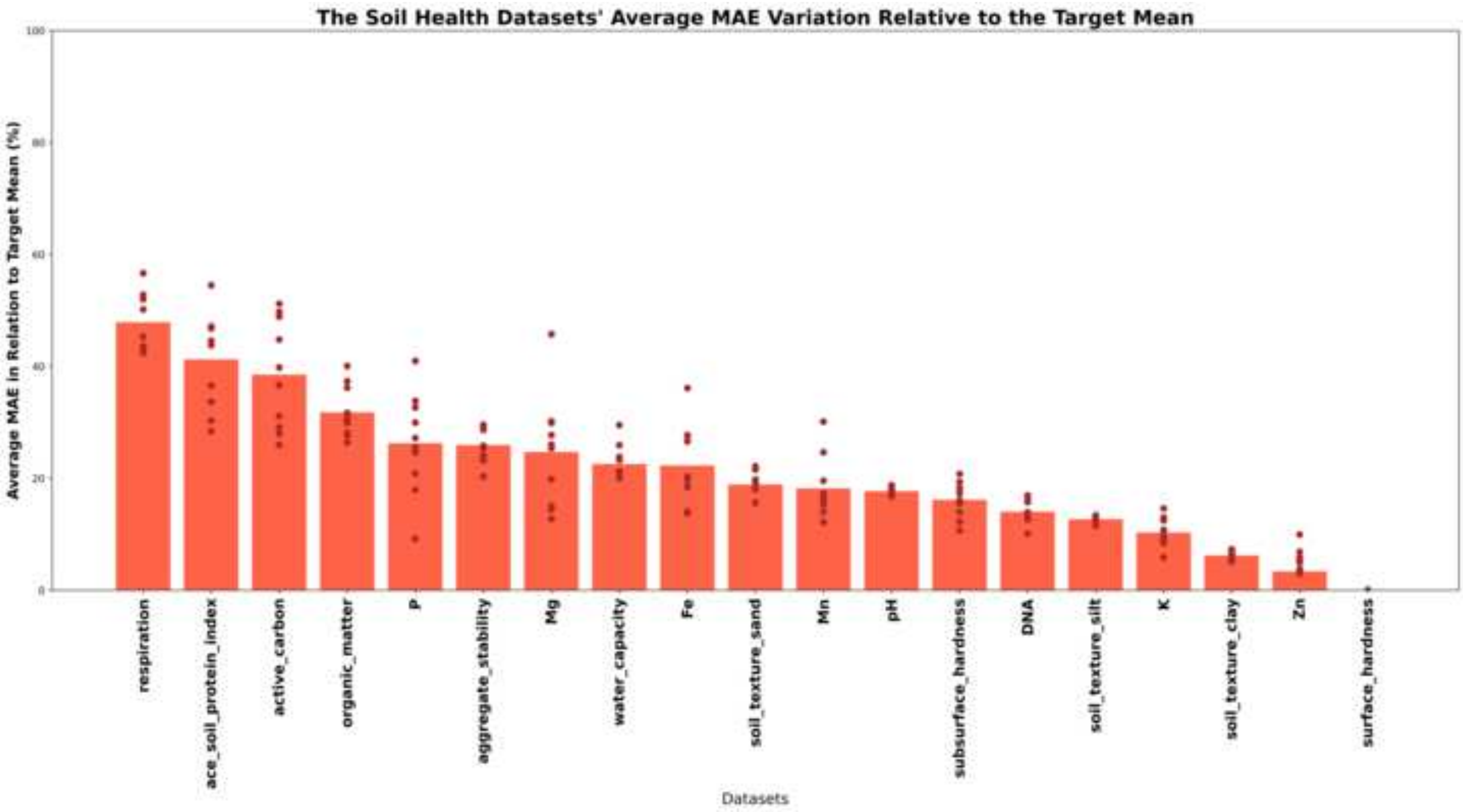

Figure8

Click here to access/download;Figure;Fig8.png

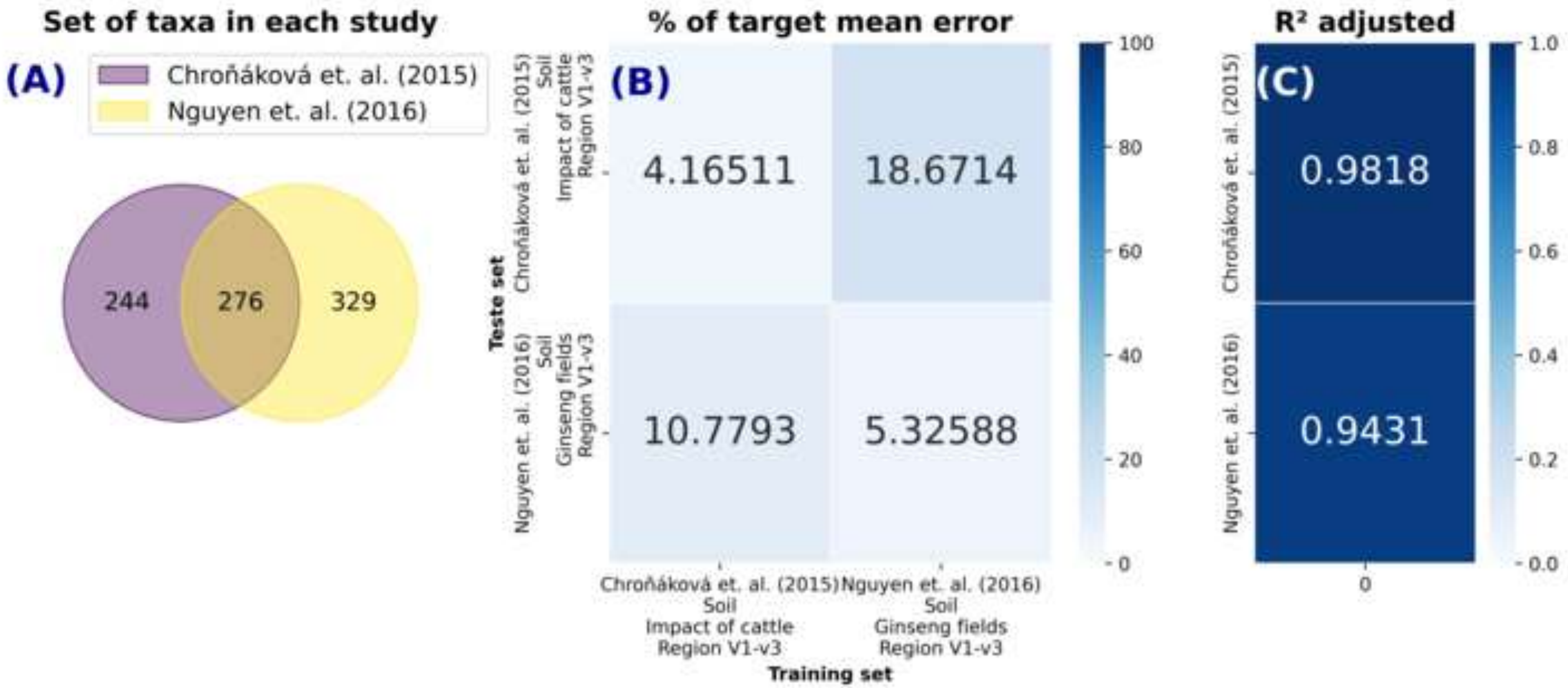

Figure9

Click here to access/download;Figure;Fig9.png

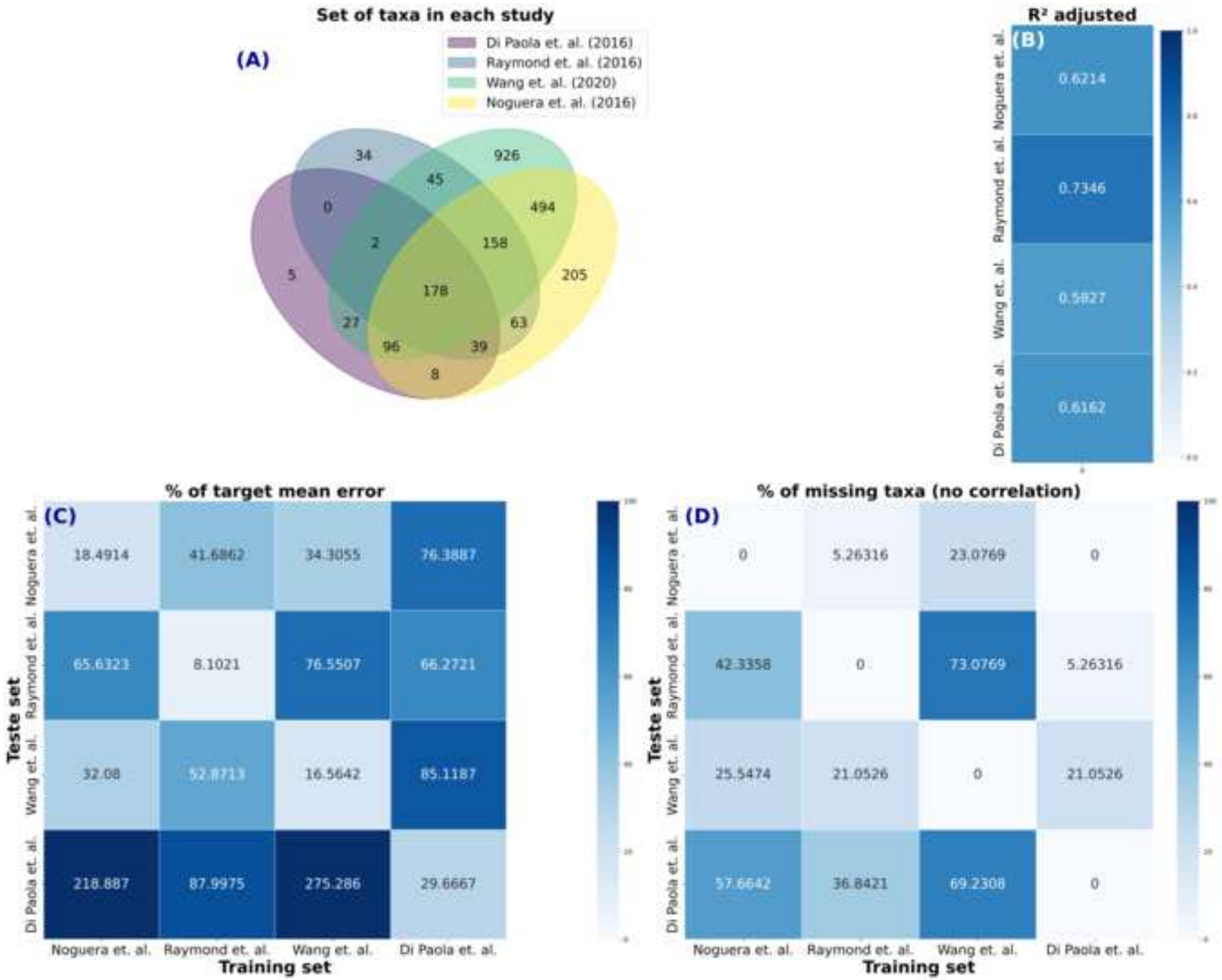

Click here to access/download
Supplementary Material
SUPPLEMENTARY MATERIAL 1 .docx

Click here to access/download
Supplementary Material
SUPPLEMENTARY MATERIAL 2.docx

Click here to access/download
Supplementary Material
SUPPLEMENTARY MATERIAL 3.docx

Click here to access/download
Supplementary Material
Cover letter-review.docx

Graphical Abstract

Click here to access/download;Graphical Abstract;graphical_abstract.docx

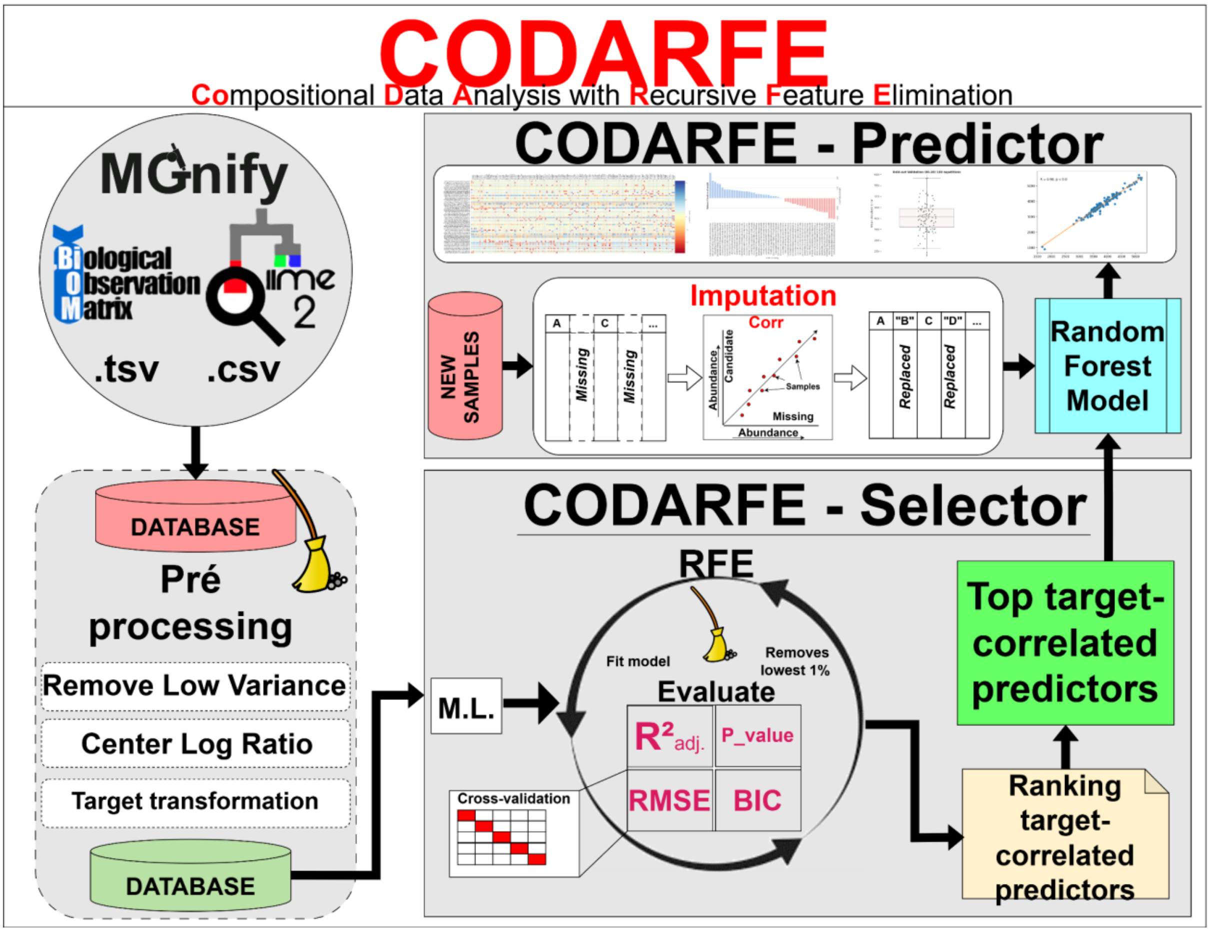

CODARFE's workflow involves several key steps: Initially, a count table from sequencing data is inputted, alongside a metadata table containing the target variable for association. The data undergoes preprocessing to eliminate compositional effects and low-variance predictors. Next, Recursive Feature Elimination (RFE) is applied, iteratively calculating four metrics to refine the predictor selection, ensuring the most relevant subset is identified. This refined predictor set is then used to train a Random-Forest model, enabling target prediction in new samples with a novel imputation method to address missing predictors. Outputs include the predictive model, selected predictors, a heatmap of abundance relative to the target distribution, and a bar chart illustrating individual predictor correlations.
