# Supplementary material for: CODARFE: Unlocking the prediction of continuous environmental variables based on microbiome
Source: Gigascience. 2025 Jun 23;14:giaf055. doi: 10.1093/gigascience/giaf055 (PMC12365963; doi:10.1093/gigascience/giaf055)
Supplement: giaf055_Authors_Response_To_Reviewer_Comments_original_submission [file giaf055_authors_response_to_reviewer_comments_original_submission.pdf]

Author's Response To Reviewer Comments

The complete text of the responses is included below, nonetheless a formatted version can be found in the 'personal cover letter' file.

Dear Dr. Hongling Zhou,
We are grateful for the opportunity to resubmit our manuscript titled "CODARFE: Unlocking the prediction of continuous environmental variables based on microbiome" to GigaScience as a revised manuscript (GIGA-D-24-00556). We have carefully reviewed the insightful feedback provided by the reviewers and have addressed all comments to improve the clarity, applicability, and scientific rigor of our study. The revisions include a more concise discussion, a better explanation of the data sources and their pipelines, and an additional comparison regarding the MaAsLin tool, as requested by one of the reviewers. We hope the responses provided here address all of the reviewers' concerns.
A detailed point-by-point response to the reviewers' comments is included below, highlighting the changes made to the manuscript (changes are highlighted in red). To make it easier to respond, I copied the reviewer's comment in its entirety and then divided the content into separate questions:

Editorial notes:
“In addition, please register any new software application in the bio.tools and SciCrunch.org databases to receive RRID (Research Resource Identification Initiative ID) and biotoolsID identifiers, and include these in your manuscript.”

Response: Thank you for pointing out this issue. We registered as requested: SciCrunch.org ID: SCR_026180; biotools ID: biotools:codarfe; They both are cited in the “Availability of source code and requirements” section.

“Computational workflows should be registered in workflowhub.eu and the DOIs cited in the relevant places in the manuscript. These will facilitate tracking, reproducibility and re-use of your tool.”

Response: It is crucial to clarify that the current version of CODARFE does not qualify as a workflow since it does not use other tools that require the installation of additional packages or the configuration of containers or other similar systems. For this reason, we decided not to register CODARFE on workflowhub.eu. However, we registered it with Zenodo, DOME-ML, GitHub, Biotools, and SciCrunch. If any additional registration is needed, please let us know.

Reviewer #1: “This manuscript addresses key aspects of microbiome data analysis, particularly in relating continuous variables to microbiome data and utilizing microbiome data to predict variables of interest. The data analysis approach is well-articulated; however, there is a notable omission regarding the derivation of the microbiome datasets. While the sources of these datasets are mentioned, it remains unclear whether the authors processed the initial data to produce the count tables used as input or if these tables were directly adopted from the original publications. Given that the data in the main text are derived from studies based on 16S rDNA sequencing, variations in data processing pipelines between publications could introduce significant variability. Although the manuscript discusses the importance of the sequenced 16S rDNA region and the similarity of the environments from which the samples were obtained, it does not address the impact of the initial data processing pipeline (including taxonomy assignment). Additionally, the number of samples in each dataset is not provided in the tables.
The manuscript includes a comparison of the proposed method with other tools; however, it omits MaAsLin

(Microbiome Multivariable Association with Linear Models), that has been applied far more extensively in microbiome data analysis than the tools included in the current manuscript. Incorporating a comparison with MaAsLin would enhance the comprehensiveness of the evaluation."

1) "...there is a notable omission regarding the derivation of the microbiome datasets. While the sources of these datasets are mentioned, it remains unclear whether the authors processed the initial data to produce the count tables used as input or if these tables were directly adopted from the original publications."

Response: We are thankful to you for raising this comment. All the datasets were already count tables, needing no further preprocessing. Except for the cross-studies, where the focus was exactly trying to understand the effect of different pipelines, all the experiments were conducted using splits of the same data for training and testing, making the experiment agnostic to the pipelines used to get the count table. As suggested by the reviewer, we added texts clarifying that no further processes were applied to the datasets added at the end of each dataset group introduction (Group A: lines 102 - 104, Group B: 113 - 115, and Group C: 124 - 126). A final clarification was added at lines 127 - 130.

2) "Given that the data in the main text are derived from studies based on 16S rDNA sequencing, variations in data processing pipelines between publications could introduce significant variability. Although the manuscript discusses the importance of the sequenced 16S rDNA region and the similarity of the environments from which the samples were obtained, it does not address the impact of the initial data processing pipeline (including taxonomy assignment)."

Response: Thank you for bringing this to our attention. An additional point about this worry was brought up in the "Feasibility and limitations" section (lines 654 - 661), and a brief text was included at the conclusion of the cross-studies experiment to describe it better (lines 615 -618). However, the tool's primary objective is to be utilized within a single project, which implies that all samples should be processed using the same pipeline. The inclusion of the two passages in their respective sections clarified this remark.

3) " Additionally, the number of samples in each dataset is not provided in the tables."

Response: Thank you for your valuable suggestion. A table summarizing the number of samples and columns for each dataset was added at the end of supplementary material 1. This table was also referred to at the end of the "Evaluation datasets" section (lines 130 - 131).

4) "The manuscript includes a comparison of the proposed method with other tools; however, it omits MaAsLin (Microbiome Multivariable Association with Linear Models), that has been applied far more extensively in microbiome data analysis than the tools included in the current manuscript. Incorporating a comparison with MaAsLin would enhance the comprehensiveness of the evaluation."

Response: Thank you for your valuable suggestion. We initially considered MaAsLin during the early stages of this work but ultimately decided not to include it, as its primary goal is fundamentally different from that of CODARFE. MaAsLin performs multiple regressions where the covariate (referred to as the "target" in CODARFE) predicts the abundance of taxa, retaining only the most relevant correlations. In contrast, CODARFE aims to identify taxa that can predict the target variable, essentially the reverse approach.
That said, we fully agree that comparing tools would strengthen the manuscript. To address this, we have added a brief explanation of MaAsLin's differences at the end of the "Comparison against four state-of-the-art tools" section (lines 288 - 298). Additionally, we have included a dedicated comparison in Supplementary Material 4. Given the fundamental methodological differences, integrating this comparison into the main results could complicate their interpretation, so we believe this supplementary analysis provides the best clarity.

Reviewer #2: "The authors propose a novel tool called CODARFE for sparse compositional microbiome-predictors selection and prediction of continuous environmental factors. The methodology described in the

paper is easy to understand. The numerical results described in the paper suggest that the proposed tool performs well in a variety of scenarios considered by the authors. However, my major concern with the paper is I had difficulty reading the legends in many of the figures. Some figures are very poor quality. Secondly, the results and discussion sections need to be written more concisely and clearly. Due to these reasons, I had difficulty appreciating the contributions of the paper."

1) "... my major concern with the paper is I had difficulty reading the legends in many of the figures. Some figures are very poor quality."

Response: We appreciate your careful attention to detail and apologize for the image quality. For clarification, the images are uploaded separately as one file per image in at least 300 dpi (all the plots are in 600 dpi). When the journal system generates the PDF file used for the revision, the images appear to be of lower quality. To solve this problem, we also provide another source for the reviewer to download all the images by this link here: 10.6084/m9.figshare.28414172.
Regarding the legends, the font size was increased from 6 to 8 for all images and tables.

2) "Secondly, the results and discussion sections need to be written more concisely and clearly."

Response: We appreciate you bringing up this point. We sincerely regret that some of the discussion was unclear at some points. We reworked several pontual questions in all the subsections making it more concise and clear.

Additional changes

During the revision, we noticed that some of the citations were not in the correct order. After correction, they now conform to the journal's standards.

We thank you for receiving our manuscript and considering it for review. We appreciate your time and look forward to receiving your response.

Sincerely,

Msc. Murilo C. Barbosa1, Dr. João F. M. da Silva, Dr. Leonardo C. Alves, Dr. Robert D Finn, and Dr. Alexandre R. Paschoal

Editorial Manager®

Cookie Preference Center

We use cookies which are necessary to make our site work. We may also use additional cookies to analyze, improve and personalize our content and your digital experience. For more information, see our Cookie Policy.

You may choose not to allow some types of cookies. However, blocking some types may impact your experience of our site and the services we are able to offer. See the different category headings below to find out more or change your settings.

Allow all

Manage Consent Preferences

Strictly Necessary Cookies

Always active

These cookies are necessary for the website to function and cannot be switched off in our systems. They are usually only set in response to actions made by you which amount to a request for services, such as setting your privacy preferences, logging in or filling in forms. You can set your browser to block or alert you about these cookies, but some parts of the site will not then work. These cookies do not store any personally identifiable information.

Functional Cookies

These cookies enable the website to provide enhanced functionality and personalisation. They may be set by us or by third party providers whose services we have added to our pages. If you do not allow these cookies then some or all of these services may not function properly.

Performance Cookies

These cookies allow us to count visits and traffic sources so we can measure and improve the performance of our site. They help us to know which pages are the most and least popular and see how visitors move around the site.

Targeting Cookies

These cookies may be set through our site by our advertising partners. They may be used by those companies to build a profile of your interests and show you relevant adverts on other sites. If you do not allow these cookies, you will experience less targeted advertising.

Cookie List

Clear

Apply Cancel
Consent Leg.Interest

Confirm my choices

Powered by onetrus
