# Supplementary material for: CODARFE: Unlocking the prediction of continuous environmental variables based on microbiome
Source: Gigascience. 2025 Jun 23;14:giaf055. doi: 10.1093/gigascience/giaf055 (PMC12365963; doi:10.1093/gigascience/giaf055)
Supplement: giaf055_Authors_Response_To_Reviewer_Comments_Revision_1 [file giaf055_authors_response_to_reviewer_comments_revision_1.pdf]

# Author's Response To Reviewer Comments

Dear Dr. Hongling Zhou,

We are grateful for the opportunity to resubmit our manuscript titled "CODARFE: Unlocking the prediction of continuous environmental variables based on microbiome" to GigaScience as a revised manuscript for this minor review. We have carefully reviewed the feedback provided by the reviewers and have addressed all comments to improve the clarity, applicability, and scientific rigor of our study.
Regarding the MaAsLin3 issue, we decided to remove it from the manuscript, since it is not an appropriate tool in the present context, as pointed out by the second reviewer.

A detailed point-by-point response to the reviewers' comments is included below. To make it easier to respond, I copied the reviewer's comment in its entirety and then divided the content into separate questions:

-------------------------------------------------------------------------------------------------------------------------------------------------------------------------

Reviewer #1: " I am satisfied with the revised manuscript. Please check the heading of Table S2, as the data in this table cannot refer to soil health metrics."

1) "Please check the heading of Table S2, as the data in this table cannot refer to soil health metrics."

Response: We appreciate your careful attention to detail and apologize for the mistake. The table S2 indeed refers to the human health targets and not the soil health metrics. The text was changed.

-------------------------------------------------------------------------------------------------------------------------------------------------------------------------

Reviewer #2: "The authors have done a nice job with the revision of their paper. Unfortunately, the figures are still very poor quality (for my eyes anyway). The fonts and images are still very blurred. I have the following additional comment.

I was surprised that the authors decided to include MaAsLin3 in their analysis especially in light of what they wrote on page 16 (lines 289 to 297). If anything, the authors should have included MirKAT which might be more appropriate in the present context. For a reader it looks odd why the authors considered MaAsLin3 when they acknowledge that it is not an appropriate tool in the present context. It looks strange to me.

I think the authors may remove the following sentence in the last page of Supplementary Material 4.

"By this manner, the conclusion is that MaAsLin3 is a very helpful and trustworthy tool but with an application different from the ones compared in the main text. Meaning that"

The above sentence is loosely put as the authors have not demonstrated (at least to me) in their analysis that MaAslin3 is "very helpful and trustworthy tool"."

1) "Unfortunately, the figures are still very poor quality (for my eyes anyway). The fonts and images are still very blurred."

Response: We appreciate your careful attention to detail and apologize for the image quality. For clarification, the images are uploaded separately as one file per image in at least 300 dpi (all the plots are in 600 dpi). When the journal system generates the PDF file used for the revision, the images appear to be

of lower quality. On the top right corner of each individual image page there will be a link similar to this one:
“Click here to access/download;Figure;FigX.png”, after clicking on it, the full resolution image will be downloaded to your computer. If you still can’t see the images on its full resolution, we also provided another source for the reviewer to download all the images on this link: 10.6084/m9.figshare.28414172.

2) “I was surprised that the authors decided to include MaAsLin3 in their analysis especially in light of what they wrote on page 16 (lines 289 to 297). If anything, the authors should have included MirKAT which might be more appropriate in the present context. For a reader it looks odd why the authors considered MaAsLin3 when they acknowledge that it is not an appropriate tool in the present context. It looks strange to me.”

Response: We appreciate you bringing up this point. We agree that the comparison looks odd between the other tools. For this reason, we decided to remove MaAsLin3 from the manuscript.

3) “I think the authors may remove the following sentence in the last page of Supplementary Material 4.
"By this manner, the conclusion is that MaAsLin3 is a very helpful and trustworthy tool but with an application different from the ones compared in the main text. Meaning that"
The above sentence is loosely put as the authors have not demonstrated (at least to me) in their analysis that MaAslin3 is "very helpful and trustworthy tool".” ”

Response: Thank you for your valuable suggestion. Since we removed MaAsLin3 from the manuscript, there will no longer be a Supplementary Material 4.

------------------------------------------------------------------------------------------------------------------------------------------------------------------------

We thank you for receiving our manuscript and considering it for review. We appreciate your time and look forward to receiving your response.

Sincerely,

Msc. Murilo C. Barbosa, Dr. João F. M. da Silva, Dr. Leonardo C. Alves, Dr. Robert D Finn, and Dr. Alexandre R. Paschoal

Editorial Manager®

## Cookie Preference Center

We use cookies which are necessary to make our site work. We may also use additional cookies to analyze, improve and personalize our content and your digital experience. For more information, see our Cookie Policy.

You may choose not to allow some types of cookies. However, blocking some types may impact your experience of our site and the services we are able to offer. See the different category headings below to find out more or change your settings.

Allow all

## Manage Consent Preferences

### Strictly Necessary Cookies

**Always active**

These cookies are necessary for the website to function and cannot be switched off in our systems. They are usually only set in response to actions made by you which amount to a request for services, such as setting your privacy preferences, logging in or filling in forms. You can set your browser to block or alert you about these cookies, but some parts of the site will not then work. These cookies do not store any personally identifiable information.

### Functional Cookies

These cookies enable the website to provide enhanced functionality and personalisation. They may be set by us or by third party providers whose services we have added to our pages. If you do not allow these cookies then some or all of these services may not function properly.

### Performance Cookies

These cookies allow us to count visits and traffic sources so we can measure and improve the performance of our site. They help us to know which pages are the most and least popular and see how visitors move around the site.

### Targeting Cookies

These cookies may be set through our site by our advertising partners. They may be used by those companies to build a profile of your interests and show you relevant adverts on other sites. If you do not allow these cookies, you will experience less targeted advertising.

## Cookie List

Clear

Apply Cancel

Consent Leg.Interest

Confirm my choices

Powered by onetrust
