# Supplementary material for: CODARFE: Unlocking the prediction of continuous environmental variables based on microbiome
Source: Gigascience. 2025 Jun 23;14:giaf055. doi: 10.1093/gigascience/giaf055 (PMC12365963; doi:10.1093/gigascience/giaf055)
Supplement: giaf055_Reviewer_1_Report_Original_submission — Jaak Truu -- 1/30/2025 [file giaf055_reviewer_1_report_original_submission.pdf]

Reviewer Report

Title: CODARFE: Unlocking the prediction of continuous environmental variables based on microbiome

Version: Original Submission **Date:** 1/30/2025

Reviewer name: Jaak Truu

Reviewer Comments to Author:

This manuscript addresses key aspects of microbiome data analysis, particularly in relating continuous variables to microbiome data and utilizing microbiome data to predict variables of interest. The data analysis approach is well-articulated; however, there is a notable omission regarding the derivation of the microbiome datasets. While the sources of these datasets are mentioned, it remains unclear whether the authors processed the initial data to produce the count tables used as input or if these tables were directly adopted from the original publications. Given that the data in the main text are derived from studies based on 16S rDNA sequencing, variations in data processing pipelines between publications could introduce significant variability. Although the manuscript discusses the importance of the sequenced 16S rDNA region and the similarity of the environments from which the samples were obtained, it does not address the impact of the initial data processing pipeline (including taxonomy assignment). Additionally, the number of samples in each dataset is not provided in the tables.
The manuscript includes a comparison of the proposed method with other tools; however, it omits MaAsLin (Microbiome Multivariable Association with Linear Models), that has been applied far more extensively in microbiome data analysis than the tools included in the current manuscript. Incorporating a comparison with MaAsLin would enhance the comprehensiveness of the evaluation.

Methods

Are the methods appropriate to the aims of the study, are they well described, and are necessary controls included? Choose an item.

Conclusions

Are the conclusions adequately supported by the data shown? Choose an item.

Reporting Standards

Does the manuscript adhere to the journal's guidelines on minimum standards of reporting? Choose an item.

Choose an item.

Statistics

Are you able to assess all statistics in the manuscript, including the appropriateness of statistical tests used? Choose an item.

Quality of Written English

Please indicate the quality of language in the manuscript: Choose an item.

Declaration of Competing Interests

Please complete a declaration of competing interests, considering the following questions:

- Have you in the past five years received reimbursements, fees, funding, or salary from an organisation that may in any way gain or lose financially from the publication of this manuscript, either now or in the future?
- Do you hold any stocks or shares in an organisation that may in any way gain or lose financially from the publication of this manuscript, either now or in the future?
- Do you hold or are you currently applying for any patents relating to the content of the manuscript?
- Have you received reimbursements, fees, funding, or salary from an organization that holds or has applied for patents relating to the content of the manuscript?
- Do you have any other financial competing interests?
- Do you have any non-financial competing interests in relation to this paper?

If you can answer no to all of the above, write 'I declare that I have no competing interests' below. If your reply is yes to any, please give details below.

I declare that I have no competing interests

I agree to the open peer review policy of the journal. I understand that my name will be included on my report to the authors and, if the manuscript is accepted for publication, my named report including any attachments I upload will be posted on the website along with the authors' responses. I agree for my report to be made available under an Open Access Creative Commons CC-BY license (http://creativecommons.org/licenses/by/4.0/). I understand that any comments which I do not wish to be included in my named report can be included as confidential comments to the editors, which will not be published.

Choose an item.

To further support our reviewers, we have joined with Publons, where you can gain additional credit to further highlight your hard work (see: https://publons.com/journal/530/gigascience). On publication of this paper, your review will be automatically added to Publons, you can then choose whether or not to claim your Publons credit. I understand this statement.

Yes Choose an item.
