# Supplementary material for: CODARFE: Unlocking the prediction of continuous environmental variables based on microbiome
Source: Gigascience. 2025 Jun 23;14:giaf055. doi: 10.1093/gigascience/giaf055 (PMC12365963; doi:10.1093/gigascience/giaf055)
Supplement: giaf055_Reviewer_2_Report_Revision_1 — Reviewer 2 -- 3/28/2025 [file giaf055_reviewer_2_report_revision_1.pdf]

**Reviewer Report**

**Title: CODARFE: Unlocking the prediction of continuous environmental variables based on microbiome**

**Version: Revision 1** **Date:** 3/28/2025

**Reviewer name: Reviewer 2**

**Reviewer Comments to Author:**

The authors have done a nice job with the revision of their paper. Unfortunately, the figures are still very poor quality (for my eyes anyway). The fonts and images are still very blurred. I have the following additional comment.
I was surprised that the authors decided to include MaAsLin3 in their analysis especially in light of what they wrote on page 16 (lines 289 to 297). If anything, the authors should have included MirKAT which might be more appropriate in the present context. For a reader it looks odd why the authors considered MaAsLin3 when they acknowledge that it is not an appropriate tool in the present context. It looks strange to me.
I think the authors may remove the following sentence in the last page of Supplementary Material 4.
"By this manner, the conclusion is that MaAsLin3 is a very helpful and trustworthy tool but with an application different from the ones compared in the main text. Meaning that"
The above sentence is loosely put as the authors have not demonstrated (at least to me) in their analysis that MaAslin3 is "very helpful and trustworthy tool".

**Methods**

Are the methods appropriate to the aims of the study, are they well described, and are necessary controls included? Choose an item.

**Conclusions**

Are the conclusions adequately supported by the data shown? Choose an item.

**Reporting Standards**

Does the manuscript adhere to the journal’s guidelines on [minimum standards of reporting?]() Choose an item.

Choose an item.

**Statistics**

Are you able to assess all statistics in the manuscript, including the appropriateness of statistical tests used? Choose an item.

**Quality of Written English**

Please indicate the quality of language in the manuscript: Choose an item.

**Declaration of Competing Interests**

Please complete a declaration of competing interests, considering the following questions:

- Have you in the past five years received reimbursements, fees, funding, or salary from an organisation that may in any way gain or lose financially from the publication of this manuscript, either now or in the future?
- Do you hold any stocks or shares in an organisation that may in any way gain or lose financially from the publication of this manuscript, either now or in the future?
- Do you hold or are you currently applying for any patents relating to the content of the manuscript?
- Have you received reimbursements, fees, funding, or salary from an organization that holds or has applied for patents relating to the content of the manuscript?
- Do you have any other financial competing interests?
- Do you have any non-financial competing interests in relation to this paper?

If you can answer no to all of the above, write 'I declare that I have no competing interests' below. If your reply is yes to any, please give details below.

I declare that I have not competing interests

I agree to the open peer review policy of the journal. I understand that my name will be included on my report to the authors and, if the manuscript is accepted for publication, my named report including any attachments I upload will be posted on the website along with the authors' responses. I agree for my report to be made available under an Open Access Creative Commons CC-BY license (http://creativecommons.org/licenses/by/4.0/). I understand that any comments which I do not wish to be included in my named report can be included as confidential comments to the editors, which will not be published.

Choose an item.

To further support our reviewers, we have joined with Publons, where you can gain additional credit to further highlight your hard work (see: https://publons.com/journal/530/gigascience). On publication of this paper, your review will be automatically added to Publons, you can then choose whether or not to claim your Publons credit. I understand this statement.

Yes Choose an item.
